# Sarcopenia-related traits and coronary artery disease: a bi-directional Mendelian randomization study

**DOI:** 10.18632/aging.102815

**Published:** 2020-02-16

**Authors:** Hui-Min Liu, Qiang Zhang, Wen-Di Shen, Bo-Yang Li, Wan-Qiang Lv, Hong-Mei Xiao, Hong-Wen Deng

**Affiliations:** 1 Center for System Biology, Data Sciences, and Reproductive Health, School of Basic Medical Science, Central South University, Yuelu, Changsha, P.R. China; 2 College of Public Health, Zhengzhou University, High-Tech Development Zone of States, Zhengzhou, P.R. China; 3 Tulane Center of Bioinformatics and Genomics, Department of Biostatistics and Data Science, Tulane University School of Public Health and Tropical Medicine, New Orleans, LA 70112, USA

**Keywords:** Mendelian randomization, sarcopenia, coronary artery disease

## Abstract

Previous Mendelian randomization (MR) studies have yielded a conflicting causal relationship between sarcopenia and coronary artery disease (CAD), and lack the association of CAD with sarcopenia. We performed a bi-directional MR approach to clarify the causality and causal direction between sarcopenia-related traits and CAD. In stage 1 analysis, estimates of inverse variance weighting (IVW) and several sensitivity analyses were obtained by applying genetic variants that predict sarcopenia-related traits to CAD. Conversely, we also applied genetic variants that predict CAD to sarcopenia-related traits in stage 2 analyses. IVW analysis showed that higher handgrip strength reduces risk for CAD: A 1-kilogram (kg) increase in genetically determined left handgrip strength reduced odds of CAD by 36% [odds ratio (OR) = 0.64, 95% confidence interval (CI) 0.498 - 0.821, *p* = 4.56E-04], and right handgrip strength reduced odds of CAD by 41.1% (OR = 0.599, 95% CI 0.476 - 0.753, *p* = 1.10E-05). However, genetically predicted CAD did not show any causal association with handgrip strength, and no significant causal relationship was detected between genetically instrumented body lean mass and CAD. Our results suggest that decreased muscle strength but not decreased muscle mass leads to the increased risk of CAD in sarcopenia.

## INTRODUCTION

Sarcopenia is a common clinical condition characterized by reduced muscle mass and muscle strength with aging [1]. Morbidity and mortality from cardiovascular disease (CVD) are increasing worldwide due to aging [2]. Coronary artery disease (CAD) is the most common form of CVD and a major global health problem that imposes a great economic burden [2]. A series of observational studies have examined the association between sarcopenia and CVD; however, no consensus has been reached [3–6]. A recent meta-analysis study illustrated that handgrip strength was an independent predictor of CVD in community-dwelling populations [3], but this association disappeared after adjusting for baseline CVD risk factors in another prospective cohort study [4]. Furthermore, reverse association between muscle mass and CAD has also been observed in traditional studies [5, 6]. A possible mechanism underlying these contradictory results might be the association between both CAD and sarcopenia with high body fat mass [7, 8] and other unmeasured potential confounders. Less muscle mass reduces total energy expenditure, which might lead to higher fat mass and a subsequent increase in risk of CAD. On the other hand, accumulated body fat mass induces chronic inflammation, which further contributes to the development and progression of sarcopenia and CAD [7–9].

Mendelian randomization (MR) analysis has been widely performed to investigate the potential causality between genetically predicted environmental factors and diseases independent of the factors that might confound observational studies [10–12]. MR studies have also yielded a conflicting causal relationship between sarcopenia-related traits (handgrip strength) and CVD [13–16]. A two-sample MR (TSMR) study reported that increased handgrip strength was causally related to lower risk of CAD [13], and similar results were also obtained between handgrip strength and CVD in UK individuals with one-sample MR analysis [15, 16]. However, another TSMR analysis did not find causal evidence between handgrip strength and CAD [14]. To our knowledge, no MR analysis has been conducted between muscle mass and CAD, nor was the MR study focused on the association of CAD on sarcopenia. Therefore, we performed a bi-directional MR approach to clarify the causality and causal direction between sarcopenia-related traits (muscle mass and muscle strength) and CAD.

## RESULTS

### Stage 1: Influence of genetically predicted sarcopenia-related traits on CAD

In total, we obtained 399, 81, and 95 linkage disequilibrium (LD)-independent (*r*^2^ < 0.001) instrumental variables (IVs) that achieved genome-wide significance level (*p* < 5×10^−8^) from body lean mass, left handgrip strength, and right handgrip strength, respectively (Supplementary Table 1). *F* statistics are presented to demonstrate the strength of selected IVs. The *F* statistics for selected IVs and the variance explained by them for body lean mass, left handgrip strength and right handgrip strength were 81.02 and 8.9%, 50.34 and 1.1%, and 46.55 and 1.3%, respectively. *F* statistics were larger than 10, indicating that selected IVs were powerful enough to mitigate any potential bias of the causal IVs estimate. The negative control analysis results showed that body lean mass, left handgrip strength and right handgrip strength were not related to myopia, indicating the selected IVs of exposures were appropriate (Supplementary Tables 4–6).

As the heterogeneity test showed significant heterogeneity among selected IVs (*p* < 0.05, Table 1), inverse variance weight (IVW) in a random-effects model was used. IVW analysis showed that higher handgrip strength reduces risk for CAD: A 1-kg increase in genetically determined left handgrip strength reduced odds of CAD by 36% [odds ratio (OR) = 0.640, 95% confidence interval (CI) 0.498- 0.821, *p* = 4.56E-04], and a 1-kg increase in genetically determined right handgrip reduced odds of CAD by 41.1% (OR = 0.599, 95% CI 0.476- 0.753, *p* = 1.10E-05) (Table 1). The MR-Egger analysis did not detect any pleiotropy for the selected IVs (left handgrip strength, intercept = 0.008, *p* = 0.307; right handgrip strength, intercept = 0.004, *p* = 0.524) (Table 1). Consistent with the IVW results, all sensitivity analyses identified significant causal effect of handgrip strength on CAD (Tables 2 and 3).

**Table 1:** Association between sarcopenia-related traits and CAD using MR Egger and IVW analysis.

**Exposures**	**Outcomes**	**IVs selection ^a^**	**No. of IVs**	**Heterogeneity test**	**MR Egger**	**IVW (random-effect model)**	
				**Cochran's Q (*p*)**	**Intercept (*p*)**	**OR (95% CI)**	**p**
Body lean mass	CAD	All	399	842.005 (<0.001)	-0.001 (0.65)	0.929 (0.834, 1.033)	0.170
		Removed 1	398	830.824 (<0.001)	-0.001 (0.548)	0.924 (0.831, 1.027)	0.144
		Removed 2	387	805.502 (<0.001)	0 (0.957)	0.901 (0.808, 1.005)	0.061
Left handgrip strength	CAD	All	81	156.954 (<0.001)	0.008 (0.307)	0.640 (0.498, 0.821)	4.56E-04*
		Removed 1	79	149.928 (<0.001)	0.007 (0.330)	0.645 (0.502, 0.829)	0.001*
		Removed 2	76	148.771 (<0.001)	0.009 (0.284)	0.647 (0.498, 0.841)	0.001*
Right handgrip strength	CAD	All	95	180.1231 (<0.001)	0.004 (0.524)	0.599 (0.476, 0.753)	1.10E-05*
		Removed 1	93	173.892 (<0.001)	0.004 (0.513)	0.606 (0.482, 0.762)	0*
		Removed 2	91	172.782 (<0.001)	0.006 (0.408)	0.604 (0.477, 0.765)	0*
CAD	Body lean mass	All	39	898.685 (<0.001)	0.003 (0.366)	0.992 (0.963, 1.023)	0.618
		Removed 1	36	893.680 (<0.001)	0.003 (0.379)	0.992 (0.976, 1.007)	0.616
CAD	Left handgrip strength	All	39	110.459 (<0.001)	0 (0.913)	0.997 (0.985, 1.009)	0.642
		Removed 1	36	109.932 (<0.001)	0 (0.886)	0.997 (0.99, 1.003)	0.62
CAD	Right handgrip strength	All	39	102.492 (<0.001)	-0.001 (0.695)	0.996 (0.984, 1.008)	0.531
		Removed 1	36	101.680 (<0.001)	0 (0.667)	0.996 (0.99, 1.002)	0.505

^a^‘All’ represents analyses with all selected IVs; ‘Removed 1’ represents analyses after removing proxy IVs; ‘Removed 2’ represents analyses after removing potential pleiotropic SNPs (related to body fat mass and height).

Bonferroni corrected significance level (0.05/3 = 0.016) was used to correct for multiple comparisons.

*p<0.016.

CAD: coronary artery disease; MR: mendelian randomization; IVW: inverse variance weighted; IVs: instrumental variables; CI: confidence interval; OR: odds ratio.

**Table 2:** Association between sarcopenia-related traits and CAD using weighted median and RAPS analysis.

**Exposures**	**Outcomes**	**IVs selection ^a^**	**No. of IVs**	**Weighted median**		**RAPS**	
				**OR (95% CI)**	**p**	**OR (95% CI)**	**p**
Body lean mass	CAD	All	399	0.961 (0.84, 1.097)	0.554	0.919 (0.827, 1.019)	0.110
		Removed 1	398	0.959 (0.844, 1.091)	0.524	0.916 (0.825, 1.015)	0.095
		Removed 2	387	0.941 (0.827, 1.07)	0.356	0.89 (0.8, 0.991)	0.033
Left handgrip strength	CAD	All	81	0.631 (0.47, 0.849)	0.002*	0.642 (0.493, 0.836)	0.001*
		Removed 1	79	0.614 (0.461, 0.817)	0.001*	0.649 (0.498, 0.846)	0.001*
		Removed 2	76	0.638 (0.478, 0.85)	0.002*	0.645 (0.489, 0.851)	0.002*
Right handgrip strength	CAD	All	95	0.61 (0.466, 0.799)	3.32E-04*	0.593 (0.466, 0.757)	2.55E-05*
		Removed 1	93	0.613 (0.470, 0.800)	0*	0.6 (0.471, 0.764)	0*
		Removed 2	91	0.673 (0.515, 0.880)	0.004*	0.592 (0.46, 0.76)	0*
CAD	Body lean mass	All	39	0.998 (0.987, 1.008)	0.685	0.988 (0.968, 1.008)	0.234
		Removed 1	36	0.997 (0.992, 1.002)	0.563	0.986 (0.976, 0.996)	0.199
CAD	Left handgrip strength	All	39	0.995 (0.983, 1.007)	0.390	0.994 (0.983, 1.005)	0.261
		Removed 1	36	0.995 (0.989, 1)	0.382	0.993 (0.987, 0.999)	0.232
CAD	Right handgrip strength	All	39	1.002 (0.99, 1.014)	0.751	0.993 (0.982, 1.004)	0.218
		Removed 1	36	1.001 (0.995, 1.007)	0.877	0.992 (0.987, 0.998)	0.188

^a^‘All’ represents analyses with all selected IVs; ‘Removed 1’ represents analyses after removing proxy IVs; ‘Removed 2’ represents analyses after removing potential pleiotropic SNPs (related to body fat mass and height).

Bonferroni corrected significance level (0.05/3 = 0.016) was used to correct for multiple comparisons.

*p<0.016.

CAD: coronary artery disease; RAPS: robust adjusted profile score; IVs: instrumental variables; CI: confidence interval; OR: odds ratio.

**Table 3:** Association between sarcopenia-related traits and CAD using MR-PRESSO analysis.

**Exposures**	**Outcomes**	**IVs selection ^a^**	**No. of IVs**	**MR-PRESSO**		
				**OR (95% CI)**	**p**	**IVs outliers**
Body lean mass	CAD	All	399	0.919 (0.834, 1.012)	0.0866	rs11065979, rs216193, rs2289976, rs2678204, rs28391281, rs3843751, rs66922415, rs7097872, rs7781964
		Removed 1	398	0.996 (0.986, 1.007)	0.494	rs11065979, rs216193, rs2289976, rs2678204, rs28391281, rs3843751, rs66922415, rs7097872, rs7781964
		Removed 2	387	0.894 (0.808, 0.989)	0.030	rs11065979, rs216193, rs2678204, rs28391281, rs3843751, rs66922415, rs7097872
Left handgrip strength	CAD	All	81	0.663 (0.521, 0.843)	0.001*	rs11130333
		Removed 1	79	0.669 (0.526, 0.852)	0.002*	rs11130333
		Removed 2	76	0.672 (0.523, 0.865)	0.003*	rs11130333
Right handgrip strength	CAD	All	95	0.599 (0.476, 0.753)	2.9E-05*	No significant outliers
		Removed 1	93	0.606 (0.482, 0.762)	4.3E-05*	No significant outliers
		Removed 2	91	0.604 (0.477, 0.765)	6.81E-05*	No significant outliers
CAD	Body lean mass	All	39	0.997 (0.986, 1.008)	0.575	rs10840293, rs11065979, rs11191416, rs17087335, rs2681472, rs3918226, rs56289821, rs663129, rs7528419
		Removed 1	36	0.995 (0.99, 1)	0.381	rs10840293, rs11065979, rs11191416, rs17087335, rs2681472, rs3918226, rs56289821, rs663129, rs7528419
CAD	Left handgrip strength	All	39	0.992 (0.983, 1)	0.052	rs2519093, rs663129, rs9349379
		Removed 1	36	0.991 (0.987, 0.995)	0.049	rs2519093, rs663129, rs9349379
CAD	Right handgrip strength	All	39	0.992 (0.983, 1)	0.064	rs2519093, rs663129, rs9349379
		Removed 1	36	0.991 (0.987, 0.995)	0.058	rs2519093, rs663129, rs9349379

MR-PRESSO analysis was only carried out for associations with a significant global test *p* value (*p* < 0.05).

^a^‘All’ represents analyses with all selected IVs; ‘Removed 1’ represents analyses after removing proxy IVs; ‘Removed 2’ represents analyses after removing potential pleiotropic SNPs (related to body fat mass and height).

Bonferroni corrected significance level (0.05/3 = 0.016) was used to correct for multiple comparisons.

*p<0.016.

CAD: coronary artery disease; MR-PRESSO: Mendelian Randomization Pleiotropy RESidual Sum and Outlier; IVs: instrumental variables; CI: confidence interval; OR: odds ratio.

After removing the proxy IVs and the potential pleiotropic IVs, both left and right handgrip strength were still negatively associated with CAD (Tables 1–3, Supplementary Table 1). However, our standard IVW and sensitivity analysis did not detect a significant association between genetically determined body lean mass and CAD (Tables 1–3).

### Stage 2: Influence of genetically predicted CAD on sarcopenia-related traits

In total, we obtained 39 LD-independent (*r*^2^ < 0.001) IVs that achieved genome-wide significance level (*p* < 5×10^−8^) from CAD (Supplementary Table 2). *F* statistics are presented to demonstrate the strength of the relationship between IVs and sarcopenia-related traits. The *F* statistics for selected IVs of CAD was larger than 10 (*F* = 64.17), and variance explained by these IVs is shown in Supplementary Table 3. Further, the negative control analysis results showed that CAD was not related to myopia, indicating the selected IVs of exposure were appropriate (Supplementary Tables 4–6).

As the heterogeneity test showed significant heterogeneity among the selected IVs (*p* < 0.05, Table 1), IVW in random-effects model was used. We did not detect a relationship between the genetically instrumented CAD and sarcopenia-related traits (body lean mass, left handgrip strength and right handgrip strength) in either IVW analysis or sensitivity analysis, even after removing the proxy IVs (Tables 1–3).

## DISCUSSION

In the current study, by applying bi-directional MR analysis, we successfully confirmed that increased handgrip strength was associated with decreased risk of CAD. However, no significant causal effect of CAD on handgrip strength was observed. Additionally, no significant causal relationship was detected between genetically determined body lean mass and CAD. To our knowledge, this is the first bi-directional MR study conducted on the topic of sarcopenia and CAD, simultaneously considering both muscle mass and muscle strength and the potential causality and reverse causality between sarcopenia and CAD.

The causal relationship of muscle strength with CAD has been discussed in previous TSMR studies [13, 14]. Xu and Hao’s TSMR analysis applied two genetic variants that predict handgrip strength in a European population to CAD in a mixed population of various ethnicities, indicating that increased handgrip reduced the risk of CAD [13]. However, another MR analysis did not find evidence for causality in the associations between handgrip strength (European population) and CAD (mixed population). A total of 20 genetic variants were selected to predict handgrip strength in this study [14]. These inconsistent results may be caused by different ancestry of exposure (handgrip strength) and outcome (CAD), the number of genetic variants selected as IVs, different methods applied, and even potential pleiotropic confounding IVs (body fat mass-related genetics variables). Compared to these two previous TSMR analyses [13, 14], more selected IVs were utilized to increase the interpretation of exposures. Several sensitivity analyses were performed to reduce the potential pleiotropic IVs, and results indicated a decreased causal effect of handgrip strength on risk of CAD. Epidemiological evidence has also suggested that muscle strength is negatively associated with CVD events and mortality [15], and similar results were also obtained between handgrip strength and CVD in UK individuals with one-sample MR analysis [15, 16].

Furthermore, a serial of traditional studies have frequently examined the association between muscle mass and CVD; however, they reached conflicting conclusions [5, 6, 17]. Previous studies indicated that the conflicting association of muscle mass with CVD might be confounded by body fat mass [7, 8] and subtypes of CVD (CAD, stroke, heart failure and atrial fibrillation). Other studies found evidence for low muscle mass as an independent risk factor of CAD [5], while inverse results were observed in obese postmenopausal women [6]. A possible mechanism underlying these contrasting association results between muscle mass and CVD might be a confounding common risk factor of high body fat mass [7, 8]. Accumulated body fat mass induces chronic inflammation, which further contributes to the development and progression of sarcopenia and CAD [7–9]. Unfortunately, few previous studies have been conducted to determine the causation between muscle mass and CAD, especially in the context of robust MR analysis for causal inference. In this study, we used a bi-directional MR study to make up for the lack of causal relationship between muscle mass and CAD, suggesting there is no significant causal relationship between genetically determined muscle mass and CAD even after removing genetic variants associated with potential confounders (body fat mass, body fat percentage and body fat distribution).

The main strength of this study is the application of bidirectional MR design, which is more robust to confounding and reverse causation and could clarify the causal direction between sarcopenia-related traits (muscle mass and muscle strength) and CAD. Secondly, we included absolute handgrip strength, which might be more appropriate to assess muscle strength than relative handgrip strength (handgrip strength/body weight), as relative grip strength may not only represent a change in muscle strength but also a change in body fat mass and bone mass. Additionally, several sensitivity analyses were performed to ensure the valid estimation of true MR causal effect size. Furthermore, as sarcopenia and CAD are closely related to body fat mass, we also detected and removed the potential pleiotropic effects introduced by those SNPs associated with body fat mass-related traits (body fat mass, body fat distribution and body fat percentage) by obtaining their association effect size from the GWAS Catalog (https://www.ebi.ac.uk/gwas) [18]. Finally, both sarcopenia and CAD unrelated trait (myopia) was used as negative control to demonstrate the validity of selected IVs, further validating our analyses and results.

This study has some potential limitations. Firstly, we used body lean mass rather than appendicular lean mass to measure muscle mass, which might be not exactly appropriate, as the measurement could be biased by other components of non-fat soft tissue, such as lungs, liver, and other viscera. Moreover, compared with absolute muscle mass, relative muscle mass (appendicular lean mass/height^2^) may be more appropriate to measure muscle mass [1]. Therefore, the conclusion of no significant association between muscle mass and CAD might not be easily generalized. Secondly, it is important to acknowledge that MR analysis has several other potential limitations [19], and while using multiple genetic instruments improves the power of MR, there is always some risk of pleiotropy despite extensive sensitivity analyses. Therefore, Mendelian Randomization Pleiotropy RESidual Sum and Outlier (MR-PRESSO) was performed to identify and remove the possible pleiotropic genetic variables and provide outlier-adjusted estimates, which should have minimized this possibility. Thirdly, we believe that sex- and age-specific analysis would be helpful, given the difference in incidence of sarcopenia and CAD by age and sex. As we only used summary statistics and had no access to the original individual measures, sex- and age-specific analysis may be difficult to perform. Fortunately, age and sex were all adjusted in genetics association of CARDIoGRAMplusCD4 and UK Biobank consortium [20–22], which should have minimized this possibility. Finally, as we only used summary statistics and had no access to the original individual measures, different standards of quality control and selection in individual-level GWAS may affect our results. Therefore, the results may not be easily generalized.

In summary, we found that increased genetically predicted muscle strength was causally associated with decreased risk of CAD, yet we did not identify a significant causal effect of CAD on muscle strength. Moreover, no significant causal association between muscle mass and CAD was observed in bi-directional MR analysis. Our results suggest that decreased muscle strength, but not decreased muscle mass, leads to the increased risk of CAD in sarcopenia.

## MATERIALS AND METHODS

### Study design

**Figure 1:**
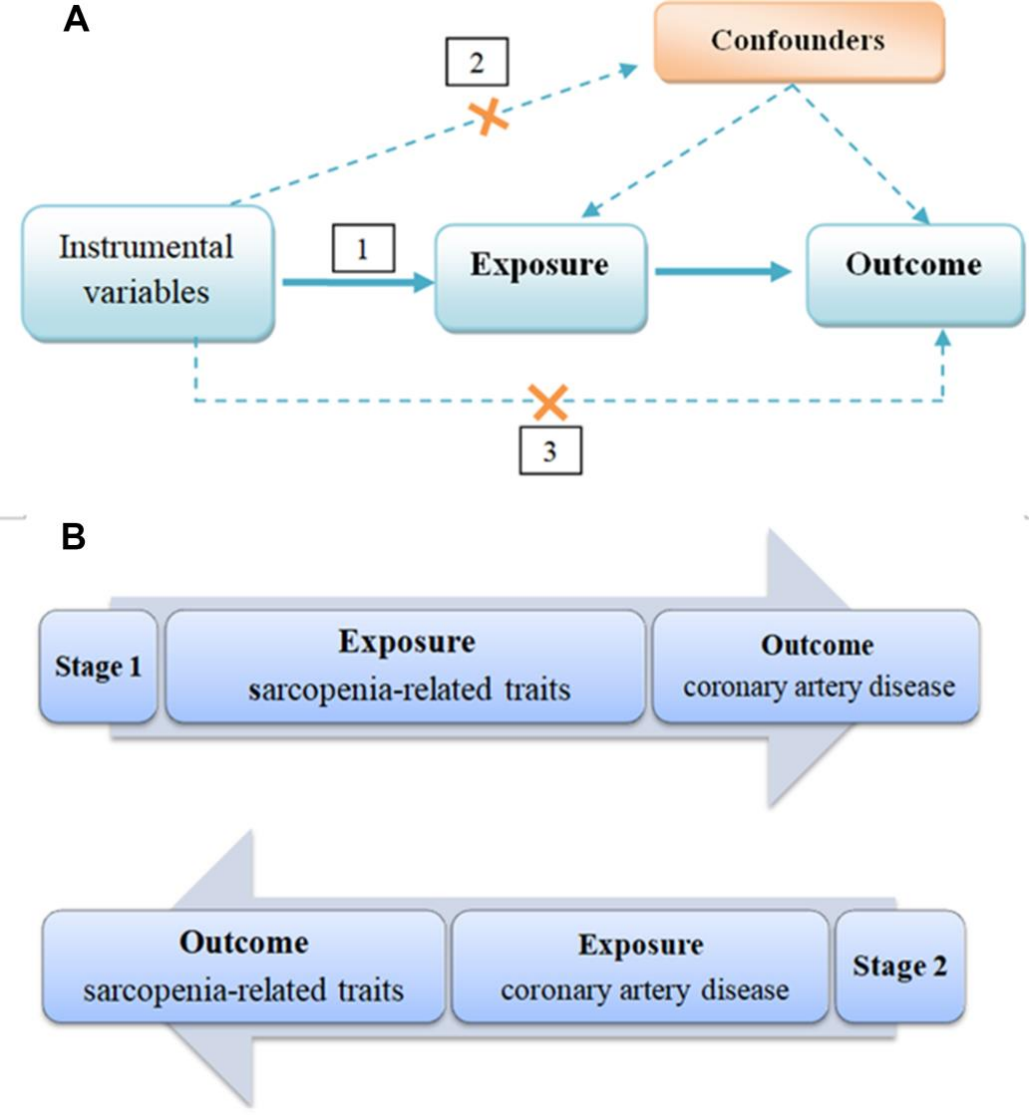
**Schematic Representation of Bi-directional MR Analysis.** (**A**) illustrates three assumptions of MR analysis as follows: 1) Instrumental variables must be associated with exposure, 2) instrumental variables must not be associated with confounders, and 3) instrumental variables must influence outcome only through exposure. (**B**) illustrates bi-directional MR analysis. In stage 1 analysis, influence of genetically predicted sarcopenia-related traits (body lean mass, left handgrip strength and right handgrip strength) on risk of CAD was estimated. In stage 2 analysis, influence of genetically predicted CAD on risk of sarcopenia-related traits (body lean mass, left handgrip strength and right handgrip strength) was estimated. TSMR: two-sample mendelian randomization; CAD: coronary artery disease.

The genetic variants used as IVs in TSMR analysis must satisfy three assumptions as follows (Figure 1A): 1) IVs are strongly associated with exposure. 2) The IVs are independent of any known confounders. 3) The selected IVs are conditionally independent of outcome, given exposure and potential confounders. The second and third assumptions are known as independence from pleiotropy [23]. In the current study, we performed a bi-directional MR study design to clarify the causal association between sarcopenia-related traits (muscle mass and muscle strength) and CAD with independent GWAS summary-level dataset (Figure 1B). In the first stage, we assessed whether sarcopenia-related traits were causally related to CAD. In the second stage, we also examined whether genetically driven CAD was causally associated with sarcopenia-related traits.

### Sarcopenia-related traits

Bioelectrical impedance analysis (BIA) was used to measure body lean mass (kilogram, kg) as a valid alternative for the assessment of body composition [24]. Body lean mass represents lipid-free soft tissue including muscle mass, body water, protein, glycerol and soft tissue mineral mass [25], which is considered to be a valid measure of muscle mass [26].

Handgrip strength (kg) was measured by a calibrated hydraulic hand dynamometer adjusted for hand size [20]. We used absolute rather than relative handgrip strength (absolute handgrip strength/weight) as a proxy for muscle strength, because absolute handgrip strength might have a higher correlation with muscle strength than relative grip strength [27].

### Data sources

GWAS summary statistics for body lean mass (n = 331, 291) and handgrip strength (left hand, n = 335, 821; right hand, n = 335, 842) were obtained from the UK biobank study [20], and CAD summary statistics (n = 184, 305) were obtained from the CARDIoGRAMplusC4D consortium [21]. Sarcopenia-related traits’ GWAS summary statistics of UK Biobank were conducted on European populations from population-based cohorts, and age, age^2^, sex, sex × age and sex × age^2^ were adjusted for in genetic association analysis [20]. CARDIoGRAMplusC4D Genomes-based GWAS summary statistics is a meta-analysis of 48 GWAS studies involving 60,801 CAD cases and 123,504 controls from European (~ 74%) and Asian (~ 26%) subjects, adjusted for sex and age in analyses of individual GWASs [21, 22].

### Selection and validation of IVs

To satisfy the three assumptions of MR (Figure 1A), we firstly selected independent (linkage disequilibrium *r*^2^ < 0.001) [28] SNPs that were strongly (*p* < 5 × 10^−8^) associated with exposure. Then, we obtained the corresponding effect estimates of these SNPs in outcome GWAS summary statistics. For the SNPs that were not available in the outcome, we used proxy SNPs that were highly correlated (*r*^2^ > 0.8) with the requested SNPs.

Additionally, MR-Egger regression was applied to assess the horizontal pleiotropy of selected IVs [23], and the intercept that deviates from the origin may provide evidence for potential pleiotropic effects across the IVs. We also performed MR-PRESSO to identify and remove pleiotropic IVs [29]. To further validate the strength of the selected IVs, we computed the *F* statistic of selected IVs using an online tool (https://sb452.shinyapps.io/overlap) [30]. *F* statistics greater than 10 are often considered as powerful enough to mitigate any potential bias of the causal IV estimate. Finally, MR-Steiger test was conducted to calculate the pooled variance explained in exposure and outcome by the selected IVs (*r*^2^) [31], and to further assess whether the variance explained in the exposure is larger than that in the outcome.

### MR analysis

IVW was conducted to estimate the causal effect between exposure and outcome, which was calculated as the SNP-outcome association effect size divided by the SNP-exposure association effect size [32]. The causal effect *β* was estimated as *w_i_* (*α_i_*/*γ_i_*), where *i* refers to the *i*th IV, *α_i_* defines as the association effect of IVs on exposure, *γ_i_* represents the association effect of IVs on outcome, and *w_i_* means the weights of the causal effect of exposure on outcome [32]. The IVW method was considered the most reliable indicator if there was no evidence of directional pleiotropy (*p* for MR-Egger intercept > 0.05) among the selected IVs [33, 34]. Given the multiple testing situation (body lean mass, left handgrip strength and right handgrip strength were included), we used a conservative approach and applied a Bonferroni corrected significance level of 0.016 (0.05/3).

### Sensitivity analysis

To further ensure the valid estimation of the true MR causal effect, we conducted several sensitivity analyses. Firstly, weighted median estimate was performed because it tends to provide valid estimates when at least 50% of information is derived from valid IVs [35]. Secondly, the MR might fail if the selected IVs are weak instruments. Therefore, we carried out a recently proposed method called Robust Adjusted Profile Score (RAPS), which considers the measurement error in SNP-exposure effects and is unbiased even when there are many (e.g. hundreds of) weak instruments [36]. Also, RAPS is robust to systematic pleiotropy. Thirdly, MR-PRESSO was performed to identify and remove the possible pleiotropic IVs, assuming that at least 50% of the IVs were valid IVs [29]. MR-PRESSO can also identify outlier IVs and provides outlier-adjusted estimates. MR-PRESSO detects pleiotropy by assessing outliers among the selected IVs contributing to the MR estimate and provides adjusted estimates. MR-PRESSO analysis was only carried out for associations with a significant global test *p* value *(p* < 0.05). Next, in order to guarantee that the MR estimates were not influenced by the inclusion of proxy SNPs, we repeated the analysis after excluding proxy SNPs. Finally, as muscle mass and CAD were closely related to body fat mass, we intend to exclude the potential pleiotropic effects introduced by those SNPs associated with body fat-related traits (body fat distribution and body fat percentage) by obtaining their association effect size from GWAS Catalog (https://www.ebi.ac.uk/gwas) [18].

### Negative control

To further demonstrate the validity of the selected IVs, we included myopia as negative control in our analysis, as there is little evidence presented that sarcopenia and CAD are associated with myopia. The summary statistics of myopia were derived from UK Biobank imputed genotype data, including 335,700 individuals of European descent [20].

All the analyses were performed using R statistical software (Version 3.4.2) with the R packages “*TwosampleMR*”, “*MendelianRandomization*” and “*MRPRESSO*”.

## Supplementary Material

Supplementary Tables

Supplementary Table 1

Supplementary Table 2

Supplementary Table 4
