# Supplementary material for: Sarcopenia-related traits and coronary artery disease: a bi-directional Mendelian randomization study
Source: Aging (Albany NY). 2020 Feb 16;12(4):3340–53. doi: 10.18632/aging.102815 (PMC7066916; doi:10.18632/aging.102815)
Supplement: Supplementary Tables [file aging-12-102815-s004..pdf]

SUPPLEMENTARY TABLES

Please browse Full Text version to see the data of Supplementary Tables 1, 2, 4

Supplementary Table 1. Independent IVs of body lean mass (kg), handgrip strength (left, kg) and handgrip strength (right, kg) in stage 1 analysis.

Supplementary Table 2. Independent IVs of body lean mass (kg), handgrip strength (left, kg) and handgrip strength (right, kg) in stage 2 analysis.

Supplementary Table 3. MR-Egeer analysis and variance explained by IVs in stage 2 analysis.

Exposure	outcome	egger_intercept	se	p-value	snp_r2.exposure	snp_r2.outcome	MR-Steiger test
**CAD**	Body lean mass	0.003232	0.003535	0.3664673	0.0134791	0.0028114	TRUE
**CAD**	Hand grip strength (left)	-0.00016	0.0014287	0.9125408	0.0134791	0.0003529	TRUE
**CAD**	Hand grip strength (right)	-0.00054	0.0013761	0.6950098	0.0134791	0.0003309	TRUE

Supplementary Table 4. Independent IVs of body lean mass (kg), handgrip strength (left, kg) and handgrip strength (right, kg) and coronary artery disease in negative control analysis.

Supplementary Table 5. Two-sample mendelian randomization analysis of negative control (myopia).

Exposure	outcome	method	n. IVs	P.value	beta (95% CI)
**CAD**	myopia	MR Egger	39	0.374	-0.004 (-0.013,0.005)
**CAD**	myopia	Weighted median	39	0.238	-0.003(-0.007,0.002)
**CAD**	myopia	Inverse variance weighted	39	0.75	0.001(-0.003,0.004)
**CAD**	myopia	Inverse variance weighted (multiplicative random effects)	39	0.75	0.001(-0.003,0.004)
**CAD**	myopia	Inverse variance weighted (fixed effects)	39	0.657	0.001(-0.002,0.003)
**CAD**	myopia	Robust adjusted profile score (RAPS)	39	0.99	0(-0.004,0.004)
**body lean mass**	myopia	MR Egger	405	0.883	-0.001(-0.018,0.015)
**body lean mass**	myopia	Weighted median	405	0.02	0.009(0.001,0.017)
**body lean mass**	myopia	Inverse variance weighted	405	0.17	0.005(-0.002,0.011)
**body lean mass**	myopia	Inverse variance weighted (multiplicative random effects)	405	0.17	0.005(-0.002,0.011)
**body lean mass**	myopia	Inverse variance weighted (fixed effects)	405	0.065	0.005(0,0.01)
**body lean mass**	myopia	Robust adjusted profile score (RAPS)	405	0.099	0.006(-0.001,0.012)
**Hand grip strength (left)**	myopia	MR Egger	85	0.233	0.039(-0.024,0.102)
**Hand grip strength (left)**	myopia	Weighted median	85	0.145	0.014(-0.005,0.032)
**Hand grip strength (left)**	myopia	Inverse variance weighted	85	0.399	0.007(-0.009,0.022)
**Hand grip strength (left)**	myopia	Inverse variance weighted (multiplicative random effects)	85	0.399	0.007(-0.009,0.022)
**Hand grip strength (left)**	myopia	Inverse variance weighted (fixed effects)	85	0.264	0.007(-0.005,0.019)
**Hand grip strength (left)**	myopia	Robust adjusted profile score (RAPS)	85	0.262	0.009(-0.007,0.024)
**Hand grip strength (right)**	myopia	MR Egger	101	0.173	0.035(-0.015,0.085)
**Hand grip strength (right)**	myopia	Weighted median	101	0.176	0.011(-0.005,0.027)
**Hand grip strength (right)**	myopia	Inverse variance weighted	101	0.505	0.004(-0.009,0.018)
**Hand grip strength (right)**	myopia	Inverse variance weighted (multiplicative random effects)	101	0.505	0.004(-0.009,0.018)
**Hand grip strength (right)**	myopia	Inverse variance weighted (fixed effects)	101	0.421	0.004(-0.006,0.015)
**Hand grip strength (right)**	myopia	Robust adjusted profile score (RAPS)	101	0.457	0.005(-0.008,0.018)

CAD: coronary artery disease; IVs: instrumental variables.

Supplementary Table 6. MR-Egeer analysis and explained by selected IVs of negative control (myopia).

Exposure	outcome	MR-Egeer. intercept	se	p.val	r^2.exposure	r^2.outcome
**CAD**	myopia	0.0004999	0.0004338	0.25656	0.0134791	0.0002211
**lean mass**	myopia	0.0000988	0.0001305	0.44943	0.0898249	0.0022361
**Hand grip strength (left)**	myopia	-0.0004489	0.0004375	0.307857	0.0119751	0.0004522
**Hand grip strength (right)**	myopia	-0.0004309	0.0003468	0.216888	0.0142341	0.0004624

r^2 means phenotypes explained by selected IVs. CAD: coronary artery disease; IVs: instrumental variables.
