# Supplementary material for: Sarcopenia-related traits and coronary artery disease: a bi-directional Mendelian randomization study
Source: Aging (Albany NY). 2020 Feb 16;12(4):3340–53. doi: 10.18632/aging.102815 (PMC7066916; doi:10.18632/aging.102815)
Supplement: Supplementary Table 1 [file aging-12-102815-s003..docx]

**Supplementary Table 1. Independent IVs of body lean mass (kg), handgrip strength (left, kg) and handgrip strength (right, kg) in stage 1 analysis.**

| SNP | exposure | outcome | beta.exposure | beta.outcome | se.exposure | se.outcome | pval.exposure | pval.outcome | proxy.outcome | target_snp.outcome | proxy_snp.outcome | potential confounders |
| --- | --- | --- | --- | --- | --- | --- | --- | --- | --- | --- | --- | --- |
| rs10203320 | Body lean mass | CAD | 0.012 | 0.014 | 0.002 | 0.011 | 3.08E-13 | 0.207 | NA | NA | NA | NA |
| rs1022523 | Body lean mass | CAD | 0.014 | -0.004 | 0.002 | 0.010 | 1.44E-16 | 0.705 | NA | NA | NA | NA |
| rs10236214 | Body lean mass | CAD | 0.020 | -0.018 | 0.002 | 0.011 | 1.12E-36 | 0.089 | NA | NA | NA | NA |
| rs10237306 | Body lean mass | CAD | 0.011 | -0.005 | 0.002 | 0.010 | 1.01E-12 | 0.631 | NA | NA | NA | NA |
| rs10257870 | Body lean mass | CAD | 0.016 | 0.016 | 0.003 | 0.016 | 4.59E-08 | 0.292 | NA | NA | NA | NA |
| rs10260993 | Body lean mass | CAD | -0.012 | 0.017 | 0.002 | 0.011 | 3.39E-10 | 0.125 | NA | NA | NA | NA |
| rs1043413 | Body lean mass | CAD | 0.010 | 0.029 | 0.002 | 0.009 | 9.60E-10 | 0.002 | NA | NA | NA | NA |
| rs1047891 | Body lean mass | CAD | 0.017 | -0.025 | 0.002 | 0.011 | 1.51E-25 | 0.018 | NA | NA | NA | NA |
| rs10483727 | Body lean mass | CAD | -0.022 | 0.017 | 0.002 | 0.010 | 1.46E-43 | 0.073 | NA | NA | NA | NA |
| rs10485622 | Body lean mass | CAD | -0.019 | -0.005 | 0.002 | 0.012 | 9.84E-22 | 0.664 | NA | NA | NA | NA |
| rs1064213 | Body lean mass | CAD | 0.012 | 0.005 | 0.002 | 0.010 | 5.88E-14 | 0.588 | NA | NA | NA | NA |
| rs10746837 | Body lean mass | CAD | -0.010 | 0.020 | 0.002 | 0.009 | 3.06E-11 | 0.037 | NA | NA | NA | NA |
| rs10748128 | Body lean mass | CAD | 0.012 | 0.004 | 0.002 | 0.010 | 3.47E-13 | 0.663 | NA | NA | NA | NA |
| rs10770705 | Body lean mass | CAD | -0.009 | 0.011 | 0.002 | 0.010 | 9.40E-09 | 0.269 | NA | NA | NA | NA |
| rs10775348 | Body lean mass | CAD | 0.014 | -0.002 | 0.002 | 0.012 | 4.41E-16 | 0.893 | NA | NA | NA | NA |
| rs10798945 | Body lean mass | CAD | 0.012 | 0.003 | 0.002 | 0.011 | 7.47E-11 | 0.817 | NA | NA | NA | NA |
| rs10843139 | Body lean mass | CAD | -0.013 | -0.006 | 0.002 | 0.011 | 4.07E-15 | 0.560 | NA | NA | NA | NA |
| rs10847415 | Body lean mass | CAD | 0.015 | 0.004 | 0.002 | 0.011 | 9.38E-19 | 0.717 | NA | NA | NA | NA |
| rs10931008 | Body lean mass | CAD | -0.013 | 0.020 | 0.002 | 0.010 | 5.11E-15 | 0.048 | NA | NA | NA | NA |
| rs10938398 | Body lean mass | CAD | 0.011 | 0.031 | 0.002 | 0.009 | 5.98E-13 | 0.001 | NA | NA | NA | NA |
| rs10953112 | Body lean mass | CAD | 0.015 | 0.021 | 0.002 | 0.016 | 2.17E-14 | 0.192 | NA | NA | NA | NA |
| rs10958683 | Body lean mass | CAD | -0.010 | 0.011 | 0.002 | 0.012 | 3.05E-08 | 0.344 | NA | NA | NA | NA |
| rs10982888 | Body lean mass | CAD | -0.016 | -0.002 | 0.002 | 0.013 | 5.40E-11 | 0.900 | NA | NA | NA | NA |
| rs10990621 | Body lean mass | CAD | 0.018 | -0.011 | 0.002 | 0.014 | 9.51E-18 | 0.412 | NA | NA | NA | NA |
| rs10995366 | Body lean mass | CAD | -0.011 | -0.004 | 0.002 | 0.011 | 2.73E-10 | 0.687 | NA | NA | NA | NA |
| rs11013045 | Body lean mass | CAD | -0.009 | 0.014 | 0.002 | 0.010 | 6.27E-09 | 0.140 | NA | NA | NA | NA |
| rs11014285 | Body lean mass | CAD | 0.018 | -0.025 | 0.002 | 0.014 | 9.35E-18 | 0.081 | NA | NA | NA | NA |
| rs11023199 | Body lean mass | CAD | -0.009 | -0.009 | 0.002 | 0.010 | 1.27E-08 | 0.348 | NA | NA | NA | NA |
| rs11030119 | Body lean mass | CAD | 0.017 | 0.022 | 0.002 | 0.010 | 1.04E-23 | 0.030 | NA | NA | NA | NA |
| rs11065015 | Body lean mass | CAD | -0.031 | -0.036 | 0.005 | 0.034 | 6.97E-11 | 0.293 | NA | NA | NA | NA |
| rs11065979 | Body lean mass | CAD | -0.015 | 0.069 | 0.002 | 0.011 | 6.59E-22 | 0.000 | NA | NA | NA | NA |
| rs11098675 | Body lean mass | CAD | -0.016 | -0.009 | 0.002 | 0.011 | 6.61E-16 | 0.435 | NA | NA | NA | NA |
| rs1111818 | Body lean mass | CAD | 0.009 | 0.004 | 0.002 | 0.009 | 3.44E-09 | 0.645 | NA | NA | NA | NA |
| rs11125074 | Body lean mass | CAD | 0.009 | 0.001 | 0.002 | 0.009 | 2.69E-08 | 0.904 | NA | NA | NA | NA |
| rs11158820 | Body lean mass | CAD | -0.010 | -0.006 | 0.002 | 0.011 | 2.45E-09 | 0.573 | NA | NA | NA | NA |
| rs111632154 | Body lean mass | CAD | -0.030 | 0.016 | 0.004 | 0.033 | 4.73E-17 | 0.631 | NA | NA | NA | NA |
| rs111640872 | Body lean mass | CAD | 0.015 | -0.023 | 0.002 | 0.010 | 3.20E-20 | 0.030 | NA | NA | NA | NA |
| rs111901479 | Body lean mass | CAD | 0.024 | -0.046 | 0.003 | 0.017 | 3.67E-19 | 0.007 | NA | NA | NA | NA |
| rs111964059 | Body lean mass | CAD | 0.016 | -0.017 | 0.003 | 0.019 | 1.05E-08 | 0.354 | NA | NA | NA | NA |
| rs11205617 | Body lean mass | CAD | 0.011 | 0.012 | 0.002 | 0.010 | 4.82E-11 | 0.201 | NA | NA | NA | NA |
| rs11217863 | Body lean mass | CAD | -0.016 | 0.013 | 0.002 | 0.014 | 6.36E-11 | 0.370 | NA | NA | NA | NA |
| rs11240565 | Body lean mass | CAD | 0.014 | 0.011 | 0.002 | 0.009 | 3.09E-19 | 0.237 | NA | NA | NA | NA |
| rs11245450 | Body lean mass | CAD | -0.010 | -0.001 | 0.002 | 0.010 | 1.20E-10 | 0.928 | NA | NA | NA | NA |
| rs112957890 | Body lean mass | CAD | 0.012 | -0.003 | 0.002 | 0.012 | 1.24E-11 | 0.787 | NA | NA | NA | NA |
| rs113457986 | Body lean mass | CAD | 0.040 | 0.043 | 0.006 | 0.043 | 7.63E-10 | 0.314 | NA | NA | NA | NA |
| rs113902512 | Body lean mass | CAD | 0.011 | -0.008 | 0.002 | 0.019 | 4.80E-08 | 0.662 | NA | NA | NA | NA |
| rs11545482 | Body lean mass | CAD | -0.035 | -0.076 | 0.005 | 0.047 | 8.76E-11 | 0.107 | NA | NA | NA | NA |
| rs115574684 | Body lean mass | CAD | 0.022 | -0.001 | 0.004 | 0.026 | 2.84E-08 | 0.969 | NA | NA | NA | NA |
| rs11580196 | Body lean mass | CAD | -0.012 | -0.010 | 0.002 | 0.014 | 2.03E-09 | 0.481 | NA | NA | NA | NA |
| rs115912456 | Body lean mass | CAD | 0.025 | 0.014 | 0.004 | 0.025 | 9.03E-11 | 0.570 | NA | NA | NA | Body fat distribution |
| rs11594322 | Body lean mass | CAD | -0.017 | -0.003 | 0.002 | 0.014 | 1.23E-13 | 0.815 | NA | NA | NA | NA |
| rs116072427 | Body lean mass | CAD | -0.025 | -0.019 | 0.003 | 0.020 | 9.68E-17 | 0.357 | NA | NA | NA | NA |
| rs116165844 | Body lean mass | CAD | -0.014 | 0.018 | 0.002 | 0.015 | 8.10E-10 | 0.235 | NA | NA | NA | NA |
| rs11655578 | Body lean mass | CAD | 0.010 | 0.035 | 0.002 | 0.014 | 4.36E-08 | 0.011 | NA | NA | NA | NA |
| rs11657325 | Body lean mass | CAD | -0.016 | 0.010 | 0.002 | 0.011 | 4.02E-20 | 0.374 | NA | NA | NA | NA |
| rs11658134 | Body lean mass | CAD | -0.011 | -0.008 | 0.002 | 0.009 | 6.71E-13 | 0.370 | NA | NA | NA | NA |
| rs11667280 | Body lean mass | CAD | -0.011 | 0.016 | 0.002 | 0.012 | 4.69E-09 | 0.170 | NA | NA | NA | NA |
| rs11678716 | Body lean mass | CAD | -0.022 | 0.029 | 0.003 | 0.020 | 1.58E-12 | 0.146 | NA | NA | NA | NA |
| rs11689727 | Body lean mass | CAD | -0.011 | 0.011 | 0.002 | 0.010 | 4.83E-12 | 0.285 | NA | NA | NA | NA |
| rs11717749 | Body lean mass | CAD | 0.016 | 0.008 | 0.002 | 0.016 | 2.20E-11 | 0.647 | NA | NA | NA | NA |
| rs11783086 | Body lean mass | CAD | 0.009 | -0.007 | 0.002 | 0.010 | 1.69E-08 | 0.500 | NA | NA | NA | NA |
| rs11803905 | Body lean mass | CAD | 0.010 | -0.015 | 0.002 | 0.010 | 8.66E-09 | 0.142 | NA | NA | NA | NA |
| rs11855017 | Body lean mass | CAD | 0.013 | -0.009 | 0.002 | 0.012 | 8.34E-11 | 0.492 | NA | NA | NA | NA |
| rs11880992 | Body lean mass | CAD | 0.015 | -0.012 | 0.002 | 0.010 | 5.83E-21 | 0.238 | NA | NA | NA | NA |
| rs11951673 | Body lean mass | CAD | -0.009 | -0.018 | 0.002 | 0.010 | 2.86E-08 | 0.060 | NA | NA | NA | NA |
| rs12041740 | Body lean mass | CAD | -0.015 | 0.000 | 0.002 | 0.010 | 3.93E-18 | 0.965 | NA | NA | NA | NA |
| rs12072845 | Body lean mass | CAD | -0.015 | 0.008 | 0.002 | 0.009 | 2.70E-20 | 0.396 | NA | NA | NA | NA |
| rs12073468 | Body lean mass | CAD | 0.012 | 0.004 | 0.002 | 0.011 | 2.55E-10 | 0.701 | NA | NA | NA | NA |
| rs12140153 | Body lean mass | CAD | -0.016 | -0.032 | 0.003 | 0.021 | 1.83E-09 | 0.127 | NA | NA | NA | Body fat percentage |
| rs12193797 | Body lean mass | CAD | -0.021 | -0.015 | 0.002 | 0.016 | 3.45E-21 | 0.364 | NA | NA | NA | NA |
| rs12209223 | Body lean mass | CAD | 0.018 | 0.024 | 0.003 | 0.016 | 4.92E-12 | 0.133 | NA | NA | NA | NA |
| rs12216497 | Body lean mass | CAD | -0.011 | 0.014 | 0.002 | 0.009 | 4.31E-12 | 0.133 | NA | NA | NA | NA |
| rs1222219 | Body lean mass | CAD | -0.011 | 0.007 | 0.002 | 0.012 | 1.89E-09 | 0.542 | NA | NA | NA | NA |
| rs1228024 | Body lean mass | CAD | -0.011 | -0.014 | 0.002 | 0.010 | 2.66E-12 | 0.176 | NA | NA | NA | NA |
| rs12347137 | Body lean mass | CAD | -0.025 | 0.002 | 0.002 | 0.013 | 2.79E-39 | 0.887 | NA | NA | NA | NA |
| rs12452505 | Body lean mass | CAD | -0.019 | -0.001 | 0.002 | 0.015 | 1.68E-17 | 0.932 | NA | NA | NA | NA |
| rs12540011 | Body lean mass | CAD | 0.014 | -0.008 | 0.002 | 0.011 | 8.87E-16 | 0.449 | NA | NA | NA | NA |
| rs1260326 | Body lean mass | CAD | 0.021 | 0.003 | 0.002 | 0.010 | 4.40E-41 | 0.735 | NA | NA | NA | NA |
| rs12619348 | Body lean mass | CAD | -0.013 | 0.004 | 0.002 | 0.013 | 5.97E-09 | 0.747 | NA | NA | NA | NA |
| rs12682601 | Body lean mass | CAD | -0.011 | 0.010 | 0.002 | 0.010 | 4.68E-11 | 0.298 | NA | NA | NA | NA |
| rs12713004 | Body lean mass | CAD | 0.018 | -0.032 | 0.002 | 0.011 | 6.58E-27 | 0.004 | NA | NA | NA | NA |
| rs12813149 | Body lean mass | CAD | -0.010 | 0.006 | 0.002 | 0.011 | 4.89E-08 | 0.602 | NA | NA | NA | NA |
| rs1285990 | Body lean mass | CAD | 0.011 | -0.025 | 0.002 | 0.011 | 3.56E-11 | 0.022 | NA | NA | NA | NA |
| rs12879423 | Body lean mass | CAD | 0.015 | 0.009 | 0.002 | 0.010 | 2.28E-19 | 0.353 | NA | NA | NA | NA |
| rs12889702 | Body lean mass | CAD | 0.009 | -0.020 | 0.002 | 0.010 | 1.15E-08 | 0.044 | NA | NA | NA | NA |
| rs12906197 | Body lean mass | CAD | -0.013 | 0.013 | 0.002 | 0.010 | 9.54E-16 | 0.207 | NA | NA | NA | NA |
| rs13124829 | Body lean mass | CAD | 0.009 | -0.013 | 0.002 | 0.010 | 2.69E-08 | 0.183 | NA | NA | NA | NA |
| rs13148166 | Body lean mass | CAD | -0.017 | -0.011 | 0.002 | 0.009 | 7.37E-28 | 0.231 | NA | NA | NA | NA |
| rs13247154 | Body lean mass | CAD | 0.012 | -0.009 | 0.002 | 0.010 | 3.87E-14 | 0.351 | NA | NA | NA | NA |
| rs13294021 | Body lean mass | CAD | -0.011 | 0.005 | 0.002 | 0.009 | 9.90E-13 | 0.563 | NA | NA | NA | NA |
| rs13430869 | Body lean mass | CAD | 0.016 | -0.001 | 0.002 | 0.010 | 6.30E-19 | 0.913 | NA | NA | NA | NA |
| rs1351394 | Body lean mass | CAD | -0.027 | 0.020 | 0.002 | 0.010 | 3.38E-70 | 0.034 | NA | NA | NA | NA |
| rs139590892 | Body lean mass | CAD | -0.012 | -0.015 | 0.002 | 0.014 | 6.93E-09 | 0.270 | NA | NA | NA | NA |
| rs1407031 | Body lean mass | CAD | -0.010 | 0.012 | 0.002 | 0.009 | 1.45E-09 | 0.206 | NA | NA | NA | NA |
| rs141403611 | Body lean mass | CAD | -0.015 | 0.001 | 0.003 | 0.018 | 2.38E-08 | 0.948 | NA | NA | NA | NA |
| rs1431663 | Body lean mass | CAD | -0.009 | 0.009 | 0.002 | 0.009 | 4.39E-08 | 0.348 | NA | NA | NA | NA |
| rs143384 | Body lean mass | CAD | 0.038 | -0.015 | 0.002 | 0.010 | 6.02E-130 | 0.120 | NA | NA | NA | Body fat distribution |
| rs143986132 | Body lean mass | CAD | -0.035 | -0.038 | 0.006 | 0.049 | 3.14E-09 | 0.440 | NA | NA | NA | NA |
| rs1452822 | Body lean mass | CAD | 0.021 | -0.028 | 0.002 | 0.010 | 3.17E-35 | 0.006 | NA | NA | NA | NA |
| rs146851424 | Body lean mass | CAD | 0.065 | 0.024 | 0.005 | 0.035 | 3.84E-35 | 0.490 | NA | NA | NA | NA |
| rs147110934 | Body lean mass | CAD | -0.033 | 0.007 | 0.005 | 0.037 | 7.52E-11 | 0.858 | NA | NA | NA | NA |
| rs1472852 | Body lean mass | CAD | -0.032 | 0.020 | 0.002 | 0.012 | 5.37E-53 | 0.109 | NA | NA | NA | NA |
| rs1478575 | Body lean mass | CAD | 0.020 | 0.011 | 0.002 | 0.010 | 2.20E-33 | 0.249 | NA | NA | NA | NA |
| rs1521624 | Body lean mass | CAD | -0.009 | 0.002 | 0.002 | 0.009 | 3.00E-09 | 0.796 | NA | NA | NA | NA |
| rs153560 | Body lean mass | CAD | 0.011 | -0.011 | 0.002 | 0.010 | 9.08E-12 | 0.282 | NA | NA | NA | NA |
| rs1542224 | Body lean mass | CAD | 0.015 | -0.019 | 0.002 | 0.011 | 2.00E-17 | 0.093 | NA | NA | NA | NA |
| rs1552234 | Body lean mass | CAD | 0.009 | 0.020 | 0.002 | 0.009 | 1.42E-08 | 0.029 | NA | NA | NA | NA |
| rs1573891 | Body lean mass | CAD | -0.023 | 0.024 | 0.002 | 0.013 | 3.68E-27 | 0.059 | NA | NA | NA | NA |
| rs1582931 | Body lean mass | CAD | -0.020 | -0.016 | 0.002 | 0.009 | 1.23E-39 | 0.089 | NA | NA | NA | NA |
| rs1591806 | Body lean mass | CAD | 0.017 | -0.031 | 0.002 | 0.010 | 6.80E-27 | 0.001 | NA | NA | NA | NA |
| rs161799 | Body lean mass | CAD | -0.009 | -0.004 | 0.002 | 0.010 | 3.78E-08 | 0.689 | NA | NA | NA | NA |
| rs16869017 | Body lean mass | CAD | 0.018 | 0.005 | 0.003 | 0.016 | 1.28E-10 | 0.739 | NA | NA | NA | NA |
| rs16892552 | Body lean mass | CAD | -0.015 | 0.009 | 0.002 | 0.011 | 2.59E-16 | 0.402 | NA | NA | NA | NA |
| rs16942324 | Body lean mass | CAD | -0.040 | 0.011 | 0.005 | 0.033 | 1.09E-16 | 0.735 | NA | NA | NA | NA |
| rs16964211 | Body lean mass | CAD | -0.021 | 0.003 | 0.004 | 0.015 | 7.70E-09 | 0.829 | NA | NA | NA | NA |
| rs17056859 | Body lean mass | CAD | 0.009 | 0.002 | 0.002 | 0.010 | 9.82E-09 | 0.817 | NA | NA | NA | NA |
| rs17157112 | Body lean mass | CAD | -0.009 | -0.007 | 0.002 | 0.010 | 2.47E-09 | 0.439 | NA | NA | NA | NA |
| rs1716162 | Body lean mass | CAD | -0.013 | 0.010 | 0.002 | 0.011 | 1.18E-11 | 0.361 | NA | NA | NA | NA |
| rs17197114 | Body lean mass | CAD | 0.013 | 0.037 | 0.002 | 0.014 | 1.52E-10 | 0.006 | NA | NA | NA | NA |
| rs17363646 | Body lean mass | CAD | 0.015 | -0.014 | 0.002 | 0.013 | 7.56E-11 | 0.282 | NA | NA | NA | NA |
| rs17454369 | Body lean mass | CAD | 0.022 | -0.015 | 0.003 | 0.024 | 3.08E-11 | 0.536 | NA | NA | NA | NA |
| rs17741497 | Body lean mass | CAD | -0.010 | 0.001 | 0.002 | 0.010 | 1.41E-09 | 0.933 | NA | NA | NA | NA |
| rs17770336 | Body lean mass | CAD | 0.011 | 0.024 | 0.002 | 0.010 | 3.71E-11 | 0.022 | NA | NA | NA | NA |
| rs1789164 | Body lean mass | CAD | 0.010 | -0.009 | 0.002 | 0.010 | 1.21E-10 | 0.384 | NA | NA | NA | NA |
| rs1805165 | Body lean mass | CAD | -0.010 | -0.007 | 0.002 | 0.010 | 1.74E-08 | 0.475 | NA | NA | NA | NA |
| rs1815518 | Body lean mass | CAD | 0.016 | -0.006 | 0.002 | 0.013 | 5.09E-15 | 0.649 | NA | NA | NA | NA |
| rs183041 | Body lean mass | CAD | 0.014 | 0.000 | 0.002 | 0.012 | 5.12E-16 | 0.996 | NA | NA | NA | NA |
| rs1837367 | Body lean mass | CAD | 0.009 | 0.013 | 0.002 | 0.010 | 3.42E-09 | 0.188 | NA | NA | NA | NA |
| rs1878528 | Body lean mass | CAD | 0.013 | 0.010 | 0.002 | 0.010 | 1.36E-14 | 0.339 | NA | NA | NA | NA |
| rs1881975 | Body lean mass | CAD | -0.014 | -0.011 | 0.002 | 0.012 | 4.31E-15 | 0.357 | NA | NA | NA | NA |
| rs1887855 | Body lean mass | CAD | -0.010 | 0.022 | 0.002 | 0.010 | 6.03E-09 | 0.031 | NA | NA | NA | NA |
| rs1910252 | Body lean mass | CAD | 0.016 | 0.002 | 0.002 | 0.012 | 6.90E-15 | 0.830 | NA | NA | NA | NA |
| rs1927635 | Body lean mass | CAD | 0.010 | -0.003 | 0.002 | 0.010 | 1.59E-09 | 0.763 | NA | NA | NA | NA |
| rs1952527 | Body lean mass | CAD | -0.012 | 0.024 | 0.002 | 0.010 | 8.84E-14 | 0.015 | NA | NA | NA | NA |
| rs197372 | Body lean mass | CAD | 0.010 | -0.021 | 0.002 | 0.010 | 1.34E-09 | 0.030 | NA | NA | NA | NA |
| rs2005172 | Body lean mass | CAD | 0.023 | -0.008 | 0.002 | 0.010 | 1.42E-47 | 0.431 | NA | NA | NA | NA |
| rs2073272 | Body lean mass | CAD | -0.010 | -0.016 | 0.002 | 0.010 | 2.81E-09 | 0.121 | NA | NA | NA | NA |
| rs2102278 | Body lean mass | CAD | 0.011 | 0.002 | 0.002 | 0.010 | 2.41E-11 | 0.876 | NA | NA | NA | NA |
| rs2118663 | Body lean mass | CAD | -0.011 | -0.016 | 0.002 | 0.012 | 1.88E-08 | 0.181 | NA | NA | NA | NA |
| rs2134963 | Body lean mass | CAD | -0.011 | -0.020 | 0.002 | 0.010 | 1.48E-10 | 0.037 | NA | NA | NA | NA |
| rs2140046 | Body lean mass | CAD | -0.013 | 0.004 | 0.002 | 0.010 | 6.97E-17 | 0.707 | NA | NA | NA | NA |
| rs2142331 | Body lean mass | CAD | -0.013 | -0.006 | 0.002 | 0.010 | 1.75E-17 | 0.513 | NA | NA | NA | NA |
| rs216193 | Body lean mass | CAD | -0.009 | -0.038 | 0.002 | 0.009 | 3.86E-09 | 0.000 | NA | NA | NA | NA |
| rs2197563 | Body lean mass | CAD | 0.011 | -0.030 | 0.002 | 0.010 | 3.72E-12 | 0.002 | NA | NA | NA | NA |
| rs2229840 | Body lean mass | CAD | 0.022 | 0.009 | 0.002 | 0.013 | 8.04E-25 | 0.482 | NA | NA | NA | NA |
| rs2230033 | Body lean mass | CAD | -0.013 | -0.004 | 0.002 | 0.009 | 2.80E-18 | 0.693 | NA | NA | NA | NA |
| rs2252720 | Body lean mass | CAD | -0.014 | -0.004 | 0.002 | 0.010 | 1.31E-17 | 0.688 | NA | NA | NA | NA |
| rs2270894 | Body lean mass | CAD | -0.017 | -0.018 | 0.002 | 0.014 | 5.75E-17 | 0.190 | NA | NA | NA | NA |
| rs2274116 | Body lean mass | CAD | -0.010 | 0.002 | 0.002 | 0.011 | 3.72E-09 | 0.837 | NA | NA | NA | NA |
| rs2277138 | Body lean mass | CAD | -0.018 | 0.016 | 0.002 | 0.009 | 3.66E-31 | 0.091 | NA | NA | NA | NA |
| rs2280940 | Body lean mass | CAD | 0.012 | -0.002 | 0.002 | 0.010 | 1.92E-12 | 0.870 | NA | NA | NA | NA |
| rs2281175 | Body lean mass | CAD | 0.011 | -0.007 | 0.002 | 0.014 | 8.41E-12 | 0.616 | NA | NA | NA | NA |
| rs2289976 | Body lean mass | CAD | 0.012 | 0.037 | 0.002 | 0.011 | 2.88E-12 | 0.001 | NA | NA | NA | NA |
| rs2291256 | Body lean mass | CAD | 0.016 | 0.019 | 0.003 | 0.018 | 1.06E-08 | 0.284 | NA | NA | NA | NA |
| rs2296316 | Body lean mass | CAD | -0.011 | 0.013 | 0.002 | 0.009 | 1.59E-12 | 0.182 | NA | NA | NA | NA |
| rs2305758 | Body lean mass | CAD | 0.010 | -0.008 | 0.002 | 0.011 | 3.70E-09 | 0.470 | NA | NA | NA | NA |
| rs2307111 | Body lean mass | CAD | -0.018 | -0.008 | 0.002 | 0.010 | 1.14E-30 | 0.429 | NA | NA | NA | NA |
| rs2395617 | Body lean mass | CAD | 0.019 | -0.015 | 0.002 | 0.016 | 4.47E-16 | 0.340 | NA | NA | NA | NA |
| rs2396348 | Body lean mass | CAD | 0.012 | 0.014 | 0.002 | 0.010 | 2.00E-12 | 0.176 | NA | NA | NA | NA |
| rs2411453 | Body lean mass | CAD | -0.012 | -0.017 | 0.002 | 0.011 | 1.74E-15 | 0.122 | NA | NA | NA | Body fat percentage |
| rs2455561 | Body lean mass | CAD | -0.012 | 0.011 | 0.002 | 0.009 | 1.12E-15 | 0.240 | NA | NA | NA | NA |
| rs2457982 | Body lean mass | CAD | 0.011 | 0.007 | 0.002 | 0.010 | 1.35E-09 | 0.518 | NA | NA | NA | NA |
| rs247008 | Body lean mass | CAD | 0.015 | 0.011 | 0.002 | 0.010 | 3.16E-20 | 0.260 | NA | NA | NA | NA |
| rs2476998 | Body lean mass | CAD | 0.010 | -0.011 | 0.002 | 0.010 | 2.03E-08 | 0.287 | NA | NA | NA | NA |
| rs2482357 | Body lean mass | CAD | -0.010 | -0.001 | 0.002 | 0.009 | 8.01E-10 | 0.945 | NA | NA | NA | NA |
| rs2490637 | Body lean mass | CAD | -0.019 | 0.009 | 0.003 | 0.017 | 6.46E-09 | 0.605 | NA | NA | NA | NA |
| rs2503756 | Body lean mass | CAD | 0.010 | -0.012 | 0.002 | 0.009 | 1.02E-10 | 0.189 | NA | NA | NA | NA |
| rs252758 | Body lean mass | CAD | -0.017 | 0.011 | 0.002 | 0.012 | 9.42E-22 | 0.324 | NA | NA | NA | NA |
| rs252937 | Body lean mass | CAD | -0.010 | 0.011 | 0.002 | 0.010 | 8.20E-09 | 0.274 | NA | NA | NA | NA |
| rs2533879 | Body lean mass | CAD | -0.022 | -0.004 | 0.002 | 0.011 | 6.70E-40 | 0.700 | NA | NA | NA | NA |
| rs2539999 | Body lean mass | CAD | 0.017 | 0.000 | 0.002 | 0.012 | 1.91E-23 | 0.983 | NA | NA | NA | NA |
| rs2540034 | Body lean mass | CAD | 0.015 | -0.007 | 0.002 | 0.010 | 9.35E-21 | 0.497 | NA | NA | NA | NA |
| rs254963 | Body lean mass | CAD | -0.010 | 0.005 | 0.002 | 0.010 | 4.19E-10 | 0.617 | NA | NA | NA | NA |
| rs2592831 | Body lean mass | CAD | 0.013 | -0.004 | 0.002 | 0.010 | 7.54E-16 | 0.673 | NA | NA | NA | NA |
| rs2602713 | Body lean mass | CAD | 0.010 | -0.016 | 0.002 | 0.010 | 3.58E-11 | 0.116 | NA | NA | NA | NA |
| rs2678204 | Body lean mass | CAD | 0.012 | 0.038 | 0.002 | 0.010 | 2.10E-14 | 0.000 | NA | NA | NA | NA |
| rs2733287 | Body lean mass | CAD | 0.009 | 0.021 | 0.002 | 0.009 | 1.83E-09 | 0.022 | NA | NA | NA | NA |
| rs2744965 | Body lean mass | CAD | 0.039 | 0.045 | 0.002 | 0.014 | 9.87E-70 | 0.001 | NA | NA | NA | NA |
| rs2803888 | Body lean mass | CAD | -0.009 | -0.007 | 0.002 | 0.009 | 3.60E-08 | 0.436 | NA | NA | NA | NA |
| rs281385 | Body lean mass | CAD | -0.014 | -0.002 | 0.002 | 0.013 | 5.33E-09 | 0.862 | NA | NA | NA | NA |
| rs2815753 | Body lean mass | CAD | 0.010 | 0.010 | 0.002 | 0.010 | 7.42E-10 | 0.290 | NA | NA | NA | NA |
| rs28391281 | Body lean mass | CAD | -0.010 | 0.048 | 0.002 | 0.010 | 4.28E-10 | 0.000 | NA | NA | NA | NA |
| rs28413009 | Body lean mass | CAD | -0.015 | -0.010 | 0.003 | 0.016 | 4.26E-08 | 0.548 | NA | NA | NA | NA |
| rs284315 | Body lean mass | CAD | -0.010 | -0.014 | 0.002 | 0.010 | 1.39E-11 | 0.184 | NA | NA | NA | NA |
| rs28457693 | Body lean mass | CAD | 0.027 | -0.035 | 0.002 | 0.015 | 1.04E-27 | 0.020 | NA | NA | NA | NA |
| rs2866719 | Body lean mass | CAD | 0.009 | 0.010 | 0.002 | 0.010 | 4.27E-08 | 0.286 | NA | NA | NA | NA |
| rs2885697 | Body lean mass | CAD | -0.020 | -0.013 | 0.002 | 0.010 | 9.77E-35 | 0.197 | NA | NA | NA | NA |
| rs28929474 | Body lean mass | CAD | 0.034 | -0.147 | 0.005 | 0.045 | 3.00E-10 | 0.001 | NA | NA | NA | NA |
| rs2897968 | Body lean mass | CAD | 0.016 | 0.004 | 0.002 | 0.010 | 2.38E-23 | 0.681 | NA | NA | NA | NA |
| rs2900208 | Body lean mass | CAD | 0.014 | 0.002 | 0.002 | 0.010 | 1.10E-17 | 0.802 | NA | NA | NA | NA |
| rs292168 | Body lean mass | CAD | -0.014 | 0.011 | 0.002 | 0.009 | 8.64E-20 | 0.236 | NA | NA | NA | NA |
| rs2979655 | Body lean mass | CAD | 0.012 | 0.018 | 0.002 | 0.011 | 8.58E-09 | 0.116 | NA | NA | NA | NA |
| rs299370 | Body lean mass | CAD | -0.010 | -0.004 | 0.002 | 0.010 | 1.00E-09 | 0.667 | NA | NA | NA | NA |
| rs30235 | Body lean mass | CAD | 0.009 | -0.018 | 0.002 | 0.010 | 7.28E-09 | 0.056 | NA | NA | NA | NA |
| rs310302 | Body lean mass | CAD | -0.009 | -0.006 | 0.002 | 0.009 | 1.81E-08 | 0.504 | NA | NA | NA | NA |
| rs310796 | Body lean mass | CAD | 0.011 | -0.022 | 0.002 | 0.010 | 5.26E-12 | 0.028 | NA | NA | NA | NA |
| rs3110496 | Body lean mass | CAD | 0.010 | -0.017 | 0.002 | 0.010 | 6.21E-09 | 0.081 | NA | NA | NA | NA |
| rs3116201 | Body lean mass | CAD | -0.018 | -0.016 | 0.003 | 0.017 | 2.47E-12 | 0.347 | NA | NA | NA | NA |
| rs3118915 | Body lean mass | CAD | -0.032 | 0.026 | 0.002 | 0.012 | 2.32E-64 | 0.033 | NA | NA | NA | NA |
| rs3212260 | Body lean mass | CAD | 0.013 | -0.007 | 0.002 | 0.011 | 5.69E-14 | 0.511 | NA | NA | NA | NA |
| rs324010 | Body lean mass | CAD | -0.009 | -0.010 | 0.002 | 0.010 | 1.87E-08 | 0.288 | NA | NA | NA | NA |
| rs33967909 | Body lean mass | CAD | 0.015 | 0.008 | 0.002 | 0.011 | 3.17E-16 | 0.486 | NA | NA | NA | NA |
| rs34028346 | Body lean mass | CAD | 0.012 | 0.005 | 0.002 | 0.013 | 3.00E-08 | 0.680 | NA | NA | NA | NA |
| rs343935 | Body lean mass | CAD | 0.013 | 0.003 | 0.002 | 0.013 | 3.27E-10 | 0.843 | NA | NA | NA | NA |
| rs34517439 | Body lean mass | CAD | 0.036 | 0.038 | 0.002 | 0.019 | 2.46E-52 | 0.042 | NA | NA | NA | NA |
| rs34693680 | Body lean mass | CAD | 0.014 | -0.016 | 0.002 | 0.015 | 9.82E-10 | 0.293 | NA | NA | NA | NA |
| rs34776209 | Body lean mass | CAD | -0.018 | 0.001 | 0.002 | 0.011 | 4.01E-25 | 0.949 | NA | NA | NA | NA |
| rs34879158 | Body lean mass | CAD | -0.021 | -0.006 | 0.002 | 0.011 | 4.56E-34 | 0.559 | NA | NA | NA | NA |
| rs35233301 | Body lean mass | CAD | 0.011 | 0.003 | 0.002 | 0.010 | 5.02E-11 | 0.788 | NA | NA | NA | NA |
| rs35276559 | Body lean mass | CAD | -0.009 | -0.013 | 0.002 | 0.011 | 4.82E-08 | 0.234 | NA | NA | NA | NA |
| rs35309034 | Body lean mass | CAD | -0.017 | 0.023 | 0.002 | 0.011 | 4.24E-21 | 0.036 | NA | NA | NA | NA |
| rs35436119 | Body lean mass | CAD | 0.018 | 0.034 | 0.003 | 0.019 | 1.25E-09 | 0.084 | NA | NA | NA | NA |
| rs35506085 | Body lean mass | CAD | -0.020 | 0.008 | 0.002 | 0.012 | 2.29E-22 | 0.513 | NA | NA | NA | NA |
| rs35665085 | Body lean mass | CAD | -0.019 | -0.040 | 0.003 | 0.029 | 1.17E-08 | 0.172 | NA | NA | NA | NA |
| rs35710322 | Body lean mass | CAD | 0.012 | -0.003 | 0.002 | 0.010 | 7.33E-16 | 0.775 | NA | NA | NA | NA |
| rs357501 | Body lean mass | CAD | 0.011 | 0.011 | 0.002 | 0.010 | 1.85E-11 | 0.266 | NA | NA | NA | NA |
| rs35756741 | Body lean mass | CAD | -0.019 | 0.007 | 0.003 | 0.016 | 1.69E-12 | 0.668 | NA | NA | NA | NA |
| rs35804313 | Body lean mass | CAD | -0.012 | -0.006 | 0.002 | 0.012 | 8.27E-10 | 0.632 | NA | NA | NA | NA |
| rs35874463 | Body lean mass | CAD | 0.022 | 0.056 | 0.003 | 0.030 | 1.20E-11 | 0.067 | NA | NA | NA | Body fat distribution |
| rs36000545 | Body lean mass | CAD | -0.015 | -0.011 | 0.002 | 0.011 | 2.01E-21 | 0.358 | NA | NA | NA | NA |
| rs36100359 | Body lean mass | CAD | -0.013 | 0.016 | 0.002 | 0.014 | 1.06E-08 | 0.246 | NA | NA | NA | NA |
| rs3738449 | Body lean mass | CAD | -0.012 | 0.001 | 0.002 | 0.010 | 1.15E-12 | 0.927 | NA | NA | NA | NA |
| rs3740591 | Body lean mass | CAD | 0.013 | -0.015 | 0.002 | 0.011 | 9.64E-17 | 0.167 | NA | NA | NA | NA |
| rs3759094 | Body lean mass | CAD | -0.013 | -0.007 | 0.002 | 0.010 | 1.18E-14 | 0.530 | NA | NA | NA | NA |
| rs3765351 | Body lean mass | CAD | -0.011 | 0.014 | 0.002 | 0.009 | 1.56E-12 | 0.127 | NA | NA | NA | NA |
| rs3778157 | Body lean mass | CAD | 0.012 | 0.020 | 0.002 | 0.014 | 4.28E-09 | 0.158 | NA | NA | NA | NA |
| rs3783256 | Body lean mass | CAD | -0.010 | 0.005 | 0.002 | 0.010 | 1.41E-09 | 0.635 | NA | NA | NA | NA |
| rs3798519 | Body lean mass | CAD | 0.021 | 0.029 | 0.002 | 0.011 | 2.26E-25 | 0.013 | NA | NA | NA | NA |
| rs3800963 | Body lean mass | CAD | -0.009 | 0.008 | 0.002 | 0.010 | 9.65E-09 | 0.394 | NA | NA | NA | NA |
| rs3803286 | Body lean mass | CAD | -0.010 | -0.021 | 0.002 | 0.010 | 4.12E-09 | 0.030 | NA | NA | NA | NA |
| rs3809570 | Body lean mass | CAD | 0.013 | -0.009 | 0.002 | 0.011 | 8.54E-14 | 0.402 | NA | NA | NA | NA |
| rs3810291 | Body lean mass | CAD | 0.017 | 0.025 | 0.002 | 0.010 | 6.10E-25 | 0.018 | NA | NA | NA | NA |
| rs3814877 | Body lean mass | CAD | 0.023 | -0.007 | 0.002 | 0.009 | 1.89E-50 | 0.467 | NA | NA | NA | NA |
| rs3818416 | Body lean mass | CAD | 0.016 | -0.009 | 0.002 | 0.011 | 8.13E-18 | 0.422 | NA | NA | NA | NA |
| rs3843751 | Body lean mass | CAD | -0.014 | 0.046 | 0.002 | 0.010 | 3.01E-17 | 0.000 | NA | NA | NA | NA |
| rs3845344 | Body lean mass | CAD | 0.009 | -0.014 | 0.002 | 0.009 | 2.68E-08 | 0.143 | NA | NA | NA | NA |
| rs3853252 | Body lean mass | CAD | 0.016 | 0.008 | 0.002 | 0.009 | 9.61E-25 | 0.373 | NA | NA | NA | NA |
| rs390192 | Body lean mass | CAD | -0.009 | -0.002 | 0.002 | 0.009 | 1.75E-09 | 0.795 | NA | NA | NA | NA |
| rs4073717 | Body lean mass | CAD | -0.018 | 0.010 | 0.002 | 0.012 | 3.15E-21 | 0.375 | NA | NA | NA | NA |
| rs4076108 | Body lean mass | CAD | 0.010 | -0.001 | 0.002 | 0.012 | 2.08E-08 | 0.966 | NA | NA | NA | NA |
| rs41271299 | Body lean mass | CAD | 0.044 | -0.012 | 0.003 | 0.035 | 1.94E-36 | 0.740 | NA | NA | NA | Body fat distribution |
| rs41311445 | Body lean mass | CAD | -0.024 | 0.001 | 0.003 | 0.019 | 9.26E-21 | 0.953 | NA | NA | NA | NA |
| rs42044 | Body lean mass | CAD | 0.029 | -0.024 | 0.002 | 0.011 | 3.21E-61 | 0.028 | NA | NA | NA | NA |
| rs4235012 | Body lean mass | CAD | 0.009 | -0.015 | 0.002 | 0.010 | 6.70E-09 | 0.103 | NA | NA | NA | NA |
| rs4240326 | Body lean mass | CAD | -0.022 | -0.008 | 0.002 | 0.009 | 4.83E-46 | 0.412 | NA | NA | NA | NA |
| rs4282339 | Body lean mass | CAD | -0.017 | -0.006 | 0.002 | 0.011 | 5.97E-20 | 0.570 | NA | NA | NA | NA |
| rs4341996 | Body lean mass | CAD | -0.011 | 0.016 | 0.002 | 0.011 | 1.26E-08 | 0.149 | NA | NA | NA | NA |
| rs4472800 | Body lean mass | CAD | 0.010 | 0.011 | 0.002 | 0.010 | 2.19E-10 | 0.260 | NA | NA | NA | NA |
| rs4627713 | Body lean mass | CAD | 0.032 | -0.089 | 0.005 | 0.030 | 4.69E-12 | 0.003 | NA | NA | NA | NA |
| rs4635681 | Body lean mass | CAD | 0.012 | 0.009 | 0.002 | 0.013 | 7.23E-09 | 0.494 | NA | NA | NA | NA |
| rs4680 | Body lean mass | CAD | 0.008 | 0.025 | 0.002 | 0.009 | 3.69E-08 | 0.007 | NA | NA | NA | NA |
| rs4752689 | Body lean mass | CAD | 0.011 | -0.015 | 0.002 | 0.010 | 3.47E-13 | 0.128 | NA | NA | NA | NA |
| rs475591 | Body lean mass | CAD | 0.011 | -0.002 | 0.002 | 0.010 | 2.55E-12 | 0.840 | NA | NA | NA | NA |
| rs4782286 | Body lean mass | CAD | -0.012 | 0.012 | 0.002 | 0.011 | 9.64E-11 | 0.302 | NA | NA | NA | NA |
| rs4800670 | Body lean mass | CAD | -0.009 | -0.005 | 0.002 | 0.010 | 2.55E-08 | 0.581 | NA | NA | NA | NA |
| rs4819021 | Body lean mass | CAD | -0.010 | -0.003 | 0.002 | 0.010 | 9.95E-11 | 0.727 | NA | NA | NA | NA |
| rs4858697 | Body lean mass | CAD | 0.012 | 0.003 | 0.002 | 0.009 | 1.21E-15 | 0.724 | NA | NA | NA | NA |
| rs4865956 | Body lean mass | CAD | -0.011 | -0.006 | 0.002 | 0.010 | 1.11E-11 | 0.570 | NA | NA | NA | NA |
| rs4980067 | Body lean mass | CAD | -0.012 | 0.017 | 0.002 | 0.010 | 3.21E-14 | 0.077 | NA | NA | NA | NA |
| rs4980826 | Body lean mass | CAD | 0.009 | -0.001 | 0.002 | 0.010 | 3.75E-08 | 0.948 | NA | NA | NA | NA |
| rs4985148 | Body lean mass | CAD | -0.011 | 0.006 | 0.002 | 0.010 | 1.85E-11 | 0.539 | NA | NA | NA | NA |
| rs508347 | Body lean mass | CAD | -0.013 | -0.004 | 0.002 | 0.010 | 3.71E-15 | 0.695 | NA | NA | NA | NA |
| rs509035 | Body lean mass | CAD | 0.018 | -0.021 | 0.002 | 0.010 | 2.84E-28 | 0.039 | NA | NA | NA | NA |
| rs545608 | Body lean mass | CAD | 0.026 | 0.007 | 0.002 | 0.012 | 7.65E-44 | 0.559 | NA | NA | NA | Body fat distribution |
| rs55681913 | Body lean mass | CAD | 0.028 | -0.017 | 0.003 | 0.018 | 1.76E-28 | 0.364 | NA | NA | NA | NA |
| rs55726687 | Body lean mass | CAD | 0.015 | 0.006 | 0.002 | 0.012 | 4.96E-15 | 0.627 | NA | NA | NA | NA |
| rs55758152 | Body lean mass | CAD | 0.012 | -0.016 | 0.002 | 0.012 | 6.27E-13 | 0.196 | NA | NA | NA | NA |
| rs55831773 | Body lean mass | CAD | -0.016 | -0.002 | 0.002 | 0.013 | 6.89E-16 | 0.853 | NA | NA | NA | NA |
| rs55872725 | Body lean mass | CAD | 0.037 | 0.030 | 0.002 | 0.010 | 8.60E-121 | 0.002 | NA | NA | NA | Body fat percentage, Body fat distribution |
| rs57126421 | Body lean mass | CAD | -0.010 | 0.005 | 0.002 | 0.011 | 8.23E-09 | 0.643 | NA | NA | NA | NA |
| rs57153895 | Body lean mass | CAD | -0.012 | -0.020 | 0.002 | 0.010 | 3.41E-14 | 0.037 | NA | NA | NA | NA |
| rs5742915 | Body lean mass | CAD | 0.010 | -0.006 | 0.002 | 0.010 | 5.54E-10 | 0.540 | NA | NA | NA | NA |
| rs5752989 | Body lean mass | CAD | -0.010 | 0.006 | 0.002 | 0.010 | 4.14E-11 | 0.571 | NA | NA | NA | NA |
| rs57635800 | Body lean mass | CAD | 0.013 | 0.022 | 0.002 | 0.010 | 1.35E-13 | 0.034 | NA | NA | NA | NA |
| rs57636386 | Body lean mass | CAD | -0.025 | -0.034 | 0.003 | 0.016 | 9.95E-19 | 0.032 | NA | NA | NA | NA |
| rs58857770 | Body lean mass | CAD | -0.012 | -0.018 | 0.002 | 0.010 | 7.85E-14 | 0.064 | NA | NA | NA | NA |
| rs599004 | Body lean mass | CAD | -0.015 | -0.008 | 0.002 | 0.010 | 1.77E-17 | 0.435 | NA | NA | NA | NA |
| rs59985551 | Body lean mass | CAD | -0.018 | 0.004 | 0.002 | 0.011 | 1.50E-22 | 0.746 | NA | NA | NA | NA |
| rs60077625 | Body lean mass | CAD | 0.014 | 0.002 | 0.002 | 0.010 | 4.09E-18 | 0.840 | NA | NA | NA | NA |
| rs6032233 | Body lean mass | CAD | 0.012 | 0.006 | 0.002 | 0.011 | 2.58E-10 | 0.617 | NA | NA | NA | NA |
| rs607472 | Body lean mass | CAD | 0.012 | -0.001 | 0.002 | 0.010 | 1.44E-13 | 0.907 | NA | NA | NA | NA |
| rs60804050 | Body lean mass | CAD | -0.011 | 0.021 | 0.002 | 0.010 | 1.16E-09 | 0.040 | NA | NA | NA | NA |
| rs6081869 | Body lean mass | CAD | -0.010 | -0.010 | 0.002 | 0.010 | 1.82E-10 | 0.311 | NA | NA | NA | NA |
| rs6085659 | Body lean mass | CAD | -0.010 | 0.014 | 0.002 | 0.010 | 8.95E-10 | 0.146 | NA | NA | NA | NA |
| rs6142059 | Body lean mass | CAD | 0.011 | 0.005 | 0.002 | 0.009 | 7.89E-13 | 0.554 | NA | NA | NA | NA |
| rs61729527 | Body lean mass | CAD | -0.025 | 0.008 | 0.003 | 0.027 | 5.45E-13 | 0.760 | NA | NA | NA | NA |
| rs61862463 | Body lean mass | CAD | 0.030 | 0.025 | 0.005 | 0.032 | 8.09E-09 | 0.438 | NA | NA | NA | NA |
| rs61941043 | Body lean mass | CAD | 0.049 | -0.065 | 0.008 | 0.048 | 8.01E-10 | 0.174 | NA | NA | NA | NA |
| rs61954257 | Body lean mass | CAD | 0.012 | -0.001 | 0.002 | 0.009 | 2.36E-14 | 0.916 | NA | NA | NA | NA |
| rs62070645 | Body lean mass | CAD | -0.024 | 0.021 | 0.002 | 0.011 | 3.28E-44 | 0.070 | NA | NA | NA | NA |
| rs62107261 | Body lean mass | CAD | -0.054 | -0.021 | 0.004 | 0.034 | 7.00E-51 | 0.526 | NA | NA | NA | Body fat distribution |
| rs62246314 | Body lean mass | CAD | 0.014 | -0.017 | 0.003 | 0.017 | 4.72E-08 | 0.330 | NA | NA | NA | NA |
| rs62275882 | Body lean mass | CAD | -0.014 | 0.034 | 0.002 | 0.014 | 1.63E-10 | 0.021 | NA | NA | NA | NA |
| rs6235 | Body lean mass | CAD | 0.016 | -0.001 | 0.002 | 0.010 | 4.14E-20 | 0.954 | NA | NA | NA | NA |
| rs62515437 | Body lean mass | CAD | 0.020 | 0.015 | 0.002 | 0.012 | 1.10E-28 | 0.231 | NA | NA | NA | NA |
| rs62621197 | Body lean mass | CAD | -0.036 | -0.048 | 0.004 | 0.046 | 9.31E-18 | 0.289 | NA | NA | NA | Body fat distribution |
| rs62621812 | Body lean mass | CAD | 0.049 | 0.025 | 0.006 | 0.041 | 1.20E-18 | 0.552 | NA | NA | NA | NA |
| rs6445198 | Body lean mass | CAD | -0.009 | -0.011 | 0.002 | 0.009 | 3.08E-09 | 0.248 | NA | NA | NA | NA |
| rs6505781 | Body lean mass | CAD | -0.010 | 0.005 | 0.002 | 0.010 | 2.65E-08 | 0.659 | NA | NA | NA | NA |
| rs6570509 | Body lean mass | CAD | -0.018 | 0.004 | 0.002 | 0.010 | 1.55E-25 | 0.717 | NA | NA | NA | NA |
| rs658957 | Body lean mass | CAD | -0.012 | -0.007 | 0.002 | 0.011 | 1.80E-08 | 0.502 | NA | NA | NA | NA |
| rs662115 | Body lean mass | CAD | 0.010 | 0.017 | 0.002 | 0.009 | 4.22E-10 | 0.070 | NA | NA | NA | NA |
| rs66922415 | Body lean mass | CAD | 0.044 | 0.056 | 0.002 | 0.011 | 6.18E-130 | 0.000 | NA | NA | NA | NA |
| rs67551338 | Body lean mass | CAD | 0.025 | 0.014 | 0.003 | 0.022 | 5.58E-15 | 0.522 | NA | NA | NA | NA |
| rs6762578 | Body lean mass | CAD | 0.016 | -0.032 | 0.002 | 0.012 | 6.68E-19 | 0.007 | NA | NA | NA | NA |
| rs6800021 | Body lean mass | CAD | 0.014 | 0.021 | 0.002 | 0.010 | 1.13E-19 | 0.041 | NA | NA | NA | NA |
| rs6821305 | Body lean mass | CAD | 0.012 | -0.005 | 0.002 | 0.009 | 8.05E-15 | 0.560 | NA | NA | NA | NA |
| rs6873192 | Body lean mass | CAD | -0.012 | -0.029 | 0.002 | 0.010 | 3.90E-15 | 0.003 | NA | NA | NA | NA |
| rs6874142 | Body lean mass | CAD | 0.020 | 0.020 | 0.003 | 0.023 | 2.13E-15 | 0.388 | NA | NA | NA | NA |
| rs6902789 | Body lean mass | CAD | 0.010 | -0.009 | 0.002 | 0.010 | 1.10E-10 | 0.347 | NA | NA | NA | NA |
| rs6975015 | Body lean mass | CAD | 0.018 | 0.002 | 0.002 | 0.015 | 1.68E-13 | 0.890 | NA | NA | NA | NA |
| rs7047694 | Body lean mass | CAD | 0.011 | 0.008 | 0.002 | 0.010 | 1.30E-11 | 0.426 | NA | NA | NA | NA |
| rs7077783 | Body lean mass | CAD | -0.015 | -0.013 | 0.002 | 0.014 | 1.36E-11 | 0.347 | NA | NA | NA | NA |
| rs7080472 | Body lean mass | CAD | 0.015 | 0.026 | 0.002 | 0.009 | 2.28E-22 | 0.006 | NA | NA | NA | NA |
| rs7097872 | Body lean mass | CAD | 0.012 | -0.037 | 0.002 | 0.009 | 2.19E-14 | 0.000 | NA | NA | NA | NA |
| rs7129320 | Body lean mass | CAD | -0.023 | 0.027 | 0.002 | 0.013 | 7.00E-30 | 0.047 | NA | NA | NA | NA |
| rs7132908 | Body lean mass | CAD | 0.017 | 0.011 | 0.002 | 0.010 | 7.52E-26 | 0.267 | NA | NA | NA | NA |
| rs7134283 | Body lean mass | CAD | -0.013 | 0.004 | 0.002 | 0.011 | 1.98E-13 | 0.696 | NA | NA | NA | NA |
| rs71385734 | Body lean mass | CAD | -0.024 | 0.000 | 0.002 | 0.016 | 3.58E-31 | 0.989 | NA | NA | NA | NA |
| rs7141420 | Body lean mass | CAD | 0.012 | 0.007 | 0.002 | 0.009 | 1.65E-14 | 0.450 | NA | NA | NA | NA |
| rs71414738 | Body lean mass | CAD | 0.011 | -0.007 | 0.002 | 0.014 | 1.55E-08 | 0.610 | NA | NA | NA | NA |
| rs71484923 | Body lean mass | CAD | 0.011 | -0.006 | 0.002 | 0.013 | 1.99E-08 | 0.658 | NA | NA | NA | NA |
| rs7148516 | Body lean mass | CAD | -0.009 | 0.001 | 0.002 | 0.010 | 1.53E-08 | 0.959 | NA | NA | NA | NA |
| rs7190477 | Body lean mass | CAD | 0.009 | -0.031 | 0.002 | 0.010 | 1.93E-09 | 0.003 | NA | NA | NA | NA |
| rs7205337 | Body lean mass | CAD | 0.016 | 0.028 | 0.002 | 0.011 | 3.95E-17 | 0.014 | NA | NA | NA | NA |
| rs7214743 | Body lean mass | CAD | -0.016 | 0.022 | 0.002 | 0.010 | 2.11E-22 | 0.023 | NA | NA | NA | NA |
| rs7229351 | Body lean mass | CAD | -0.010 | 0.025 | 0.002 | 0.010 | 5.53E-11 | 0.011 | NA | NA | NA | NA |
| rs723149 | Body lean mass | CAD | -0.013 | 0.007 | 0.002 | 0.010 | 9.40E-17 | 0.472 | NA | NA | NA | NA |
| rs7235010 | Body lean mass | CAD | 0.027 | 0.002 | 0.002 | 0.011 | 2.75E-47 | 0.881 | NA | NA | NA | NA |
| rs724016 | Body lean mass | CAD | 0.030 | -0.018 | 0.002 | 0.009 | 9.22E-84 | 0.056 | NA | NA | NA | NA |
| rs7250927 | Body lean mass | CAD | -0.011 | 0.001 | 0.002 | 0.011 | 3.22E-11 | 0.932 | NA | NA | NA | NA |
| rs72656010 | Body lean mass | CAD | -0.036 | 0.019 | 0.002 | 0.014 | 5.13E-55 | 0.191 | NA | NA | NA | NA |
| rs72660086 | Body lean mass | CAD | 0.015 | 0.018 | 0.002 | 0.012 | 3.61E-15 | 0.130 | NA | NA | NA | NA |
| rs72699866 | Body lean mass | CAD | 0.013 | 0.011 | 0.002 | 0.013 | 1.09E-11 | 0.386 | NA | NA | NA | NA |
| rs72703414 | Body lean mass | CAD | -0.018 | 0.017 | 0.003 | 0.018 | 1.20E-08 | 0.341 | NA | NA | NA | NA |
| rs72755233 | Body lean mass | CAD | -0.014 | -0.004 | 0.002 | 0.021 | 1.17E-08 | 0.833 | NA | NA | NA | Body fat distribution |
| rs72801843 | Body lean mass | CAD | 0.016 | -0.009 | 0.002 | 0.011 | 1.99E-21 | 0.431 | NA | NA | NA | NA |
| rs72828807 | Body lean mass | CAD | 0.010 | -0.005 | 0.002 | 0.010 | 6.29E-09 | 0.614 | NA | NA | NA | NA |
| rs72885917 | Body lean mass | CAD | -0.022 | 0.028 | 0.002 | 0.012 | 2.70E-36 | 0.020 | NA | NA | NA | NA |
| rs73052033 | Body lean mass | CAD | -0.016 | -0.004 | 0.002 | 0.012 | 3.10E-16 | 0.750 | NA | NA | NA | NA |
| rs730536 | Body lean mass | CAD | -0.009 | 0.022 | 0.002 | 0.011 | 2.50E-08 | 0.045 | NA | NA | NA | NA |
| rs7305516 | Body lean mass | CAD | -0.011 | -0.002 | 0.002 | 0.010 | 2.19E-12 | 0.799 | NA | NA | NA | NA |
| rs73175572 | Body lean mass | CAD | 0.028 | -0.034 | 0.002 | 0.017 | 1.56E-29 | 0.048 | NA | NA | NA | NA |
| rs73619441 | Body lean mass | CAD | -0.016 | 0.006 | 0.002 | 0.013 | 1.49E-13 | 0.655 | NA | NA | NA | NA |
| rs73966422 | Body lean mass | CAD | 0.013 | 0.004 | 0.002 | 0.014 | 4.67E-08 | 0.790 | NA | NA | NA | NA |
| rs74048171 | Body lean mass | CAD | -0.010 | -0.002 | 0.002 | 0.011 | 1.05E-08 | 0.837 | NA | NA | NA | NA |
| rs74494415 | Body lean mass | CAD | -0.030 | -0.015 | 0.004 | 0.022 | 2.53E-14 | 0.497 | NA | NA | NA | NA |
| rs74565893 | Body lean mass | CAD | -0.040 | -0.016 | 0.007 | 0.022 | 3.41E-08 | 0.457 | NA | NA | NA | NA |
| rs7460093 | Body lean mass | CAD | 0.009 | 0.001 | 0.002 | 0.011 | 1.93E-09 | 0.926 | NA | NA | NA | NA |
| rs748457 | Body lean mass | CAD | 0.009 | -0.007 | 0.002 | 0.009 | 1.58E-09 | 0.445 | NA | NA | NA | NA |
| rs7559547 | Body lean mass | CAD | 0.032 | 0.034 | 0.002 | 0.012 | 6.60E-57 | 0.004 | NA | NA | NA | NA |
| rs7584391 | Body lean mass | CAD | -0.012 | -0.006 | 0.002 | 0.012 | 4.90E-09 | 0.618 | NA | NA | NA | NA |
| rs7624428 | Body lean mass | CAD | 0.011 | 0.006 | 0.002 | 0.011 | 8.34E-10 | 0.605 | NA | NA | NA | NA |
| rs7628343 | Body lean mass | CAD | 0.015 | -0.011 | 0.003 | 0.013 | 5.27E-09 | 0.366 | NA | NA | NA | NA |
| rs76798800 | Body lean mass | CAD | 0.023 | -0.038 | 0.002 | 0.012 | 1.27E-38 | 0.001 | NA | NA | NA | NA |
| rs768023 | Body lean mass | CAD | 0.019 | 0.001 | 0.002 | 0.010 | 2.93E-33 | 0.944 | NA | NA | NA | NA |
| rs76895963 | Body lean mass | CAD | 0.103 | 0.000 | 0.006 | 0.047 | 7.25E-67 | 0.999 | NA | NA | NA | NA |
| rs7728690 | Body lean mass | CAD | -0.011 | 0.001 | 0.002 | 0.010 | 3.26E-12 | 0.927 | NA | NA | NA | NA |
| rs7730885 | Body lean mass | CAD | 0.015 | -0.003 | 0.002 | 0.010 | 3.16E-20 | 0.783 | NA | NA | NA | NA |
| rs7731023 | Body lean mass | CAD | 0.010 | -0.001 | 0.002 | 0.010 | 7.59E-10 | 0.905 | NA | NA | NA | NA |
| rs77392989 | Body lean mass | CAD | -0.014 | -0.007 | 0.002 | 0.013 | 5.47E-12 | 0.598 | NA | NA | NA | NA |
| rs7740107 | Body lean mass | CAD | -0.029 | 0.030 | 0.002 | 0.011 | 4.97E-61 | 0.006 | NA | NA | NA | NA |
| rs7781964 | Body lean mass | CAD | 0.013 | -0.045 | 0.002 | 0.011 | 1.35E-11 | 0.000 | NA | NA | NA | NA |
| rs7815955 | Body lean mass | CAD | -0.017 | 0.013 | 0.002 | 0.011 | 1.09E-19 | 0.227 | NA | NA | NA | NA |
| rs78378222 | Body lean mass | CAD | 0.089 | 0.026 | 0.007 | 0.054 | 9.38E-37 | 0.628 | NA | NA | NA | NA |
| rs78812993 | Body lean mass | CAD | -0.023 | -0.010 | 0.003 | 0.024 | 4.36E-11 | 0.693 | NA | NA | NA | NA |
| rs78818722 | Body lean mass | CAD | 0.023 | 0.012 | 0.003 | 0.026 | 3.03E-14 | 0.642 | NA | NA | NA | NA |
| rs78964719 | Body lean mass | CAD | 0.017 | 0.024 | 0.003 | 0.019 | 1.92E-08 | 0.214 | NA | NA | NA | NA |
| rs7910087 | Body lean mass | CAD | -0.012 | 0.006 | 0.002 | 0.009 | 2.80E-15 | 0.530 | NA | NA | NA | NA |
| rs79266482 | Body lean mass | CAD | 0.010 | 0.005 | 0.002 | 0.013 | 4.05E-08 | 0.708 | NA | NA | NA | NA |
| rs7952436 | Body lean mass | CAD | -0.033 | 0.043 | 0.003 | 0.026 | 3.09E-32 | 0.099 | NA | NA | NA | NA |
| rs7977788 | Body lean mass | CAD | 0.026 | 0.005 | 0.002 | 0.011 | 5.52E-46 | 0.677 | NA | NA | NA | NA |
| rs79883557 | Body lean mass | CAD | -0.018 | 0.028 | 0.003 | 0.018 | 1.97E-10 | 0.112 | NA | NA | NA | NA |
| rs7994814 | Body lean mass | CAD | 0.011 | -0.032 | 0.002 | 0.012 | 2.36E-12 | 0.009 | NA | NA | NA | NA |
| rs8007058 | Body lean mass | CAD | 0.010 | 0.008 | 0.002 | 0.011 | 4.13E-08 | 0.471 | NA | NA | NA | NA |
| rs8007644 | Body lean mass | CAD | 0.010 | 0.003 | 0.002 | 0.010 | 2.14E-09 | 0.781 | NA | NA | NA | NA |
| rs8059189 | Body lean mass | CAD | -0.011 | -0.007 | 0.002 | 0.010 | 2.73E-12 | 0.470 | NA | NA | NA | NA |
| rs8123912 | Body lean mass | CAD | 0.011 | -0.006 | 0.002 | 0.012 | 5.26E-10 | 0.617 | NA | NA | NA | NA |
| rs815341 | Body lean mass | CAD | 0.010 | 0.032 | 0.002 | 0.010 | 2.24E-09 | 0.001 | TRUE | rs815341 | rs2990998 | NA |
| rs836519 | Body lean mass | CAD | 0.012 | 0.014 | 0.002 | 0.011 | 2.74E-10 | 0.202 | NA | NA | NA | NA |
| rs9314420 | Body lean mass | CAD | -0.009 | -0.006 | 0.002 | 0.011 | 4.36E-09 | 0.604 | NA | NA | NA | NA |
| rs9317002 | Body lean mass | CAD | 0.009 | -0.007 | 0.002 | 0.009 | 2.39E-09 | 0.489 | NA | NA | NA | NA |
| rs931874 | Body lean mass | CAD | 0.009 | 0.012 | 0.002 | 0.010 | 5.23E-09 | 0.236 | NA | NA | NA | NA |
| rs9327336 | Body lean mass | CAD | 0.010 | 0.014 | 0.002 | 0.010 | 7.42E-10 | 0.157 | NA | NA | NA | NA |
| rs9343977 | Body lean mass | CAD | -0.013 | 0.013 | 0.002 | 0.009 | 7.02E-18 | 0.176 | NA | NA | NA | NA |
| rs9379084 | Body lean mass | CAD | -0.015 | 0.017 | 0.002 | 0.016 | 1.25E-09 | 0.294 | NA | NA | NA | NA |
| rs9381350 | Body lean mass | CAD | 0.010 | -0.009 | 0.002 | 0.010 | 3.95E-09 | 0.358 | NA | NA | NA | NA |
| rs946197 | Body lean mass | CAD | 0.016 | -0.013 | 0.002 | 0.010 | 3.92E-18 | 0.217 | NA | NA | NA | NA |
| rs9512661 | Body lean mass | CAD | -0.009 | -0.003 | 0.002 | 0.010 | 1.61E-08 | 0.755 | NA | NA | NA | NA |
| rs9540493 | Body lean mass | CAD | -0.008 | -0.006 | 0.002 | 0.010 | 4.66E-08 | 0.539 | NA | NA | NA | NA |
| rs9788443 | Body lean mass | CAD | 0.020 | -0.008 | 0.004 | 0.022 | 3.73E-08 | 0.713 | NA | NA | NA | NA |
| rs981002 | Body lean mass | CAD | -0.011 | 0.009 | 0.002 | 0.010 | 9.01E-11 | 0.354 | NA | NA | NA | NA |
| rs9826759 | Body lean mass | CAD | 0.015 | -0.009 | 0.002 | 0.010 | 1.92E-19 | 0.328 | NA | NA | NA | NA |
| rs9861443 | Body lean mass | CAD | 0.012 | 0.013 | 0.002 | 0.011 | 3.26E-12 | 0.236 | NA | NA | NA | NA |
| rs9915532 | Body lean mass | CAD | -0.016 | -0.008 | 0.002 | 0.012 | 2.57E-15 | 0.496 | NA | NA | NA | NA |
| rs9925273 | Body lean mass | CAD | -0.013 | 0.000 | 0.002 | 0.014 | 1.10E-10 | 0.990 | NA | NA | NA | NA |
| rs9951619 | Body lean mass | CAD | 0.013 | 0.002 | 0.002 | 0.011 | 2.38E-12 | 0.842 | NA | NA | NA | NA |
| rs9960619 | Body lean mass | CAD | 0.009 | -0.005 | 0.002 | 0.010 | 1.36E-08 | 0.609 | NA | NA | NA | NA |
| rs9985795 | Body lean mass | CAD | -0.009 | 0.007 | 0.002 | 0.010 | 3.95E-09 | 0.452 | NA | NA | NA | NA |
| rs10139160 | Hand grip strength (left) | CAD | 0.010 | -0.003 | 0.002 | 0.010 | 5.63E-09 | 0.756 | NA | NA | NA | NA |
| rs10205394 | Hand grip strength (left) | CAD | -0.012 | 0.010 | 0.002 | 0.011 | 2.38E-08 | 0.389 | NA | NA | NA | NA |
| rs10210654 | Hand grip strength (left) | CAD | 0.014 | -0.001 | 0.002 | 0.012 | 3.19E-10 | 0.922 | NA | NA | NA | NA |
| rs10403906 | Hand grip strength (left) | CAD | -0.010 | 0.007 | 0.002 | 0.010 | 2.87E-09 | 0.438 | NA | NA | NA | NA |
| rs10753139 | Hand grip strength (left) | CAD | 0.015 | -0.002 | 0.002 | 0.009 | 1.06E-16 | 0.867 | NA | NA | NA | NA |
| rs10753823 | Hand grip strength (left) | CAD | -0.011 | -0.006 | 0.002 | 0.010 | 1.51E-08 | 0.525 | NA | NA | NA | NA |
| rs10788958 | Hand grip strength (left) | CAD | 0.015 | 0.010 | 0.002 | 0.010 | 9.23E-16 | 0.293 | NA | NA | NA | NA |
| rs10805877 | Hand grip strength (left) | CAD | 0.012 | -0.006 | 0.002 | 0.012 | 5.08E-09 | 0.627 | NA | NA | NA | NA |
| rs11002322 | Hand grip strength (left) | CAD | -0.010 | 0.022 | 0.002 | 0.010 | 1.68E-08 | 0.029 | NA | NA | NA | NA |
| rs11124957 | Hand grip strength (left) | CAD | -0.011 | -0.006 | 0.002 | 0.009 | 7.23E-11 | 0.555 | NA | NA | NA | NA |
| rs11130333 | Hand grip strength (left) | CAD | 0.011 | -0.043 | 0.002 | 0.010 | 1.36E-09 | 0.000 | NA | NA | NA | NA |
| rs11236203 | Hand grip strength (left) | CAD | -0.015 | -0.004 | 0.002 | 0.010 | 1.33E-17 | 0.683 | TRUE | rs11236203 | rs76704686 | NA |
| rs11631697 | Hand grip strength (left) | CAD | 0.011 | 0.012 | 0.002 | 0.010 | 2.39E-08 | 0.241 | NA | NA | NA | NA |
| rs11642430 | Hand grip strength (left) | CAD | 0.011 | -0.008 | 0.002 | 0.010 | 2.64E-09 | 0.402 | NA | NA | NA | NA |
| rs11906450 | Hand grip strength (left) | CAD | 0.011 | -0.022 | 0.002 | 0.012 | 1.50E-08 | 0.070 | NA | NA | NA | NA |
| rs12055234 | Hand grip strength (left) | CAD | -0.010 | 0.005 | 0.002 | 0.010 | 4.39E-08 | 0.655 | NA | NA | NA | NA |
| rs12119893 | Hand grip strength (left) | CAD | -0.015 | -0.004 | 0.003 | 0.015 | 1.52E-08 | 0.781 | NA | NA | NA | NA |
| rs12129704 | Hand grip strength (left) | CAD | -0.016 | -0.007 | 0.002 | 0.012 | 1.83E-13 | 0.585 | NA | NA | NA | NA |
| rs12316046 | Hand grip strength (left) | CAD | -0.019 | 0.015 | 0.002 | 0.010 | 2.06E-25 | 0.135 | NA | NA | NA | NA |
| rs12361415 | Hand grip strength (left) | CAD | 0.014 | -0.006 | 0.002 | 0.011 | 1.29E-12 | 0.596 | NA | NA | NA | NA |
| rs12414407 | Hand grip strength (left) | CAD | 0.012 | -0.014 | 0.002 | 0.010 | 1.40E-10 | 0.140 | NA | NA | NA | NA |
| rs12673062 | Hand grip strength (left) | CAD | -0.013 | 0.010 | 0.002 | 0.012 | 3.29E-09 | 0.412 | NA | NA | NA | NA |
| rs12792358 | Hand grip strength (left) | CAD | -0.025 | 0.034 | 0.004 | 0.025 | 1.02E-12 | 0.185 | NA | NA | NA | NA |
| rs12917449 | Hand grip strength (left) | CAD | -0.013 | 0.028 | 0.002 | 0.012 | 3.19E-09 | 0.021 | NA | NA | NA | NA |
| rs12926737 | Hand grip strength (left) | CAD | -0.017 | 0.011 | 0.002 | 0.015 | 2.86E-14 | 0.430 | NA | NA | NA | NA |
| rs13107325 | Hand grip strength (left) | CAD | -0.028 | -0.007 | 0.003 | 0.022 | 6.10E-17 | 0.765 | NA | NA | NA | Body fat distribution |
| rs138019 | Hand grip strength (left) | CAD | -0.011 | -0.023 | 0.002 | 0.010 | 2.53E-08 | 0.030 | NA | NA | NA | NA |
| rs143384 | Hand grip strength (left) | CAD | 0.020 | -0.015 | 0.002 | 0.010 | 1.89E-28 | 0.120 | NA | NA | NA | Body fat distribution |
| rs1442883 | Hand grip strength (left) | CAD | -0.012 | 0.013 | 0.002 | 0.012 | 1.34E-09 | 0.271 | NA | NA | NA | NA |
| rs150330307 | Hand grip strength (left) | CAD | -0.031 | -0.027 | 0.005 | 0.031 | 3.35E-10 | 0.389 | NA | NA | NA | NA |
| rs1550115 | Hand grip strength (left) | CAD | 0.015 | -0.015 | 0.002 | 0.010 | 2.30E-14 | 0.150 | NA | NA | NA | NA |
| rs1551042 | Hand grip strength (left) | CAD | -0.011 | 0.004 | 0.002 | 0.010 | 5.59E-10 | 0.714 | NA | NA | NA | NA |
| rs1556659 | Hand grip strength (left) | CAD | 0.016 | -0.034 | 0.002 | 0.010 | 2.92E-19 | 0.001 | NA | NA | NA | NA |
| rs16896068 | Hand grip strength (left) | CAD | -0.021 | 0.021 | 0.002 | 0.012 | 4.93E-19 | 0.088 | NA | NA | NA | NA |
| rs217181 | Hand grip strength (left) | CAD | 0.013 | -0.024 | 0.002 | 0.012 | 1.03E-08 | 0.045 | NA | NA | NA | NA |
| rs2273555 | Hand grip strength (left) | CAD | 0.011 | 0.016 | 0.002 | 0.009 | 2.09E-09 | 0.082 | NA | NA | NA | NA |
| rs2532111 | Hand grip strength (left) | CAD | 0.011 | -0.012 | 0.002 | 0.010 | 5.04E-10 | 0.222 | NA | NA | NA | NA |
| rs2807504 | Hand grip strength (left) | CAD | 0.012 | 0.010 | 0.002 | 0.011 | 2.92E-09 | 0.371 | NA | NA | NA | NA |
| rs2871865 | Hand grip strength (left) | CAD | -0.024 | 0.053 | 0.003 | 0.015 | 2.96E-18 | 0.000 | NA | NA | NA | NA |
| rs2871960 | Hand grip strength (left) | CAD | 0.012 | -0.019 | 0.002 | 0.009 | 1.70E-11 | 0.041 | NA | NA | NA | NA |
| rs3116600 | Hand grip strength (left) | CAD | -0.018 | 0.026 | 0.002 | 0.012 | 1.11E-16 | 0.034 | NA | NA | NA | NA |
| rs3169733 | Hand grip strength (left) | CAD | 0.011 | 0.001 | 0.002 | 0.011 | 5.47E-09 | 0.936 | NA | NA | NA | NA |
| rs34159998 | Hand grip strength (left) | CAD | -0.024 | 0.013 | 0.004 | 0.031 | 1.60E-08 | 0.673 | NA | NA | NA | NA |
| rs34845616 | Hand grip strength (left) | CAD | 0.012 | 0.004 | 0.002 | 0.011 | 6.66E-09 | 0.749 | NA | NA | NA | NA |
| rs35779564 | Hand grip strength (left) | CAD | -0.010 | 0.024 | 0.002 | 0.010 | 3.78E-08 | 0.011 | NA | NA | NA | NA |
| rs35810656 | Hand grip strength (left) | CAD | 0.010 | 0.004 | 0.002 | 0.010 | 2.30E-08 | 0.680 | NA | NA | NA | NA |
| rs3785456 | Hand grip strength (left) | CAD | 0.015 | 0.004 | 0.002 | 0.010 | 1.92E-15 | 0.715 | NA | NA | NA | NA |
| rs41271299 | Hand grip strength (left) | CAD | 0.024 | -0.012 | 0.004 | 0.035 | 1.54E-09 | 0.740 | NA | NA | NA | Body fat percentage |
| rs41705 | Hand grip strength (left) | CAD | -0.012 | -0.010 | 0.002 | 0.011 | 7.29E-09 | 0.375 | NA | NA | NA | NA |
| rs417591 | Hand grip strength (left) | CAD | 0.012 | -0.002 | 0.002 | 0.011 | 7.30E-09 | 0.864 | NA | NA | NA | NA |
| rs4234519 | Hand grip strength (left) | CAD | -0.011 | -0.006 | 0.002 | 0.011 | 1.16E-08 | 0.595 | NA | NA | NA | NA |
| rs4308051 | Hand grip strength (left) | CAD | 0.017 | 0.004 | 0.002 | 0.012 | 6.67E-15 | 0.735 | NA | NA | NA | NA |
| rs4363950 | Hand grip strength (left) | CAD | 0.013 | -0.015 | 0.002 | 0.011 | 8.64E-11 | 0.191 | NA | NA | NA | NA |
| rs4380799 | Hand grip strength (left) | CAD | -0.012 | 0.045 | 0.002 | 0.020 | 4.93E-10 | 0.028 | NA | NA | NA | NA |
| rs4398863 | Hand grip strength (left) | CAD | -0.011 | 0.008 | 0.002 | 0.011 | 3.51E-08 | 0.467 | NA | NA | NA | NA |
| rs4553566 | Hand grip strength (left) | CAD | -0.011 | 0.018 | 0.002 | 0.009 | 1.93E-09 | 0.054 | NA | NA | NA | NA |
| rs4575361 | Hand grip strength (left) | CAD | -0.010 | -0.028 | 0.002 | 0.010 | 3.83E-08 | 0.008 | NA | NA | NA | NA |
| rs4621706 | Hand grip strength (left) | CAD | -0.013 | -0.009 | 0.002 | 0.009 | 8.03E-13 | 0.330 | NA | NA | NA | NA |
| rs4784329 | Hand grip strength (left) | CAD | -0.011 | -0.008 | 0.002 | 0.010 | 6.28E-10 | 0.395 | NA | NA | NA | NA |
| rs4886778 | Hand grip strength (left) | CAD | 0.013 | 0.017 | 0.002 | 0.009 | 9.52E-14 | 0.071 | NA | NA | NA | NA |
| rs56187488 | Hand grip strength (left) | CAD | -0.011 | -0.009 | 0.002 | 0.010 | 3.59E-08 | 0.368 | NA | NA | NA | NA |
| rs56338231 | Hand grip strength (left) | CAD | -0.012 | 0.005 | 0.002 | 0.011 | 4.35E-09 | 0.646 | NA | NA | NA | NA |
| rs635538 | Hand grip strength (left) | CAD | -0.024 | 0.016 | 0.003 | 0.017 | 1.00E-14 | 0.347 | NA | NA | NA | NA |
| rs66517261 | Hand grip strength (left) | CAD | -0.012 | -0.014 | 0.002 | 0.013 | 4.30E-09 | 0.248 | NA | NA | NA | NA |
| rs6754903 | Hand grip strength (left) | CAD | 0.016 | 0.009 | 0.002 | 0.012 | 2.15E-12 | 0.432 | NA | NA | NA | NA |
| rs694893 | Hand grip strength (left) | CAD | -0.011 | -0.005 | 0.002 | 0.010 | 2.17E-09 | 0.579 | NA | NA | NA | NA |
| rs6977081 | Hand grip strength (left) | CAD | 0.014 | 0.002 | 0.002 | 0.010 | 3.97E-14 | 0.822 | NA | NA | NA | NA |
| rs700518 | Hand grip strength (left) | CAD | 0.011 | 0.009 | 0.002 | 0.009 | 1.88E-09 | 0.361 | NA | NA | NA | NA |
| rs7196917 | Hand grip strength (left) | CAD | -0.013 | 0.012 | 0.002 | 0.009 | 1.38E-12 | 0.194 | NA | NA | NA | NA |
| rs7222242 | Hand grip strength (left) | CAD | -0.013 | -0.002 | 0.002 | 0.012 | 1.77E-09 | 0.874 | NA | NA | NA | NA |
| rs7571789 | Hand grip strength (left) | CAD | 0.013 | -0.008 | 0.002 | 0.009 | 2.79E-14 | 0.381 | NA | NA | NA | NA |
| rs7705189 | Hand grip strength (left) | CAD | 0.013 | -0.012 | 0.002 | 0.010 | 7.68E-14 | 0.230 | NA | NA | NA | NA |
| rs7740107 | Hand grip strength (left) | CAD | -0.017 | 0.030 | 0.002 | 0.011 | 2.95E-17 | 0.006 | NA | NA | NA | NA |
| rs7856625 | Hand grip strength (left) | CAD | -0.012 | -0.010 | 0.002 | 0.010 | 5.24E-12 | 0.300 | NA | NA | NA | NA |
| rs79172804 | Hand grip strength (left) | CAD | -0.016 | 0.040 | 0.002 | 0.014 | 2.44E-12 | 0.004 | TRUE | rs79172804 | rs62055869 | NA |
| rs7963801 | Hand grip strength (left) | CAD | -0.011 | 0.022 | 0.002 | 0.010 | 7.21E-10 | 0.032 | NA | NA | NA | NA |
| rs8108461 | Hand grip strength (left) | CAD | 0.010 | -0.025 | 0.002 | 0.010 | 1.16E-08 | 0.009 | NA | NA | NA | NA |
| rs823141 | Hand grip strength (left) | CAD | -0.013 | -0.019 | 0.002 | 0.010 | 6.27E-14 | 0.068 | NA | NA | NA | NA |
| rs867633 | Hand grip strength (left) | CAD | -0.010 | 0.020 | 0.002 | 0.010 | 3.98E-08 | 0.036 | NA | NA | NA | NA |
| rs934075 | Hand grip strength (left) | CAD | -0.011 | 0.005 | 0.002 | 0.010 | 1.09E-08 | 0.588 | NA | NA | NA | NA |
| rs9371881 | Hand grip strength (left) | CAD | 0.010 | -0.011 | 0.002 | 0.010 | 1.81E-08 | 0.289 | NA | NA | NA | NA |
| rs10210654 | Hand grip strength (right) | CAD | 0.013 | -0.001 | 0.002 | 0.012 | 2.05E-08 | 0.922 | NA | NA | NA | NA |
| rs1043515 | Hand grip strength (right) | CAD | 0.013 | -0.012 | 0.002 | 0.009 | 1.02E-13 | 0.190 | NA | NA | NA | NA |
| rs10798483 | Hand grip strength (right) | CAD | 0.016 | -0.003 | 0.002 | 0.009 | 1.84E-19 | 0.763 | NA | NA | NA | NA |
| rs10799428 | Hand grip strength (right) | CAD | -0.015 | -0.008 | 0.002 | 0.012 | 7.39E-11 | 0.506 | NA | NA | NA | NA |
| rs11236189 | Hand grip strength (right) | CAD | -0.017 | -0.001 | 0.002 | 0.009 | 4.72E-22 | 0.893 | NA | NA | NA | NA |
| rs11243202 | Hand grip strength (right) | CAD | 0.013 | -0.019 | 0.002 | 0.009 | 6.04E-13 | 0.042 | NA | NA | NA | NA |
| rs11669079 | Hand grip strength (right) | CAD | 0.013 | -0.011 | 0.002 | 0.011 | 7.11E-11 | 0.305 | NA | NA | NA | NA |
| rs11813532 | Hand grip strength (right) | CAD | -0.010 | 0.023 | 0.002 | 0.010 | 3.94E-08 | 0.021 | NA | NA | NA | NA |
| rs12127013 | Hand grip strength (right) | CAD | -0.015 | 0.025 | 0.003 | 0.014 | 1.27E-08 | 0.076 | NA | NA | NA | NA |
| rs12361415 | Hand grip strength (right) | CAD | 0.011 | -0.006 | 0.002 | 0.011 | 6.24E-09 | 0.596 | NA | NA | NA | NA |
| rs12452505 | Hand grip strength (right) | CAD | -0.014 | -0.001 | 0.003 | 0.015 | 1.61E-08 | 0.932 | NA | NA | NA | NA |
| rs1245463 | Hand grip strength (right) | CAD | 0.010 | 0.012 | 0.002 | 0.010 | 1.20E-08 | 0.222 | NA | NA | NA | NA |
| rs12562146 | Hand grip strength (right) | CAD | 0.014 | 0.000 | 0.003 | 0.013 | 8.79E-09 | 0.986 | NA | NA | NA | NA |
| rs12598856 | Hand grip strength (right) | CAD | 0.010 | -0.013 | 0.002 | 0.010 | 4.78E-08 | 0.199 | NA | NA | NA | NA |
| rs12708450 | Hand grip strength (right) | CAD | -0.013 | 0.001 | 0.002 | 0.011 | 3.17E-09 | 0.907 | NA | NA | NA | NA |
| rs12790261 | Hand grip strength (right) | CAD | -0.026 | 0.062 | 0.003 | 0.030 | 3.12E-16 | 0.041 | NA | NA | NA | NA |
| rs1280839 | Hand grip strength (right) | CAD | 0.010 | -0.007 | 0.002 | 0.010 | 4.65E-09 | 0.477 | NA | NA | NA | NA |
| rs12917449 | Hand grip strength (right) | CAD | -0.013 | 0.028 | 0.002 | 0.012 | 1.58E-09 | 0.021 | NA | NA | NA | NA |
| rs13017833 | Hand grip strength (right) | CAD | -0.027 | 0.020 | 0.005 | 0.028 | 1.18E-08 | 0.493 | NA | NA | NA | NA |
| rs13040292 | Hand grip strength (right) | CAD | 0.012 | -0.020 | 0.002 | 0.012 | 1.30E-09 | 0.095 | NA | NA | NA | NA |
| rs13107325 | Hand grip strength (right) | CAD | -0.031 | -0.007 | 0.003 | 0.022 | 3.07E-20 | 0.765 | NA | NA | NA | Body fat percentage |
| rs13150083 | Hand grip strength (right) | CAD | -0.011 | 0.002 | 0.002 | 0.011 | 2.60E-08 | 0.872 | NA | NA | NA | NA |
| rs143384 | Hand grip strength (right) | CAD | 0.023 | -0.015 | 0.002 | 0.010 | 2.75E-38 | 0.120 | NA | NA | NA | Body fat distribution |
| rs1442883 | Hand grip strength (right) | CAD | -0.011 | 0.013 | 0.002 | 0.012 | 3.16E-08 | 0.271 | NA | NA | NA | NA |
| rs150330307 | Hand grip strength (right) | CAD | -0.037 | -0.027 | 0.005 | 0.031 | 1.65E-13 | 0.389 | NA | NA | NA | NA |
| rs1514665 | Hand grip strength (right) | CAD | 0.010 | -0.009 | 0.002 | 0.009 | 1.37E-08 | 0.351 | NA | NA | NA | NA |
| rs1550115 | Hand grip strength (right) | CAD | 0.016 | -0.015 | 0.002 | 0.010 | 7.98E-16 | 0.150 | NA | NA | NA | NA |
| rs1556659 | Hand grip strength (right) | CAD | 0.017 | -0.034 | 0.002 | 0.010 | 1.76E-21 | 0.001 | NA | NA | NA | NA |
| rs17688916 | Hand grip strength (right) | CAD | -0.015 | 0.039 | 0.002 | 0.014 | 4.92E-11 | 0.006 | TRUE | rs17688916 | rs113661667 | NA |
| rs1991431 | Hand grip strength (right) | CAD | 0.011 | -0.019 | 0.002 | 0.010 | 2.50E-09 | 0.045 | NA | NA | NA | NA |
| rs200531 | Hand grip strength (right) | CAD | 0.013 | 0.021 | 0.002 | 0.012 | 3.86E-09 | 0.084 | NA | NA | NA | NA |
| rs2194411 | Hand grip strength (right) | CAD | 0.015 | -0.035 | 0.003 | 0.015 | 1.75E-08 | 0.017 | NA | NA | NA | NA |
| rs2265309 | Hand grip strength (right) | CAD | -0.010 | 0.027 | 0.002 | 0.009 | 7.55E-09 | 0.004 | NA | NA | NA | NA |
| rs2273555 | Hand grip strength (right) | CAD | 0.011 | 0.016 | 0.002 | 0.009 | 2.70E-10 | 0.082 | NA | NA | NA | NA |
| rs2431112 | Hand grip strength (right) | CAD | -0.011 | 0.003 | 0.002 | 0.009 | 2.20E-09 | 0.715 | NA | NA | NA | NA |
| rs249516 | Hand grip strength (right) | CAD | 0.011 | 0.012 | 0.002 | 0.009 | 2.91E-09 | 0.185 | NA | NA | NA | NA |
| rs2587505 | Hand grip strength (right) | CAD | -0.010 | 0.000 | 0.002 | 0.010 | 8.55E-09 | 0.977 | NA | NA | NA | NA |
| rs28417075 | Hand grip strength (right) | CAD | -0.020 | -0.032 | 0.003 | 0.039 | 1.35E-09 | 0.410 | TRUE | rs28417075 | rs71536555 | NA |
| rs2854152 | Hand grip strength (right) | CAD | 0.013 | -0.007 | 0.002 | 0.010 | 9.62E-13 | 0.496 | NA | NA | NA | NA |
| rs2871865 | Hand grip strength (right) | CAD | -0.026 | 0.053 | 0.003 | 0.015 | 1.37E-21 | 0.000 | NA | NA | NA | NA |
| rs2894602 | Hand grip strength (right) | CAD | 0.011 | -0.025 | 0.002 | 0.012 | 4.26E-08 | 0.036 | NA | NA | NA | NA |
| rs2971154 | Hand grip strength (right) | CAD | -0.011 | 0.003 | 0.002 | 0.010 | 1.90E-09 | 0.745 | NA | NA | NA | NA |
| rs3116605 | Hand grip strength (right) | CAD | -0.019 | 0.026 | 0.002 | 0.013 | 5.67E-19 | 0.038 | NA | NA | NA | NA |
| rs34217742 | Hand grip strength (right) | CAD | 0.016 | -0.001 | 0.003 | 0.016 | 4.10E-09 | 0.975 | NA | NA | NA | NA |
| rs34530577 | Hand grip strength (right) | CAD | -0.011 | 0.016 | 0.002 | 0.009 | 1.51E-09 | 0.084 | NA | NA | NA | NA |
| rs34588175 | Hand grip strength (right) | CAD | -0.022 | 0.019 | 0.002 | 0.012 | 3.39E-19 | 0.112 | NA | NA | NA | NA |
| rs34627176 | Hand grip strength (right) | CAD | -0.012 | 0.027 | 0.002 | 0.014 | 4.96E-08 | 0.057 | NA | NA | NA | NA |
| rs35910339 | Hand grip strength (right) | CAD | -0.013 | 0.003 | 0.002 | 0.011 | 1.58E-11 | 0.807 | NA | NA | NA | NA |
| rs3771498 | Hand grip strength (right) | CAD | 0.015 | -0.005 | 0.002 | 0.009 | 2.08E-17 | 0.582 | NA | NA | NA | NA |
| rs3773853 | Hand grip strength (right) | CAD | 0.010 | -0.011 | 0.002 | 0.009 | 2.25E-08 | 0.257 | NA | NA | NA | NA |
| rs3790076 | Hand grip strength (right) | CAD | -0.011 | 0.023 | 0.002 | 0.010 | 9.62E-11 | 0.015 | NA | NA | NA | NA |
| rs417591 | Hand grip strength (right) | CAD | 0.014 | -0.002 | 0.002 | 0.011 | 2.83E-11 | 0.864 | NA | NA | NA | NA |
| rs4308051 | Hand grip strength (right) | CAD | 0.016 | 0.004 | 0.002 | 0.012 | 2.91E-14 | 0.735 | NA | NA | NA | NA |
| rs4326984 | Hand grip strength (right) | CAD | -0.010 | -0.005 | 0.002 | 0.011 | 3.65E-08 | 0.678 | NA | NA | NA | NA |
| rs4373305 | Hand grip strength (right) | CAD | -0.011 | -0.001 | 0.002 | 0.010 | 4.74E-09 | 0.956 | NA | NA | NA | NA |
| rs4380799 | Hand grip strength (right) | CAD | -0.014 | 0.045 | 0.002 | 0.020 | 1.48E-12 | 0.028 | NA | NA | NA | NA |
| rs4594848 | Hand grip strength (right) | CAD | 0.014 | -0.004 | 0.002 | 0.010 | 2.45E-16 | 0.718 | NA | NA | NA | NA |
| rs4621706 | Hand grip strength (right) | CAD | -0.010 | -0.009 | 0.002 | 0.009 | 8.81E-09 | 0.330 | NA | NA | NA | NA |
| rs4730984 | Hand grip strength (right) | CAD | 0.012 | 0.005 | 0.002 | 0.011 | 1.01E-08 | 0.653 | NA | NA | NA | NA |
| rs4751671 | Hand grip strength (right) | CAD | 0.011 | -0.037 | 0.002 | 0.009 | 1.30E-09 | 0.000 | NA | NA | NA | NA |
| rs475390 | Hand grip strength (right) | CAD | -0.014 | -0.003 | 0.002 | 0.012 | 5.09E-11 | 0.771 | NA | NA | NA | NA |
| rs4764131 | Hand grip strength (right) | CAD | -0.017 | 0.014 | 0.002 | 0.010 | 4.35E-22 | 0.156 | NA | NA | NA | NA |
| rs4784329 | Hand grip strength (right) | CAD | -0.013 | -0.008 | 0.002 | 0.010 | 5.35E-14 | 0.395 | NA | NA | NA | NA |
| rs4785574 | Hand grip strength (right) | CAD | -0.011 | -0.020 | 0.002 | 0.009 | 4.54E-10 | 0.035 | NA | NA | NA | NA |
| rs4886778 | Hand grip strength (right) | CAD | 0.013 | 0.017 | 0.002 | 0.009 | 1.56E-13 | 0.071 | NA | NA | NA | NA |
| rs4927015 | Hand grip strength (right) | CAD | 0.013 | 0.000 | 0.002 | 0.010 | 4.92E-13 | 0.988 | NA | NA | NA | NA |
| rs4945185 | Hand grip strength (right) | CAD | -0.010 | -0.012 | 0.002 | 0.010 | 2.28E-08 | 0.207 | NA | NA | NA | NA |
| rs56412116 | Hand grip strength (right) | CAD | -0.012 | 0.034 | 0.002 | 0.012 | 1.32E-08 | 0.005 | NA | NA | NA | NA |
| rs57316347 | Hand grip strength (right) | CAD | -0.011 | -0.019 | 0.002 | 0.010 | 1.18E-08 | 0.055 | NA | NA | NA | NA |
| rs6006984 | Hand grip strength (right) | CAD | 0.011 | -0.002 | 0.002 | 0.010 | 9.67E-09 | 0.831 | NA | NA | NA | NA |
| rs635538 | Hand grip strength (right) | CAD | -0.024 | 0.016 | 0.003 | 0.017 | 1.88E-14 | 0.347 | NA | NA | NA | NA |
| rs6425501 | Hand grip strength (right) | CAD | 0.010 | 0.008 | 0.002 | 0.010 | 3.08E-08 | 0.408 | NA | NA | NA | NA |
| rs6539284 | Hand grip strength (right) | CAD | 0.012 | -0.018 | 0.002 | 0.010 | 1.17E-11 | 0.069 | NA | NA | NA | NA |
| rs6693965 | Hand grip strength (right) | CAD | -0.016 | -0.012 | 0.003 | 0.014 | 4.93E-10 | 0.397 | NA | NA | NA | NA |
| rs6977081 | Hand grip strength (right) | CAD | 0.013 | 0.002 | 0.002 | 0.010 | 1.39E-11 | 0.822 | NA | NA | NA | NA |
| rs700518 | Hand grip strength (right) | CAD | 0.011 | 0.009 | 0.002 | 0.009 | 2.91E-10 | 0.361 | NA | NA | NA | NA |
| rs7071654 | Hand grip strength (right) | CAD | 0.016 | 0.004 | 0.002 | 0.012 | 9.48E-11 | 0.739 | NA | NA | NA | NA |
| rs7206195 | Hand grip strength (right) | CAD | -0.018 | 0.012 | 0.002 | 0.015 | 2.60E-15 | 0.409 | NA | NA | NA | NA |
| rs721101 | Hand grip strength (right) | CAD | 0.012 | -0.007 | 0.002 | 0.011 | 4.60E-10 | 0.488 | NA | NA | NA | NA |
| rs7249081 | Hand grip strength (right) | CAD | 0.011 | -0.024 | 0.002 | 0.010 | 1.47E-09 | 0.014 | NA | NA | NA | NA |
| rs73307079 | Hand grip strength (right) | CAD | 0.014 | 0.010 | 0.002 | 0.011 | 4.55E-10 | 0.335 | NA | NA | NA | NA |
| rs75069534 | Hand grip strength (right) | CAD | 0.017 | 0.028 | 0.003 | 0.020 | 3.28E-09 | 0.165 | NA | NA | NA | NA |
| rs7740107 | Hand grip strength (right) | CAD | -0.017 | 0.030 | 0.002 | 0.011 | 8.50E-18 | 0.006 | NA | NA | NA | NA |
| rs7760564 | Hand grip strength (right) | CAD | 0.012 | -0.013 | 0.002 | 0.012 | 2.09E-08 | 0.264 | NA | NA | NA | NA |
| rs7871404 | Hand grip strength (right) | CAD | 0.012 | -0.011 | 0.002 | 0.013 | 2.70E-08 | 0.364 | NA | NA | NA | NA |
| rs7968902 | Hand grip strength (right) | CAD | -0.013 | 0.019 | 0.002 | 0.010 | 2.94E-12 | 0.050 | NA | NA | NA | NA |
| rs8012800 | Hand grip strength (right) | CAD | 0.011 | -0.010 | 0.002 | 0.011 | 4.32E-08 | 0.364 | NA | NA | NA | NA |
| rs8055199 | Hand grip strength (right) | CAD | -0.011 | -0.002 | 0.002 | 0.011 | 1.21E-09 | 0.874 | NA | NA | NA | NA |
| rs817316 | Hand grip strength (right) | CAD | 0.010 | 0.005 | 0.002 | 0.011 | 5.99E-09 | 0.631 | NA | NA | NA | NA |
| rs823130 | Hand grip strength (right) | CAD | -0.015 | -0.016 | 0.002 | 0.010 | 1.37E-16 | 0.110 | NA | NA | NA | NA |
| rs9322822 | Hand grip strength (right) | CAD | 0.012 | -0.012 | 0.002 | 0.010 | 3.74E-10 | 0.235 | NA | NA | NA | NA |
| rs934075 | Hand grip strength (right) | CAD | -0.011 | 0.005 | 0.002 | 0.010 | 5.54E-09 | 0.588 | NA | NA | NA | NA |
| rs9396861 | Hand grip strength (right) | CAD | -0.011 | 0.021 | 0.002 | 0.011 | 9.65E-10 | 0.044 | NA | NA | NA | NA |
| rs973767 | Hand grip strength (right) | CAD | 0.014 | 0.033 | 0.002 | 0.014 | 7.97E-09 | 0.018 | NA | NA | NA | NA |
| rs9847951 | Hand grip strength (right) | CAD | -0.010 | 0.013 | 0.002 | 0.009 | 2.32E-08 | 0.176 | NA | NA | NA | NA |

**CAD: coronary artery disease;**
