# Supplementary material for: Sarcopenia-related traits and coronary artery disease: a bi-directional Mendelian randomization study
Source: Aging (Albany NY). 2020 Feb 16;12(4):3340–53. doi: 10.18632/aging.102815 (PMC7066916; doi:10.18632/aging.102815)
Supplement: Supplementary Table 2 [file aging-12-102815-s001..docx]

**Supplementary Table 2. Independent IVs of body lean mass (㎏), handgrip strength (left, kg) and handgrip strength (right, kg) in stage 2 analysis.**

| SNP | exposure | outcome | beta.exposure | se.exposure | pval.exposure | beta.outcome | se.outcome | pval.outcome | proxy.outcome | target_snp.outcome | proxy_snp.outcome |  |
| --- | --- | --- | --- | --- | --- | --- | --- | --- | --- | --- | --- | --- |
| rs10080815 | CAD | body lean mass | -0.247 | 0.031 | 1.33E-15 | 0.004 | 0.005 | 0.444 | NA | NA | NA |  |
| rs10840293 | CAD | body lean mass | 0.055 | 0.010 | 1.28E-08 | 0.004 | 0.002 | 0.005 | NA | NA | NA |  |
| rs11065979 | CAD | body lean mass | -0.069 | 0.011 | 1.93E-10 | 0.015 | 0.002 | 0.000 | NA | NA | NA |  |
| rs11191416 | CAD | body lean mass | 0.079 | 0.014 | 4.65E-09 | -0.016 | 0.003 | 0.000 | NA | NA | NA |  |
| rs11556924 | CAD | body lean mass | 0.073 | 0.011 | 5.34E-11 | -0.002 | 0.002 | 0.165 | NA | NA | NA |  |
| rs115654617 | CAD | body lean mass | -0.138 | 0.016 | 3.12E-18 | -0.004 | 0.002 | 0.098 | NA | NA | NA |  |
| rs11838776 | CAD | body lean mass | -0.069 | 0.011 | 1.83E-10 | -0.004 | 0.002 | 0.015 | NA | NA | NA |  |
| rs1199338 | CAD | body lean mass | -0.074 | 0.012 | 3.90E-09 | 0.007 | 0.002 | 0.002 | NA | NA | NA |  |
| rs12202017 | CAD | body lean mass | 0.067 | 0.010 | 1.98E-11 | 0.003 | 0.002 | 0.079 | NA | NA | NA |  |
| rs1412444 | CAD | body lean mass | -0.067 | 0.010 | 5.15E-12 | 0.000 | 0.002 | 0.813 | NA | NA | NA |  |
| rs16986953 | CAD | body lean mass | -0.085 | 0.015 | 1.45E-08 | 0.001 | 0.003 | 0.722 | NA | NA | NA |  |
| rs17087335 | CAD | body lean mass | -0.061 | 0.011 | 4.59E-08 | -0.007 | 0.002 | 0.001 | NA | NA | NA |  |
| rs17678683 | CAD | body lean mass | -0.099 | 0.017 | 3.00E-09 | -0.005 | 0.003 | 0.070 | TRUE | rs17678683 | rs17740744 |  |
| rs180803 | CAD | body lean mass | -0.181 | 0.028 | 1.64E-10 | 0.000 | 0.007 | 0.955 | TRUE | rs180803 | rs5760293 |  |
| rs1870634 | CAD | body lean mass | 0.076 | 0.010 | 5.55E-15 | -0.002 | 0.002 | 0.350 | NA | NA | NA |  |
| rs2107595 | CAD | body lean mass | -0.073 | 0.011 | 8.05E-11 | 0.002 | 0.002 | 0.260 | NA | NA | NA |  |
| rs2128739 | CAD | body lean mass | -0.066 | 0.010 | 7.05E-11 | -0.003 | 0.002 | 0.073 | NA | NA | NA |  |
| rs2487928 | CAD | body lean mass | -0.063 | 0.010 | 4.41E-11 | 0.002 | 0.002 | 0.266 | NA | NA | NA |  |
| rs2519093 | CAD | body lean mass | -0.080 | 0.012 | 1.19E-11 | 0.004 | 0.002 | 0.057 | NA | NA | NA |  |
| rs2681472 | CAD | body lean mass | -0.074 | 0.011 | 6.17E-11 | -0.007 | 0.002 | 0.001 | NA | NA | NA |  |
| rs28451064 | CAD | body lean mass | -0.128 | 0.016 | 1.33E-15 | 0.003 | 0.002 | 0.164 | NA | NA | NA |  |
| rs2891168 | CAD | body lean mass | -0.193 | 0.009 | 2.29E-98 | 0.000 | 0.002 | 0.944 | NA | NA | NA |  |
| rs3918226 | CAD | body lean mass | -0.133 | 0.022 | 1.69E-09 | 0.022 | 0.003 | 0.000 | NA | NA | NA |  |
| rs4420638 | CAD | body lean mass | -0.092 | 0.014 | 7.07E-11 | 0.006 | 0.002 | 0.003 | NA | NA | NA |  |
| rs4468572 | CAD | body lean mass | 0.077 | 0.010 | 4.44E-16 | 0.000 | 0.002 | 0.844 | NA | NA | NA |  |
| rs4593108 | CAD | body lean mass | 0.071 | 0.012 | 8.82E-10 | 0.000 | 0.002 | 0.876 | NA | NA | NA |  |
| rs515135 | CAD | body lean mass | 0.067 | 0.012 | 3.09E-08 | -0.003 | 0.002 | 0.116 | NA | NA | NA |  |
| rs55730499 | CAD | body lean mass | -0.317 | 0.024 | 5.39E-39 | 0.001 | 0.003 | 0.824 | NA | NA | NA |  |
| rs56062135 | CAD | body lean mass | 0.070 | 0.012 | 4.52E-09 | 0.003 | 0.002 | 0.054 | NA | NA | NA |  |
| rs56289821 | CAD | body lean mass | 0.134 | 0.017 | 4.44E-15 | -0.011 | 0.002 | 0.000 | NA | NA | NA |  |
| rs56336142 | CAD | body lean mass | 0.067 | 0.012 | 1.85E-08 | -0.002 | 0.002 | 0.416 | NA | NA | NA |  |
| rs663129 | CAD | body lean mass | -0.058 | 0.011 | 3.20E-08 | -0.044 | 0.002 | 0.000 | NA | NA | NA |  |
| rs6689306 | CAD | body lean mass | -0.056 | 0.009 | 2.60E-09 | 0.002 | 0.002 | 0.120 | NA | NA | NA |  |
| rs67180937 | CAD | body lean mass | 0.079 | 0.011 | 1.01E-12 | 0.004 | 0.002 | 0.031 | NA | NA | NA |  |
| rs7212798 | CAD | body lean mass | -0.080 | 0.014 | 1.88E-08 | 0.002 | 0.002 | 0.273 | TRUE | rs7212798 | rs8080784 |  |
| rs7528419 | CAD | body lean mass | 0.115 | 0.011 | 1.97E-23 | -0.009 | 0.002 | 0.000 | NA | NA | NA |  |
| rs8042271 | CAD | body lean mass | -0.097 | 0.018 | 3.68E-08 | -0.001 | 0.004 | 0.817 | NA | NA | NA |  |
| rs9349379 | CAD | body lean mass | -0.132 | 0.010 | 1.81E-42 | 0.001 | 0.002 | 0.587 | NA | NA | NA |  |
| rs9970807 | CAD | body lean mass | 0.126 | 0.017 | 5.00E-14 | -0.002 | 0.003 | 0.452 | NA | NA | NA |  |
| rs10080815 | CAD | Hand grip strength (left) | -0.247 | 0.031 | 1.33E-15 | -0.001 | 0.006 | 0.869 | NA | NA | NA |  |
| rs10840293 | CAD | Hand grip strength (left) | 0.055 | 0.010 | 1.28E-08 | -0.001 | 0.002 | 0.675 | NA | NA | NA |  |
| rs11065979 | CAD | Hand grip strength (left) | -0.069 | 0.011 | 1.93E-10 | 0.004 | 0.002 | 0.029 | NA | NA | NA |  |
| rs11191416 | CAD | Hand grip strength (left) | 0.079 | 0.014 | 4.65E-09 | -0.007 | 0.003 | 0.027 | NA | NA | NA |  |
| rs11556924 | CAD | Hand grip strength (left) | 0.073 | 0.011 | 5.34E-11 | -0.004 | 0.002 | 0.037 | NA | NA | NA |  |
| rs115654617 | CAD | Hand grip strength (left) | -0.138 | 0.016 | 3.12E-18 | -0.001 | 0.003 | 0.731 | NA | NA | NA |  |
| rs11838776 | CAD | Hand grip strength (left) | -0.069 | 0.011 | 1.83E-10 | 0.000 | 0.002 | 0.843 | NA | NA | NA |  |
| rs1199338 | CAD | Hand grip strength (left) | -0.074 | 0.012 | 3.90E-09 | 0.003 | 0.002 | 0.181 | NA | NA | NA |  |
| rs12202017 | CAD | Hand grip strength (left) | 0.067 | 0.010 | 1.98E-11 | 0.002 | 0.002 | 0.257 | NA | NA | NA |  |
| rs1412444 | CAD | Hand grip strength (left) | -0.067 | 0.010 | 5.15E-12 | 0.000 | 0.002 | 0.819 | NA | NA | NA |  |
| rs16986953 | CAD | Hand grip strength (left) | -0.085 | 0.015 | 1.45E-08 | 0.001 | 0.003 | 0.836 | NA | NA | NA |  |
| rs17087335 | CAD | Hand grip strength (left) | -0.061 | 0.011 | 4.59E-08 | -0.003 | 0.002 | 0.236 | NA | NA | NA |  |
| rs17678683 | CAD | Hand grip strength (left) | -0.099 | 0.017 | 3.00E-09 | 0.000 | 0.003 | 0.950 | TRUE | rs17678683 | rs17740744 |  |
| rs180803 | CAD | Hand grip strength (left) | -0.181 | 0.028 | 1.64E-10 | 0.001 | 0.008 | 0.934 | TRUE | rs180803 | rs5760293 |  |
| rs1870634 | CAD | Hand grip strength (left) | 0.076 | 0.010 | 5.55E-15 | 0.001 | 0.002 | 0.480 | NA | NA | NA |  |
| rs2107595 | CAD | Hand grip strength (left) | -0.073 | 0.011 | 8.05E-11 | 0.002 | 0.002 | 0.317 | NA | NA | NA |  |
| rs2128739 | CAD | Hand grip strength (left) | -0.066 | 0.010 | 7.05E-11 | -0.001 | 0.002 | 0.482 | NA | NA | NA |  |
| rs2487928 | CAD | Hand grip strength (left) | -0.063 | 0.010 | 4.41E-11 | 0.003 | 0.002 | 0.057 | NA | NA | NA |  |
| rs2519093 | CAD | Hand grip strength (left) | -0.080 | 0.012 | 1.19E-11 | 0.010 | 0.002 | 0.000 | NA | NA | NA |  |
| rs2681472 | CAD | Hand grip strength (left) | -0.074 | 0.011 | 6.17E-11 | 0.001 | 0.002 | 0.806 | NA | NA | NA |  |
| rs28451064 | CAD | Hand grip strength (left) | -0.128 | 0.016 | 1.33E-15 | 0.001 | 0.003 | 0.833 | NA | NA | NA |  |
| rs2891168 | CAD | Hand grip strength (left) | -0.193 | 0.009 | 2.29E-98 | 0.001 | 0.002 | 0.561 | NA | NA | NA |  |
| rs3918226 | CAD | Hand grip strength (left) | -0.133 | 0.022 | 1.69E-09 | 0.000 | 0.003 | 0.977 | NA | NA | NA |  |
| rs4420638 | CAD | Hand grip strength (left) | -0.092 | 0.014 | 7.07E-11 | -0.002 | 0.002 | 0.304 | NA | NA | NA |  |
| rs4468572 | CAD | Hand grip strength (left) | 0.077 | 0.010 | 4.44E-16 | 0.001 | 0.002 | 0.537 | NA | NA | NA |  |
| rs4593108 | CAD | Hand grip strength (left) | 0.071 | 0.012 | 8.82E-10 | -0.002 | 0.002 | 0.359 | NA | NA | NA |  |
| rs515135 | CAD | Hand grip strength (left) | 0.067 | 0.012 | 3.09E-08 | 0.003 | 0.002 | 0.157 | NA | NA | NA |  |
| rs55730499 | CAD | Hand grip strength (left) | -0.317 | 0.024 | 5.39E-39 | 0.004 | 0.003 | 0.181 | NA | NA | NA |  |
| rs56062135 | CAD | Hand grip strength (left) | 0.070 | 0.012 | 4.52E-09 | 0.004 | 0.002 | 0.087 | NA | NA | NA |  |
| rs56289821 | CAD | Hand grip strength (left) | 0.134 | 0.017 | 4.44E-15 | -0.002 | 0.003 | 0.417 | NA | NA | NA |  |
| rs56336142 | CAD | Hand grip strength (left) | 0.067 | 0.012 | 1.85E-08 | -0.001 | 0.002 | 0.548 | NA | NA | NA |  |
| rs663129 | CAD | Hand grip strength (left) | -0.058 | 0.011 | 3.20E-08 | -0.009 | 0.002 | 0.000 | NA | NA | NA |  |
| rs6689306 | CAD | Hand grip strength (left) | -0.056 | 0.009 | 2.60E-09 | 0.003 | 0.002 | 0.057 | NA | NA | NA |  |
| rs67180937 | CAD | Hand grip strength (left) | 0.079 | 0.011 | 1.01E-12 | 0.001 | 0.002 | 0.804 | NA | NA | NA |  |
| rs7212798 | CAD | Hand grip strength (left) | -0.080 | 0.014 | 1.88E-08 | -0.002 | 0.002 | 0.543 | TRUE | rs7212798 | rs8080784 |  |
| rs7528419 | CAD | Hand grip strength (left) | 0.115 | 0.011 | 1.97E-23 | -0.006 | 0.002 | 0.009 | NA | NA | NA |  |
| rs8042271 | CAD | Hand grip strength (left) | -0.097 | 0.018 | 3.68E-08 | 0.001 | 0.005 | 0.836 | NA | NA | NA |  |
| rs9349379 | CAD | Hand grip strength (left) | -0.132 | 0.010 | 1.81E-42 | -0.009 | 0.002 | 0.000 | NA | NA | NA |  |
| rs9970807 | CAD | Hand grip strength (left) | 0.126 | 0.017 | 5.00E-14 | 0.000 | 0.003 | 0.888 | NA | NA | NA |  |
| rs10080815 | CAD | Hand grip strength (right) | -0.247 | 0.031 | 1.33E-15 | -0.001 | 0.006 | 0.842 | NA | NA | NA |  |
| rs10840293 | CAD | Hand grip strength (right) | 0.055 | 0.010 | 1.28E-08 | -0.001 | 0.002 | 0.541 | NA | NA | NA |  |
| rs11065979 | CAD | Hand grip strength (right) | -0.069 | 0.011 | 1.93E-10 | 0.003 | 0.002 | 0.093 | NA | NA | NA |  |
| rs11191416 | CAD | Hand grip strength (right) | 0.079 | 0.014 | 4.65E-09 | -0.006 | 0.003 | 0.045 | NA | NA | NA |  |
| rs11556924 | CAD | Hand grip strength (right) | 0.073 | 0.011 | 5.34E-11 | -0.004 | 0.002 | 0.034 | NA | NA | NA |  |
| rs115654617 | CAD | Hand grip strength (right) | -0.138 | 0.016 | 3.12E-18 | -0.002 | 0.003 | 0.448 | NA | NA | NA |  |
| rs11838776 | CAD | Hand grip strength (right) | -0.069 | 0.011 | 1.83E-10 | 0.000 | 0.002 | 0.822 | NA | NA | NA |  |
| rs1199338 | CAD | Hand grip strength (right) | -0.074 | 0.012 | 3.90E-09 | 0.005 | 0.002 | 0.037 | NA | NA | NA |  |
| rs12202017 | CAD | Hand grip strength (right) | 0.067 | 0.010 | 1.98E-11 | 0.000 | 0.002 | 0.948 | NA | NA | NA |  |
| rs1412444 | CAD | Hand grip strength (right) | -0.067 | 0.010 | 5.15E-12 | 0.002 | 0.002 | 0.267 | NA | NA | NA |  |
| rs16986953 | CAD | Hand grip strength (right) | -0.085 | 0.015 | 1.45E-08 | 0.002 | 0.004 | 0.528 | NA | NA | NA |  |
| rs17087335 | CAD | Hand grip strength (right) | -0.061 | 0.011 | 4.59E-08 | -0.001 | 0.002 | 0.697 | NA | NA | NA |  |
| rs17678683 | CAD | Hand grip strength (right) | -0.099 | 0.017 | 3.00E-09 | -0.001 | 0.003 | 0.867 | TRUE | rs17678683 | rs17740744 |  |
| rs180803 | CAD | Hand grip strength (right) | -0.181 | 0.028 | 1.64E-10 | 0.002 | 0.008 | 0.856 | TRUE | rs180803 | rs5760293 |  |
| rs1870634 | CAD | Hand grip strength (right) | 0.076 | 0.010 | 5.55E-15 | 0.001 | 0.002 | 0.546 | NA | NA | NA |  |
| rs2107595 | CAD | Hand grip strength (right) | -0.073 | 0.011 | 8.05E-11 | 0.001 | 0.002 | 0.575 | NA | NA | NA |  |
| rs2128739 | CAD | Hand grip strength (right) | -0.066 | 0.010 | 7.05E-11 | 0.000 | 0.002 | 0.897 | NA | NA | NA |  |
| rs2487928 | CAD | Hand grip strength (right) | -0.063 | 0.010 | 4.41E-11 | 0.001 | 0.002 | 0.453 | NA | NA | NA |  |
| rs2519093 | CAD | Hand grip strength (right) | -0.080 | 0.012 | 1.19E-11 | 0.010 | 0.002 | 0.000 | NA | NA | NA |  |
| rs2681472 | CAD | Hand grip strength (right) | -0.074 | 0.011 | 6.17E-11 | 0.001 | 0.002 | 0.607 | NA | NA | NA |  |
| rs28451064 | CAD | Hand grip strength (right) | -0.128 | 0.016 | 1.33E-15 | 0.000 | 0.003 | 0.852 | NA | NA | NA |  |
| rs2891168 | CAD | Hand grip strength (right) | -0.193 | 0.009 | 2.29E-98 | -0.001 | 0.002 | 0.774 | NA | NA | NA |  |
| rs3918226 | CAD | Hand grip strength (right) | -0.133 | 0.022 | 1.69E-09 | 0.001 | 0.003 | 0.845 | NA | NA | NA |  |
| rs4420638 | CAD | Hand grip strength (right) | -0.092 | 0.014 | 7.07E-11 | -0.005 | 0.002 | 0.028 | NA | NA | NA |  |
| rs4468572 | CAD | Hand grip strength (right) | 0.077 | 0.010 | 4.44E-16 | 0.002 | 0.002 | 0.270 | NA | NA | NA |  |
| rs4593108 | CAD | Hand grip strength (right) | 0.071 | 0.012 | 8.82E-10 | -0.002 | 0.002 | 0.361 | NA | NA | NA |  |
| rs515135 | CAD | Hand grip strength (right) | 0.067 | 0.012 | 3.09E-08 | 0.003 | 0.002 | 0.273 | NA | NA | NA |  |
| rs55730499 | CAD | Hand grip strength (right) | -0.317 | 0.024 | 5.39E-39 | 0.005 | 0.003 | 0.132 | NA | NA | NA |  |
| rs56062135 | CAD | Hand grip strength (right) | 0.070 | 0.012 | 4.52E-09 | 0.003 | 0.002 | 0.149 | NA | NA | NA |  |
| rs56289821 | CAD | Hand grip strength (right) | 0.134 | 0.017 | 4.44E-15 | -0.004 | 0.003 | 0.142 | NA | NA | NA |  |
| rs56336142 | CAD | Hand grip strength (right) | 0.067 | 0.012 | 1.85E-08 | -0.001 | 0.002 | 0.488 | NA | NA | NA |  |
| rs663129 | CAD | Hand grip strength (right) | -0.058 | 0.011 | 3.20E-08 | -0.009 | 0.002 | 0.000 | NA | NA | NA |  |
| rs6689306 | CAD | Hand grip strength (right) | -0.056 | 0.009 | 2.60E-09 | 0.003 | 0.002 | 0.078 | NA | NA | NA |  |
| rs67180937 | CAD | Hand grip strength (right) | 0.079 | 0.011 | 1.01E-12 | 0.000 | 0.002 | 0.988 | NA | NA | NA |  |
| rs7212798 | CAD | Hand grip strength (right) | -0.080 | 0.014 | 1.88E-08 | -0.002 | 0.002 | 0.472 | TRUE | rs7212798 | rs8080784 |  |
| rs7528419 | CAD | Hand grip strength (right) | 0.115 | 0.011 | 1.97E-23 | -0.006 | 0.002 | 0.003 | NA | NA | NA |  |
| rs8042271 | CAD | Hand grip strength (right) | -0.097 | 0.018 | 3.68E-08 | -0.001 | 0.005 | 0.894 | NA | NA | NA |  |
| rs9349379 | CAD | Hand grip strength (right) | -0.132 | 0.010 | 1.81E-42 | -0.007 | 0.002 | 0.000 | NA | NA | NA |  |
| rs9970807 | CAD | Hand grip strength (right) | 0.126 | 0.017 | 5.00E-14 | -0.002 | 0.003 | 0.534 | NA | NA | NA |  |

**CAD: coronary artery disease;**
