# Supplementary material for: Sarcopenia-related traits and coronary artery disease: a bi-directional Mendelian randomization study
Source: Aging (Albany NY). 2020 Feb 16;12(4):3340–53. doi: 10.18632/aging.102815 (PMC7066916; doi:10.18632/aging.102815)
Supplement: Supplementary Table 4 [file aging-12-102815-s002..docx]

**Supplementary Table 4. Independent IVs of body lean mass (㎏), handgrip strength (left, kg) and handgrip strength (right, kg) and coronary artery disease in negative control analysis (myopia).**

| SNP | exposure | outcome | beta.exposure | se.exposure | pval.exposure | beta.outcome | se.outcome | pval.outcome | proxy.outcome | target_snp.outcome | proxy_snp.outcome |
| --- | --- | --- | --- | --- | --- | --- | --- | --- | --- | --- | --- |
| rs10080815 | CAD | myopia | -0.247 | 0.031 | 1.33E-15 | 0.001 | 0.002 | 0.757 | NA | NA | NA |
| rs10840293 | CAD | myopia | 0.055 | 0.010 | 1.28E-08 | -0.001 | 0.001 | 0.065 | NA | NA | NA |
| rs11065979 | CAD | myopia | -0.069 | 0.011 | 1.93E-10 | -0.002 | 0.001 | 0.005 | NA | NA | NA |
| rs11191416 | CAD | myopia | 0.079 | 0.014 | 4.65E-09 | 0.000 | 0.001 | 0.870 | NA | NA | NA |
| rs11556924 | CAD | myopia | 0.073 | 0.011 | 5.34E-11 | 0.002 | 0.001 | 0.013 | NA | NA | NA |
| rs115654617 | CAD | myopia | -0.138 | 0.016 | 3.12E-18 | 0.000 | 0.001 | 0.829 | NA | NA | NA |
| rs11838776 | CAD | myopia | -0.069 | 0.011 | 1.83E-10 | -0.001 | 0.001 | 0.193 | NA | NA | NA |
| rs1199338 | CAD | myopia | -0.074 | 0.012 | 3.90E-09 | -0.002 | 0.001 | 0.041 | NA | NA | NA |
| rs12202017 | CAD | myopia | 0.067 | 0.010 | 1.98E-11 | 0.000 | 0.001 | 0.861 | NA | NA | NA |
| rs1412444 | CAD | myopia | -0.067 | 0.010 | 5.15E-12 | -0.001 | 0.001 | 0.332 | NA | NA | NA |
| rs16986953 | CAD | myopia | -0.085 | 0.015 | 1.45E-08 | 0.002 | 0.001 | 0.097 | NA | NA | NA |
| rs17087335 | CAD | myopia | -0.061 | 0.011 | 4.59E-08 | -0.001 | 0.001 | 0.419 | NA | NA | NA |
| rs17678683 | CAD | myopia | -0.099 | 0.017 | 3.00E-09 | -0.001 | 0.001 | 0.360 | TRUE | rs17678683 | rs17740744 |
| rs180803 | CAD | myopia | -0.181 | 0.028 | 1.64E-10 | -0.002 | 0.003 | 0.479 | TRUE | rs180803 | rs5760293 |
| rs1870634 | CAD | myopia | 0.076 | 0.010 | 5.55E-15 | 0.001 | 0.001 | 0.095 | NA | NA | NA |
| rs2107595 | CAD | myopia | -0.073 | 0.011 | 8.05E-11 | 0.000 | 0.001 | 0.667 | NA | NA | NA |
| rs2128739 | CAD | myopia | -0.066 | 0.010 | 7.05E-11 | -0.001 | 0.001 | 0.175 | NA | NA | NA |
| rs2487928 | CAD | myopia | -0.063 | 0.010 | 4.41E-11 | 0.001 | 0.001 | 0.039 | NA | NA | NA |
| rs2519093 | CAD | myopia | -0.080 | 0.012 | 1.19E-11 | 0.001 | 0.001 | 0.413 | NA | NA | NA |
| rs2681472 | CAD | myopia | -0.074 | 0.011 | 6.17E-11 | 0.000 | 0.001 | 0.881 | NA | NA | NA |
| rs28451064 | CAD | myopia | -0.128 | 0.016 | 1.33E-15 | -0.003 | 0.001 | 0.006 | NA | NA | NA |
| rs2891168 | CAD | myopia | -0.193 | 0.009 | 2.29E-98 | 0.001 | 0.001 | 0.439 | NA | NA | NA |
| rs3918226 | CAD | myopia | -0.133 | 0.022 | 1.69E-09 | 0.001 | 0.001 | 0.392 | NA | NA | NA |
| rs4420638 | CAD | myopia | -0.092 | 0.014 | 7.07E-11 | 0.002 | 0.001 | 0.004 | NA | NA | NA |
| rs4468572 | CAD | myopia | 0.077 | 0.010 | 4.44E-16 | 0.001 | 0.001 | 0.031 | NA | NA | NA |
| rs4593108 | CAD | myopia | 0.071 | 0.012 | 8.82E-10 | 0.002 | 0.001 | 0.023 | NA | NA | NA |
| rs515135 | CAD | myopia | 0.067 | 0.012 | 3.09E-08 | 0.000 | 0.001 | 0.829 | NA | NA | NA |
| rs55730499 | CAD | myopia | -0.317 | 0.024 | 5.39E-39 | 0.001 | 0.001 | 0.318 | NA | NA | NA |
| rs56062135 | CAD | myopia | 0.070 | 0.012 | 4.52E-09 | 0.000 | 0.001 | 0.631 | NA | NA | NA |
| rs56289821 | CAD | myopia | 0.134 | 0.017 | 4.44E-15 | -0.001 | 0.001 | 0.242 | NA | NA | NA |
| rs56336142 | CAD | myopia | 0.067 | 0.012 | 1.85E-08 | -0.001 | 0.001 | 0.235 | NA | NA | NA |
| rs663129 | CAD | myopia | -0.058 | 0.011 | 3.20E-08 | 0.001 | 0.001 | 0.179 | NA | NA | NA |
| rs6689306 | CAD | myopia | -0.056 | 0.009 | 2.60E-09 | 0.000 | 0.001 | 0.748 | NA | NA | NA |
| rs67180937 | CAD | myopia | 0.079 | 0.011 | 1.01E-12 | 0.000 | 0.001 | 0.629 | NA | NA | NA |
| rs7212798 | CAD | myopia | -0.080 | 0.014 | 1.88E-08 | 0.001 | 0.001 | 0.377 | TRUE | rs7212798 | rs8080784 |
| rs7528419 | CAD | myopia | 0.115 | 0.011 | 1.97E-23 | 0.000 | 0.001 | 0.530 | NA | NA | NA |
| rs8042271 | CAD | myopia | -0.097 | 0.018 | 3.68E-08 | -0.001 | 0.002 | 0.638 | NA | NA | NA |
| rs9349379 | CAD | myopia | -0.132 | 0.010 | 1.81E-42 | 0.000 | 0.001 | 0.814 | NA | NA | NA |
| rs9970807 | CAD | myopia | 0.126 | 0.017 | 5.00E-14 | 0.001 | 0.001 | 0.518 | NA | NA | NA |
| rs10203320 | body lean mass | myopia | 0.012 | 0.002 | 3.08E-13 | 0.001 | 0.001 | 0.428 | NA | NA | NA |
| rs1022523 | body lean mass | myopia | 0.014 | 0.002 | 1.44E-16 | -0.001 | 0.001 | 0.449 | NA | NA | NA |
| rs10236214 | body lean mass | myopia | 0.020 | 0.002 | 1.12E-36 | 0.001 | 0.001 | 0.444 | NA | NA | NA |
| rs10237306 | body lean mass | myopia | 0.011 | 0.002 | 1.01E-12 | 0.000 | 0.001 | 0.869 | NA | NA | NA |
| rs10257870 | body lean mass | myopia | 0.016 | 0.003 | 4.59E-08 | 0.000 | 0.001 | 0.910 | NA | NA | NA |
| rs10260993 | body lean mass | myopia | -0.012 | 0.002 | 3.39E-10 | 0.000 | 0.001 | 0.588 | NA | NA | NA |
| rs1043413 | body lean mass | myopia | 0.010 | 0.002 | 9.60E-10 | 0.002 | 0.001 | 0.015 | NA | NA | NA |
| rs1047891 | body lean mass | myopia | 0.017 | 0.002 | 1.51E-25 | 0.000 | 0.001 | 0.679 | NA | NA | NA |
| rs10483727 | body lean mass | myopia | -0.022 | 0.002 | 1.46E-43 | -0.002 | 0.001 | 0.000 | NA | NA | NA |
| rs10485622 | body lean mass | myopia | -0.019 | 0.002 | 9.84E-22 | -0.001 | 0.001 | 0.292 | NA | NA | NA |
| rs1064213 | body lean mass | myopia | 0.012 | 0.002 | 5.88E-14 | 0.002 | 0.001 | 0.007 | NA | NA | NA |
| rs10746837 | body lean mass | myopia | -0.010 | 0.002 | 3.06E-11 | 0.002 | 0.001 | 0.016 | NA | NA | NA |
| rs10748128 | body lean mass | myopia | 0.012 | 0.002 | 3.47E-13 | 0.001 | 0.001 | 0.057 | NA | NA | NA |
| rs10767735 | body lean mass | myopia | -0.009 | 0.002 | 4.17E-09 | 0.002 | 0.001 | 0.001 | NA | NA | NA |
| rs10770705 | body lean mass | myopia | -0.009 | 0.002 | 9.40E-09 | 0.001 | 0.001 | 0.441 | NA | NA | NA |
| rs10775348 | body lean mass | myopia | 0.014 | 0.002 | 4.41E-16 | 0.001 | 0.001 | 0.173 | NA | NA | NA |
| rs10798945 | body lean mass | myopia | 0.012 | 0.002 | 7.47E-11 | -0.001 | 0.001 | 0.060 | NA | NA | NA |
| rs10843139 | body lean mass | myopia | -0.013 | 0.002 | 4.07E-15 | 0.002 | 0.001 | 0.007 | NA | NA | NA |
| rs10847415 | body lean mass | myopia | 0.015 | 0.002 | 9.38E-19 | 0.000 | 0.001 | 0.505 | NA | NA | NA |
| rs10931008 | body lean mass | myopia | -0.013 | 0.002 | 5.11E-15 | 0.000 | 0.001 | 0.870 | NA | NA | NA |
| rs10938398 | body lean mass | myopia | 0.011 | 0.002 | 5.98E-13 | 0.001 | 0.001 | 0.086 | NA | NA | NA |
| rs10953112 | body lean mass | myopia | 0.015 | 0.002 | 2.17E-14 | -0.001 | 0.001 | 0.264 | NA | NA | NA |
| rs10958683 | body lean mass | myopia | -0.010 | 0.002 | 3.05E-08 | 0.001 | 0.001 | 0.064 | NA | NA | NA |
| rs10982888 | body lean mass | myopia | -0.016 | 0.002 | 5.40E-11 | 0.001 | 0.001 | 0.320 | NA | NA | NA |
| rs10990621 | body lean mass | myopia | 0.018 | 0.002 | 9.51E-18 | 0.001 | 0.001 | 0.397 | NA | NA | NA |
| rs10995366 | body lean mass | myopia | -0.011 | 0.002 | 2.73E-10 | -0.001 | 0.001 | 0.458 | NA | NA | NA |
| rs11013045 | body lean mass | myopia | -0.009 | 0.002 | 6.27E-09 | -0.001 | 0.001 | 0.235 | NA | NA | NA |
| rs11014285 | body lean mass | myopia | 0.018 | 0.002 | 9.35E-18 | -0.001 | 0.001 | 0.549 | NA | NA | NA |
| rs11023199 | body lean mass | myopia | -0.009 | 0.002 | 1.27E-08 | -0.002 | 0.001 | 0.002 | NA | NA | NA |
| rs11030119 | body lean mass | myopia | 0.017 | 0.002 | 1.04E-23 | 0.000 | 0.001 | 0.581 | NA | NA | NA |
| rs11065015 | body lean mass | myopia | -0.031 | 0.005 | 6.97E-11 | -0.001 | 0.002 | 0.726 | NA | NA | NA |
| rs11065979 | body lean mass | myopia | -0.015 | 0.002 | 6.59E-22 | 0.002 | 0.001 | 0.005 | NA | NA | NA |
| rs11098675 | body lean mass | myopia | -0.016 | 0.002 | 6.61E-16 | 0.001 | 0.001 | 0.354 | NA | NA | NA |
| rs1111818 | body lean mass | myopia | 0.009 | 0.002 | 3.44E-09 | 0.001 | 0.001 | 0.466 | NA | NA | NA |
| rs11125074 | body lean mass | myopia | 0.009 | 0.002 | 2.69E-08 | -0.001 | 0.001 | 0.063 | NA | NA | NA |
| rs11158820 | body lean mass | myopia | -0.010 | 0.002 | 2.45E-09 | -0.001 | 0.001 | 0.064 | NA | NA | NA |
| rs111632154 | body lean mass | myopia | -0.030 | 0.004 | 4.73E-17 | -0.001 | 0.002 | 0.395 | NA | NA | NA |
| rs111640872 | body lean mass | myopia | 0.015 | 0.002 | 3.20E-20 | 0.002 | 0.001 | 0.028 | NA | NA | NA |
| rs111901479 | body lean mass | myopia | 0.024 | 0.003 | 3.67E-19 | 0.001 | 0.001 | 0.605 | NA | NA | NA |
| rs111964059 | body lean mass | myopia | 0.016 | 0.003 | 1.05E-08 | -0.001 | 0.001 | 0.609 | NA | NA | NA |
| rs11205617 | body lean mass | myopia | 0.011 | 0.002 | 4.82E-11 | 0.002 | 0.001 | 0.001 | NA | NA | NA |
| rs11217863 | body lean mass | myopia | -0.016 | 0.002 | 6.36E-11 | 0.000 | 0.001 | 0.913 | NA | NA | NA |
| rs11240565 | body lean mass | myopia | 0.014 | 0.002 | 3.09E-19 | 0.000 | 0.001 | 0.575 | NA | NA | NA |
| rs11245450 | body lean mass | myopia | -0.010 | 0.002 | 1.20E-10 | 0.000 | 0.001 | 0.770 | NA | NA | NA |
| rs112957890 | body lean mass | myopia | 0.012 | 0.002 | 1.24E-11 | 0.001 | 0.001 | 0.094 | NA | NA | NA |
| rs113457986 | body lean mass | myopia | 0.040 | 0.006 | 7.63E-10 | 0.001 | 0.003 | 0.735 | NA | NA | NA |
| rs113902512 | body lean mass | myopia | 0.011 | 0.002 | 4.80E-08 | 0.001 | 0.001 | 0.366 | NA | NA | NA |
| rs11545482 | body lean mass | myopia | -0.035 | 0.005 | 8.76E-11 | -0.002 | 0.002 | 0.456 | NA | NA | NA |
| rs115574684 | body lean mass | myopia | 0.022 | 0.004 | 2.84E-08 | 0.002 | 0.002 | 0.229 | NA | NA | NA |
| rs11580196 | body lean mass | myopia | -0.012 | 0.002 | 2.03E-09 | 0.000 | 0.001 | 0.884 | NA | NA | NA |
| rs115912456 | body lean mass | myopia | 0.025 | 0.004 | 9.03E-11 | -0.003 | 0.002 | 0.083 | NA | NA | NA |
| rs11594322 | body lean mass | myopia | -0.017 | 0.002 | 1.23E-13 | -0.002 | 0.001 | 0.121 | NA | NA | NA |
| rs116072427 | body lean mass | myopia | -0.025 | 0.003 | 9.68E-17 | 0.000 | 0.001 | 0.998 | NA | NA | NA |
| rs116165844 | body lean mass | myopia | -0.014 | 0.002 | 8.10E-10 | -0.001 | 0.001 | 0.402 | NA | NA | NA |
| rs11655578 | body lean mass | myopia | 0.010 | 0.002 | 4.36E-08 | -0.001 | 0.001 | 0.075 | NA | NA | NA |
| rs11657325 | body lean mass | myopia | -0.016 | 0.002 | 4.02E-20 | -0.002 | 0.001 | 0.023 | NA | NA | NA |
| rs11658134 | body lean mass | myopia | -0.011 | 0.002 | 6.71E-13 | -0.001 | 0.001 | 0.178 | NA | NA | NA |
| rs11667280 | body lean mass | myopia | -0.011 | 0.002 | 4.69E-09 | -0.001 | 0.001 | 0.397 | NA | NA | NA |
| rs11678716 | body lean mass | myopia | -0.022 | 0.003 | 1.58E-12 | -0.003 | 0.001 | 0.014 | NA | NA | NA |
| rs11689727 | body lean mass | myopia | -0.011 | 0.002 | 4.83E-12 | 0.000 | 0.001 | 0.705 | NA | NA | NA |
| rs11717749 | body lean mass | myopia | 0.016 | 0.002 | 2.20E-11 | -0.001 | 0.001 | 0.364 | NA | NA | NA |
| rs117543413 | body lean mass | myopia | -0.040 | 0.006 | 5.71E-12 | -0.001 | 0.003 | 0.812 | NA | NA | NA |
| rs11783086 | body lean mass | myopia | 0.009 | 0.002 | 1.69E-08 | 0.000 | 0.001 | 0.606 | NA | NA | NA |
| rs11803905 | body lean mass | myopia | 0.010 | 0.002 | 8.66E-09 | 0.000 | 0.001 | 0.832 | NA | NA | NA |
| rs11855017 | body lean mass | myopia | 0.013 | 0.002 | 8.34E-11 | -0.001 | 0.001 | 0.363 | NA | NA | NA |
| rs11880992 | body lean mass | myopia | 0.015 | 0.002 | 5.83E-21 | 0.000 | 0.001 | 0.629 | NA | NA | NA |
| rs11951673 | body lean mass | myopia | -0.009 | 0.002 | 2.86E-08 | 0.000 | 0.001 | 0.569 | NA | NA | NA |
| rs12041740 | body lean mass | myopia | -0.015 | 0.002 | 3.93E-18 | -0.001 | 0.001 | 0.347 | NA | NA | NA |
| rs12072845 | body lean mass | myopia | -0.015 | 0.002 | 2.70E-20 | 0.000 | 0.001 | 0.770 | NA | NA | NA |
| rs12073468 | body lean mass | myopia | 0.012 | 0.002 | 2.55E-10 | 0.001 | 0.001 | 0.419 | NA | NA | NA |
| rs12140153 | body lean mass | myopia | -0.016 | 0.003 | 1.83E-09 | 0.000 | 0.001 | 0.848 | NA | NA | NA |
| rs12193797 | body lean mass | myopia | -0.021 | 0.002 | 3.45E-21 | 0.000 | 0.001 | 0.803 | NA | NA | NA |
| rs12209223 | body lean mass | myopia | 0.018 | 0.003 | 4.92E-12 | 0.000 | 0.001 | 0.917 | NA | NA | NA |
| rs12216497 | body lean mass | myopia | -0.011 | 0.002 | 4.31E-12 | 0.000 | 0.001 | 0.998 | NA | NA | NA |
| rs1222219 | body lean mass | myopia | -0.011 | 0.002 | 1.89E-09 | -0.002 | 0.001 | 0.032 | NA | NA | NA |
| rs1228024 | body lean mass | myopia | -0.011 | 0.002 | 2.66E-12 | -0.002 | 0.001 | 0.021 | NA | NA | NA |
| rs12347137 | body lean mass | myopia | -0.025 | 0.002 | 2.79E-39 | -0.001 | 0.001 | 0.461 | NA | NA | NA |
| rs12452505 | body lean mass | myopia | -0.019 | 0.002 | 1.68E-17 | -0.002 | 0.001 | 0.092 | NA | NA | NA |
| rs12540011 | body lean mass | myopia | 0.014 | 0.002 | 8.87E-16 | 0.000 | 0.001 | 0.757 | NA | NA | NA |
| rs1260326 | body lean mass | myopia | 0.021 | 0.002 | 4.40E-41 | -0.001 | 0.001 | 0.335 | NA | NA | NA |
| rs12619348 | body lean mass | myopia | -0.013 | 0.002 | 5.97E-09 | 0.000 | 0.001 | 0.824 | NA | NA | NA |
| rs12682601 | body lean mass | myopia | -0.011 | 0.002 | 4.68E-11 | 0.000 | 0.001 | 0.682 | NA | NA | NA |
| rs12713004 | body lean mass | myopia | 0.018 | 0.002 | 6.58E-27 | 0.000 | 0.001 | 0.804 | NA | NA | NA |
| rs12813149 | body lean mass | myopia | -0.010 | 0.002 | 4.89E-08 | -0.001 | 0.001 | 0.265 | NA | NA | NA |
| rs1285990 | body lean mass | myopia | 0.011 | 0.002 | 3.56E-11 | 0.000 | 0.001 | 0.543 | NA | NA | NA |
| rs12879423 | body lean mass | myopia | 0.015 | 0.002 | 2.28E-19 | 0.000 | 0.001 | 0.641 | NA | NA | NA |
| rs12889702 | body lean mass | myopia | 0.009 | 0.002 | 1.15E-08 | 0.001 | 0.001 | 0.446 | NA | NA | NA |
| rs12906197 | body lean mass | myopia | -0.013 | 0.002 | 9.54E-16 | 0.000 | 0.001 | 0.820 | NA | NA | NA |
| rs13124829 | body lean mass | myopia | 0.009 | 0.002 | 2.69E-08 | -0.001 | 0.001 | 0.362 | NA | NA | NA |
| rs13148166 | body lean mass | myopia | -0.017 | 0.002 | 7.37E-28 | -0.001 | 0.001 | 0.345 | NA | NA | NA |
| rs13247154 | body lean mass | myopia | 0.012 | 0.002 | 3.87E-14 | 0.000 | 0.001 | 0.870 | NA | NA | NA |
| rs13294021 | body lean mass | myopia | -0.011 | 0.002 | 9.90E-13 | -0.002 | 0.001 | 0.000 | NA | NA | NA |
| rs13430869 | body lean mass | myopia | 0.016 | 0.002 | 6.30E-19 | 0.000 | 0.001 | 0.614 | NA | NA | NA |
| rs1351394 | body lean mass | myopia | -0.027 | 0.002 | 3.38E-70 | 0.001 | 0.001 | 0.129 | NA | NA | NA |
| rs139590892 | body lean mass | myopia | -0.012 | 0.002 | 6.93E-09 | 0.001 | 0.001 | 0.448 | NA | NA | NA |
| rs1407031 | body lean mass | myopia | -0.010 | 0.002 | 1.45E-09 | 0.000 | 0.001 | 0.579 | NA | NA | NA |
| rs141403611 | body lean mass | myopia | -0.015 | 0.003 | 2.38E-08 | -0.001 | 0.001 | 0.269 | NA | NA | NA |
| rs1431663 | body lean mass | myopia | -0.009 | 0.002 | 4.39E-08 | 0.000 | 0.001 | 0.624 | NA | NA | NA |
| rs143384 | body lean mass | myopia | 0.038 | 0.002 | ######## | 0.001 | 0.001 | 0.045 | NA | NA | NA |
| rs143986132 | body lean mass | myopia | -0.035 | 0.006 | 3.14E-09 | 0.006 | 0.003 | 0.022 | NA | NA | NA |
| rs1452822 | body lean mass | myopia | 0.021 | 0.002 | 3.17E-35 | 0.002 | 0.001 | 0.023 | NA | NA | NA |
| rs146851424 | body lean mass | myopia | 0.065 | 0.005 | 3.84E-35 | 0.002 | 0.002 | 0.348 | NA | NA | NA |
| rs147110934 | body lean mass | myopia | -0.033 | 0.005 | 7.52E-11 | 0.002 | 0.002 | 0.282 | NA | NA | NA |
| rs1472852 | body lean mass | myopia | -0.032 | 0.002 | 5.37E-53 | 0.000 | 0.001 | 0.733 | NA | NA | NA |
| rs1478575 | body lean mass | myopia | 0.020 | 0.002 | 2.20E-33 | -0.001 | 0.001 | 0.441 | NA | NA | NA |
| rs149229890 | body lean mass | myopia | 0.045 | 0.008 | 2.99E-09 | 0.000 | 0.003 | 0.883 | NA | NA | NA |
| rs1521624 | body lean mass | myopia | -0.009 | 0.002 | 3.00E-09 | 0.003 | 0.001 | 0.000 | NA | NA | NA |
| rs153560 | body lean mass | myopia | 0.011 | 0.002 | 9.08E-12 | 0.000 | 0.001 | 0.658 | NA | NA | NA |
| rs1542224 | body lean mass | myopia | 0.015 | 0.002 | 2.00E-17 | 0.000 | 0.001 | 0.682 | NA | NA | NA |
| rs1552234 | body lean mass | myopia | 0.009 | 0.002 | 1.42E-08 | -0.001 | 0.001 | 0.157 | NA | NA | NA |
| rs1573891 | body lean mass | myopia | -0.023 | 0.002 | 3.68E-27 | -0.001 | 0.001 | 0.170 | NA | NA | NA |
| rs1582931 | body lean mass | myopia | -0.020 | 0.002 | 1.23E-39 | -0.001 | 0.001 | 0.451 | NA | NA | NA |
| rs1591806 | body lean mass | myopia | 0.017 | 0.002 | 6.80E-27 | 0.000 | 0.001 | 0.955 | NA | NA | NA |
| rs161799 | body lean mass | myopia | -0.009 | 0.002 | 3.78E-08 | 0.002 | 0.001 | 0.001 | NA | NA | NA |
| rs16869017 | body lean mass | myopia | 0.018 | 0.003 | 1.28E-10 | 0.003 | 0.001 | 0.017 | NA | NA | NA |
| rs16892552 | body lean mass | myopia | -0.015 | 0.002 | 2.59E-16 | 0.000 | 0.001 | 0.604 | NA | NA | NA |
| rs16942324 | body lean mass | myopia | -0.040 | 0.005 | 1.09E-16 | -0.001 | 0.002 | 0.800 | NA | NA | NA |
| rs16964211 | body lean mass | myopia | -0.021 | 0.004 | 7.70E-09 | 0.002 | 0.002 | 0.220 | NA | NA | NA |
| rs17056859 | body lean mass | myopia | 0.009 | 0.002 | 9.82E-09 | 0.001 | 0.001 | 0.106 | NA | NA | NA |
| rs17157112 | body lean mass | myopia | -0.009 | 0.002 | 2.47E-09 | 0.000 | 0.001 | 0.680 | NA | NA | NA |
| rs1716162 | body lean mass | myopia | -0.013 | 0.002 | 1.18E-11 | -0.001 | 0.001 | 0.103 | NA | NA | NA |
| rs17197114 | body lean mass | myopia | 0.013 | 0.002 | 1.52E-10 | 0.000 | 0.001 | 0.769 | NA | NA | NA |
| rs17363646 | body lean mass | myopia | 0.015 | 0.002 | 7.56E-11 | 0.001 | 0.001 | 0.185 | NA | NA | NA |
| rs17454369 | body lean mass | myopia | 0.022 | 0.003 | 3.08E-11 | -0.001 | 0.001 | 0.416 | NA | NA | NA |
| rs17741497 | body lean mass | myopia | -0.010 | 0.002 | 1.41E-09 | 0.000 | 0.001 | 0.876 | NA | NA | NA |
| rs17770336 | body lean mass | myopia | 0.011 | 0.002 | 3.71E-11 | -0.001 | 0.001 | 0.420 | NA | NA | NA |
| rs1789164 | body lean mass | myopia | 0.010 | 0.002 | 1.21E-10 | -0.001 | 0.001 | 0.181 | NA | NA | NA |
| rs1805165 | body lean mass | myopia | -0.010 | 0.002 | 1.74E-08 | 0.000 | 0.001 | 0.610 | NA | NA | NA |
| rs1815518 | body lean mass | myopia | 0.016 | 0.002 | 5.09E-15 | 0.001 | 0.001 | 0.279 | NA | NA | NA |
| rs183041 | body lean mass | myopia | 0.014 | 0.002 | 5.12E-16 | -0.002 | 0.001 | 0.008 | NA | NA | NA |
| rs1837367 | body lean mass | myopia | 0.009 | 0.002 | 3.42E-09 | -0.002 | 0.001 | 0.009 | NA | NA | NA |
| rs1878528 | body lean mass | myopia | 0.013 | 0.002 | 1.36E-14 | 0.000 | 0.001 | 0.921 | NA | NA | NA |
| rs1881975 | body lean mass | myopia | -0.014 | 0.002 | 4.31E-15 | 0.000 | 0.001 | 0.727 | NA | NA | NA |
| rs1887855 | body lean mass | myopia | -0.010 | 0.002 | 6.03E-09 | -0.002 | 0.001 | 0.034 | NA | NA | NA |
| rs1910252 | body lean mass | myopia | 0.016 | 0.002 | 6.90E-15 | -0.001 | 0.001 | 0.430 | NA | NA | NA |
| rs1927635 | body lean mass | myopia | 0.010 | 0.002 | 1.59E-09 | 0.000 | 0.001 | 0.564 | NA | NA | NA |
| rs1952527 | body lean mass | myopia | -0.012 | 0.002 | 8.84E-14 | 0.000 | 0.001 | 0.706 | NA | NA | NA |
| rs197372 | body lean mass | myopia | 0.010 | 0.002 | 1.34E-09 | -0.001 | 0.001 | 0.163 | NA | NA | NA |
| rs2005172 | body lean mass | myopia | 0.023 | 0.002 | 1.42E-47 | 0.000 | 0.001 | 0.832 | NA | NA | NA |
| rs2073272 | body lean mass | myopia | -0.010 | 0.002 | 2.81E-09 | 0.001 | 0.001 | 0.388 | NA | NA | NA |
| rs2102278 | body lean mass | myopia | 0.011 | 0.002 | 2.41E-11 | 0.001 | 0.001 | 0.436 | NA | NA | NA |
| rs2118663 | body lean mass | myopia | -0.011 | 0.002 | 1.88E-08 | -0.001 | 0.001 | 0.424 | NA | NA | NA |
| rs2134963 | body lean mass | myopia | -0.011 | 0.002 | 1.48E-10 | 0.001 | 0.001 | 0.219 | NA | NA | NA |
| rs2140046 | body lean mass | myopia | -0.013 | 0.002 | 6.97E-17 | 0.000 | 0.001 | 0.711 | NA | NA | NA |
| rs2142331 | body lean mass | myopia | -0.013 | 0.002 | 1.75E-17 | 0.000 | 0.001 | 0.826 | NA | NA | NA |
| rs216193 | body lean mass | myopia | -0.009 | 0.002 | 3.86E-09 | 0.000 | 0.001 | 0.719 | NA | NA | NA |
| rs2197563 | body lean mass | myopia | 0.011 | 0.002 | 3.72E-12 | 0.000 | 0.001 | 0.502 | NA | NA | NA |
| rs2229840 | body lean mass | myopia | 0.022 | 0.002 | 8.04E-25 | -0.001 | 0.001 | 0.214 | NA | NA | NA |
| rs2230033 | body lean mass | myopia | -0.013 | 0.002 | 2.80E-18 | 0.001 | 0.001 | 0.367 | NA | NA | NA |
| rs2252720 | body lean mass | myopia | -0.014 | 0.002 | 1.31E-17 | -0.001 | 0.001 | 0.071 | NA | NA | NA |
| rs2270894 | body lean mass | myopia | -0.017 | 0.002 | 5.75E-17 | 0.000 | 0.001 | 0.929 | NA | NA | NA |
| rs2274116 | body lean mass | myopia | -0.010 | 0.002 | 3.72E-09 | -0.003 | 0.001 | 0.000 | NA | NA | NA |
| rs2277138 | body lean mass | myopia | -0.018 | 0.002 | 3.66E-31 | 0.000 | 0.001 | 0.490 | NA | NA | NA |
| rs2280940 | body lean mass | myopia | 0.012 | 0.002 | 1.92E-12 | -0.001 | 0.001 | 0.479 | NA | NA | NA |
| rs2281175 | body lean mass | myopia | 0.011 | 0.002 | 8.41E-12 | 0.001 | 0.001 | 0.453 | NA | NA | NA |
| rs2289976 | body lean mass | myopia | 0.012 | 0.002 | 2.88E-12 | 0.000 | 0.001 | 0.963 | NA | NA | NA |
| rs2291256 | body lean mass | myopia | 0.016 | 0.003 | 1.06E-08 | 0.003 | 0.001 | 0.009 | NA | NA | NA |
| rs2296316 | body lean mass | myopia | -0.011 | 0.002 | 1.59E-12 | 0.000 | 0.001 | 0.486 | NA | NA | NA |
| rs2305758 | body lean mass | myopia | 0.010 | 0.002 | 3.70E-09 | 0.001 | 0.001 | 0.140 | NA | NA | NA |
| rs2307111 | body lean mass | myopia | -0.018 | 0.002 | 1.14E-30 | -0.002 | 0.001 | 0.009 | NA | NA | NA |
| rs2395617 | body lean mass | myopia | 0.019 | 0.002 | 4.47E-16 | -0.001 | 0.001 | 0.168 | NA | NA | NA |
| rs2396348 | body lean mass | myopia | 0.012 | 0.002 | 2.00E-12 | 0.000 | 0.001 | 0.518 | NA | NA | NA |
| rs2411453 | body lean mass | myopia | -0.012 | 0.002 | 1.74E-15 | 0.001 | 0.001 | 0.042 | NA | NA | NA |
| rs2455561 | body lean mass | myopia | -0.012 | 0.002 | 1.12E-15 | 0.001 | 0.001 | 0.037 | NA | NA | NA |
| rs2457982 | body lean mass | myopia | 0.011 | 0.002 | 1.35E-09 | 0.001 | 0.001 | 0.125 | NA | NA | NA |
| rs247008 | body lean mass | myopia | 0.015 | 0.002 | 3.16E-20 | 0.001 | 0.001 | 0.208 | NA | NA | NA |
| rs2476998 | body lean mass | myopia | 0.010 | 0.002 | 2.03E-08 | 0.002 | 0.001 | 0.014 | NA | NA | NA |
| rs2482357 | body lean mass | myopia | -0.010 | 0.002 | 8.01E-10 | -0.001 | 0.001 | 0.223 | NA | NA | NA |
| rs2490637 | body lean mass | myopia | -0.019 | 0.003 | 6.46E-09 | 0.000 | 0.001 | 0.778 | NA | NA | NA |
| rs2503756 | body lean mass | myopia | 0.010 | 0.002 | 1.02E-10 | -0.001 | 0.001 | 0.117 | NA | NA | NA |
| rs252758 | body lean mass | myopia | -0.017 | 0.002 | 9.42E-22 | -0.001 | 0.001 | 0.253 | NA | NA | NA |
| rs252937 | body lean mass | myopia | -0.010 | 0.002 | 8.20E-09 | 0.000 | 0.001 | 0.859 | NA | NA | NA |
| rs2533879 | body lean mass | myopia | -0.022 | 0.002 | 6.70E-40 | 0.000 | 0.001 | 0.557 | NA | NA | NA |
| rs2539999 | body lean mass | myopia | 0.017 | 0.002 | 1.91E-23 | 0.000 | 0.001 | 0.763 | NA | NA | NA |
| rs2540034 | body lean mass | myopia | 0.015 | 0.002 | 9.35E-21 | -0.001 | 0.001 | 0.393 | NA | NA | NA |
| rs254963 | body lean mass | myopia | -0.010 | 0.002 | 4.19E-10 | 0.000 | 0.001 | 0.996 | NA | NA | NA |
| rs2592831 | body lean mass | myopia | 0.013 | 0.002 | 7.54E-16 | 0.000 | 0.001 | 0.913 | NA | NA | NA |
| rs2602713 | body lean mass | myopia | 0.010 | 0.002 | 3.58E-11 | 0.000 | 0.001 | 0.491 | NA | NA | NA |
| rs2678204 | body lean mass | myopia | 0.012 | 0.002 | 2.10E-14 | -0.002 | 0.001 | 0.027 | NA | NA | NA |
| rs2733287 | body lean mass | myopia | 0.009 | 0.002 | 1.83E-09 | 0.000 | 0.001 | 0.794 | NA | NA | NA |
| rs2744965 | body lean mass | myopia | 0.039 | 0.002 | 9.87E-70 | -0.004 | 0.001 | 0.000 | NA | NA | NA |
| rs2803888 | body lean mass | myopia | -0.009 | 0.002 | 3.60E-08 | 0.000 | 0.001 | 0.580 | NA | NA | NA |
| rs281385 | body lean mass | myopia | -0.014 | 0.002 | 5.33E-09 | 0.000 | 0.001 | 0.799 | NA | NA | NA |
| rs2815753 | body lean mass | myopia | 0.010 | 0.002 | 7.42E-10 | 0.002 | 0.001 | 0.001 | NA | NA | NA |
| rs28391281 | body lean mass | myopia | -0.010 | 0.002 | 4.28E-10 | 0.000 | 0.001 | 0.908 | NA | NA | NA |
| rs28413009 | body lean mass | myopia | -0.015 | 0.003 | 4.26E-08 | 0.000 | 0.001 | 0.998 | NA | NA | NA |
| rs284315 | body lean mass | myopia | -0.010 | 0.002 | 1.39E-11 | 0.001 | 0.001 | 0.252 | NA | NA | NA |
| rs28457693 | body lean mass | myopia | 0.027 | 0.002 | 1.04E-27 | -0.001 | 0.001 | 0.486 | NA | NA | NA |
| rs2856666 | body lean mass | myopia | -0.021 | 0.002 | 2.33E-41 | 0.000 | 0.001 | 0.873 | NA | NA | NA |
| rs2866719 | body lean mass | myopia | 0.009 | 0.002 | 4.27E-08 | 0.000 | 0.001 | 0.556 | NA | NA | NA |
| rs2885697 | body lean mass | myopia | -0.020 | 0.002 | 9.77E-35 | 0.000 | 0.001 | 0.675 | NA | NA | NA |
| rs28929474 | body lean mass | myopia | 0.034 | 0.005 | 3.00E-10 | 0.001 | 0.002 | 0.756 | NA | NA | NA |
| rs2897968 | body lean mass | myopia | 0.016 | 0.002 | 2.38E-23 | 0.000 | 0.001 | 0.834 | NA | NA | NA |
| rs2900208 | body lean mass | myopia | 0.014 | 0.002 | 1.10E-17 | 0.000 | 0.001 | 0.507 | NA | NA | NA |
| rs292168 | body lean mass | myopia | -0.014 | 0.002 | 8.64E-20 | 0.000 | 0.001 | 0.833 | NA | NA | NA |
| rs2979655 | body lean mass | myopia | 0.012 | 0.002 | 8.58E-09 | -0.001 | 0.001 | 0.197 | NA | NA | NA |
| rs299370 | body lean mass | myopia | -0.010 | 0.002 | 1.00E-09 | 0.002 | 0.001 | 0.017 | NA | NA | NA |
| rs30235 | body lean mass | myopia | 0.009 | 0.002 | 7.28E-09 | 0.000 | 0.001 | 0.757 | NA | NA | NA |
| rs310302 | body lean mass | myopia | -0.009 | 0.002 | 1.81E-08 | -0.001 | 0.001 | 0.396 | NA | NA | NA |
| rs310796 | body lean mass | myopia | 0.011 | 0.002 | 5.26E-12 | 0.001 | 0.001 | 0.415 | NA | NA | NA |
| rs3110496 | body lean mass | myopia | 0.010 | 0.002 | 6.21E-09 | -0.001 | 0.001 | 0.368 | NA | NA | NA |
| rs3116201 | body lean mass | myopia | -0.018 | 0.003 | 2.47E-12 | -0.001 | 0.001 | 0.200 | NA | NA | NA |
| rs3118915 | body lean mass | myopia | -0.032 | 0.002 | 2.32E-64 | -0.001 | 0.001 | 0.286 | NA | NA | NA |
| rs3212260 | body lean mass | myopia | 0.013 | 0.002 | 5.69E-14 | 0.000 | 0.001 | 0.559 | NA | NA | NA |
| rs324010 | body lean mass | myopia | -0.009 | 0.002 | 1.87E-08 | -0.002 | 0.001 | 0.018 | NA | NA | NA |
| rs33967909 | body lean mass | myopia | 0.015 | 0.002 | 3.17E-16 | 0.000 | 0.001 | 0.839 | NA | NA | NA |
| rs34028346 | body lean mass | myopia | 0.012 | 0.002 | 3.00E-08 | -0.001 | 0.001 | 0.552 | NA | NA | NA |
| rs343935 | body lean mass | myopia | 0.013 | 0.002 | 3.27E-10 | 0.002 | 0.001 | 0.096 | NA | NA | NA |
| rs34517439 | body lean mass | myopia | 0.036 | 0.002 | 2.46E-52 | 0.000 | 0.001 | 0.929 | NA | NA | NA |
| rs34693680 | body lean mass | myopia | 0.014 | 0.002 | 9.82E-10 | 0.000 | 0.001 | 0.984 | NA | NA | NA |
| rs34776209 | body lean mass | myopia | -0.018 | 0.002 | 4.01E-25 | 0.000 | 0.001 | 0.889 | NA | NA | NA |
| rs34879158 | body lean mass | myopia | -0.021 | 0.002 | 4.56E-34 | -0.001 | 0.001 | 0.047 | NA | NA | NA |
| rs35233301 | body lean mass | myopia | 0.011 | 0.002 | 5.02E-11 | -0.002 | 0.001 | 0.039 | NA | NA | NA |
| rs35276559 | body lean mass | myopia | -0.009 | 0.002 | 4.82E-08 | 0.000 | 0.001 | 0.887 | NA | NA | NA |
| rs35309034 | body lean mass | myopia | -0.017 | 0.002 | 4.24E-21 | -0.001 | 0.001 | 0.342 | NA | NA | NA |
| rs35436119 | body lean mass | myopia | 0.018 | 0.003 | 1.25E-09 | -0.001 | 0.001 | 0.628 | NA | NA | NA |
| rs35506085 | body lean mass | myopia | -0.020 | 0.002 | 2.29E-22 | 0.001 | 0.001 | 0.390 | NA | NA | NA |
| rs35665085 | body lean mass | myopia | -0.019 | 0.003 | 1.17E-08 | -0.002 | 0.001 | 0.099 | NA | NA | NA |
| rs35710322 | body lean mass | myopia | 0.012 | 0.002 | 7.33E-16 | -0.001 | 0.001 | 0.130 | NA | NA | NA |
| rs357501 | body lean mass | myopia | 0.011 | 0.002 | 1.85E-11 | 0.000 | 0.001 | 0.568 | NA | NA | NA |
| rs35756741 | body lean mass | myopia | -0.019 | 0.003 | 1.69E-12 | 0.000 | 0.001 | 0.684 | NA | NA | NA |
| rs35804313 | body lean mass | myopia | -0.012 | 0.002 | 8.27E-10 | 0.000 | 0.001 | 0.870 | NA | NA | NA |
| rs35874463 | body lean mass | myopia | 0.022 | 0.003 | 1.20E-11 | -0.003 | 0.001 | 0.027 | NA | NA | NA |
| rs36000545 | body lean mass | myopia | -0.015 | 0.002 | 2.01E-21 | 0.000 | 0.001 | 0.752 | NA | NA | NA |
| rs36100359 | body lean mass | myopia | -0.013 | 0.002 | 1.06E-08 | 0.001 | 0.001 | 0.546 | NA | NA | NA |
| rs3738449 | body lean mass | myopia | -0.012 | 0.002 | 1.15E-12 | 0.000 | 0.001 | 0.627 | NA | NA | NA |
| rs3740591 | body lean mass | myopia | 0.013 | 0.002 | 9.64E-17 | 0.000 | 0.001 | 0.623 | NA | NA | NA |
| rs3759094 | body lean mass | myopia | -0.013 | 0.002 | 1.18E-14 | 0.000 | 0.001 | 0.854 | NA | NA | NA |
| rs3765351 | body lean mass | myopia | -0.011 | 0.002 | 1.56E-12 | 0.000 | 0.001 | 0.840 | NA | NA | NA |
| rs3778157 | body lean mass | myopia | 0.012 | 0.002 | 4.28E-09 | 0.001 | 0.001 | 0.371 | NA | NA | NA |
| rs3783256 | body lean mass | myopia | -0.010 | 0.002 | 1.41E-09 | 0.000 | 0.001 | 0.475 | NA | NA | NA |
| rs3798519 | body lean mass | myopia | 0.021 | 0.002 | 2.26E-25 | 0.002 | 0.001 | 0.047 | NA | NA | NA |
| rs3800963 | body lean mass | myopia | -0.009 | 0.002 | 9.65E-09 | 0.000 | 0.001 | 0.560 | NA | NA | NA |
| rs3803286 | body lean mass | myopia | -0.010 | 0.002 | 4.12E-09 | 0.000 | 0.001 | 0.632 | NA | NA | NA |
| rs3809570 | body lean mass | myopia | 0.013 | 0.002 | 8.54E-14 | 0.003 | 0.001 | 0.001 | NA | NA | NA |
| rs3810291 | body lean mass | myopia | 0.017 | 0.002 | 6.10E-25 | 0.000 | 0.001 | 0.886 | NA | NA | NA |
| rs3814877 | body lean mass | myopia | 0.023 | 0.002 | 1.89E-50 | 0.000 | 0.001 | 0.770 | NA | NA | NA |
| rs3818416 | body lean mass | myopia | 0.016 | 0.002 | 8.13E-18 | 0.000 | 0.001 | 0.833 | NA | NA | NA |
| rs3843751 | body lean mass | myopia | -0.014 | 0.002 | 3.01E-17 | 0.000 | 0.001 | 0.883 | NA | NA | NA |
| rs3845344 | body lean mass | myopia | 0.009 | 0.002 | 2.68E-08 | 0.000 | 0.001 | 0.766 | NA | NA | NA |
| rs3853252 | body lean mass | myopia | 0.016 | 0.002 | 9.61E-25 | 0.000 | 0.001 | 0.920 | NA | NA | NA |
| rs390192 | body lean mass | myopia | -0.009 | 0.002 | 1.75E-09 | -0.001 | 0.001 | 0.067 | NA | NA | NA |
| rs4073717 | body lean mass | myopia | -0.018 | 0.002 | 3.15E-21 | 0.000 | 0.001 | 0.717 | NA | NA | NA |
| rs4076108 | body lean mass | myopia | 0.010 | 0.002 | 2.08E-08 | 0.001 | 0.001 | 0.101 | NA | NA | NA |
| rs41271299 | body lean mass | myopia | 0.044 | 0.003 | 1.94E-36 | -0.001 | 0.001 | 0.710 | NA | NA | NA |
| rs41311445 | body lean mass | myopia | -0.024 | 0.003 | 9.26E-21 | 0.001 | 0.001 | 0.226 | NA | NA | NA |
| rs42044 | body lean mass | myopia | 0.029 | 0.002 | 3.21E-61 | 0.001 | 0.001 | 0.150 | NA | NA | NA |
| rs4235012 | body lean mass | myopia | 0.009 | 0.002 | 6.70E-09 | 0.000 | 0.001 | 0.525 | NA | NA | NA |
| rs4240326 | body lean mass | myopia | -0.022 | 0.002 | 4.83E-46 | 0.002 | 0.001 | 0.023 | NA | NA | NA |
| rs4282339 | body lean mass | myopia | -0.017 | 0.002 | 5.97E-20 | 0.001 | 0.001 | 0.324 | NA | NA | NA |
| rs4341996 | body lean mass | myopia | -0.011 | 0.002 | 1.26E-08 | 0.000 | 0.001 | 0.697 | NA | NA | NA |
| rs4472800 | body lean mass | myopia | 0.010 | 0.002 | 2.19E-10 | 0.001 | 0.001 | 0.401 | NA | NA | NA |
| rs4627713 | body lean mass | myopia | 0.032 | 0.005 | 4.69E-12 | 0.005 | 0.002 | 0.009 | NA | NA | NA |
| rs4635681 | body lean mass | myopia | 0.012 | 0.002 | 7.23E-09 | -0.001 | 0.001 | 0.413 | NA | NA | NA |
| rs4680 | body lean mass | myopia | 0.008 | 0.002 | 3.69E-08 | -0.001 | 0.001 | 0.165 | NA | NA | NA |
| rs4752689 | body lean mass | myopia | 0.011 | 0.002 | 3.47E-13 | 0.001 | 0.001 | 0.034 | NA | NA | NA |
| rs475591 | body lean mass | myopia | 0.011 | 0.002 | 2.55E-12 | 0.002 | 0.001 | 0.021 | NA | NA | NA |
| rs4782286 | body lean mass | myopia | -0.012 | 0.002 | 9.64E-11 | 0.001 | 0.001 | 0.127 | NA | NA | NA |
| rs4800670 | body lean mass | myopia | -0.009 | 0.002 | 2.55E-08 | -0.001 | 0.001 | 0.167 | NA | NA | NA |
| rs4819021 | body lean mass | myopia | -0.010 | 0.002 | 9.95E-11 | 0.001 | 0.001 | 0.419 | NA | NA | NA |
| rs4858697 | body lean mass | myopia | 0.012 | 0.002 | 1.21E-15 | -0.001 | 0.001 | 0.395 | NA | NA | NA |
| rs4865956 | body lean mass | myopia | -0.011 | 0.002 | 1.11E-11 | 0.000 | 0.001 | 0.847 | NA | NA | NA |
| rs4980067 | body lean mass | myopia | -0.012 | 0.002 | 3.21E-14 | 0.000 | 0.001 | 0.589 | NA | NA | NA |
| rs4980826 | body lean mass | myopia | 0.009 | 0.002 | 3.75E-08 | 0.000 | 0.001 | 0.889 | NA | NA | NA |
| rs4985148 | body lean mass | myopia | -0.011 | 0.002 | 1.85E-11 | 0.000 | 0.001 | 0.933 | NA | NA | NA |
| rs508347 | body lean mass | myopia | -0.013 | 0.002 | 3.71E-15 | 0.000 | 0.001 | 0.630 | NA | NA | NA |
| rs509035 | body lean mass | myopia | 0.018 | 0.002 | 2.84E-28 | 0.000 | 0.001 | 0.826 | NA | NA | NA |
| rs545608 | body lean mass | myopia | 0.026 | 0.002 | 7.65E-44 | -0.001 | 0.001 | 0.225 | NA | NA | NA |
| rs55681913 | body lean mass | myopia | 0.028 | 0.003 | 1.76E-28 | -0.001 | 0.001 | 0.214 | NA | NA | NA |
| rs55726687 | body lean mass | myopia | 0.015 | 0.002 | 4.96E-15 | -0.002 | 0.001 | 0.047 | NA | NA | NA |
| rs55758152 | body lean mass | myopia | 0.012 | 0.002 | 6.27E-13 | -0.001 | 0.001 | 0.451 | NA | NA | NA |
| rs55831773 | body lean mass | myopia | -0.016 | 0.002 | 6.89E-16 | -0.001 | 0.001 | 0.356 | NA | NA | NA |
| rs55872725 | body lean mass | myopia | 0.037 | 0.002 | ######## | 0.001 | 0.001 | 0.456 | NA | NA | NA |
| rs57126421 | body lean mass | myopia | -0.010 | 0.002 | 8.23E-09 | -0.001 | 0.001 | 0.466 | NA | NA | NA |
| rs57153895 | body lean mass | myopia | -0.012 | 0.002 | 3.41E-14 | 0.000 | 0.001 | 0.594 | NA | NA | NA |
| rs5742915 | body lean mass | myopia | 0.010 | 0.002 | 5.54E-10 | 0.000 | 0.001 | 0.892 | NA | NA | NA |
| rs5752989 | body lean mass | myopia | -0.010 | 0.002 | 4.14E-11 | -0.001 | 0.001 | 0.064 | NA | NA | NA |
| rs57635800 | body lean mass | myopia | 0.013 | 0.002 | 1.35E-13 | 0.000 | 0.001 | 0.627 | NA | NA | NA |
| rs57636386 | body lean mass | myopia | -0.025 | 0.003 | 9.95E-19 | 0.000 | 0.001 | 0.733 | NA | NA | NA |
| rs58857770 | body lean mass | myopia | -0.012 | 0.002 | 7.85E-14 | 0.000 | 0.001 | 0.899 | NA | NA | NA |
| rs599004 | body lean mass | myopia | -0.015 | 0.002 | 1.77E-17 | 0.001 | 0.001 | 0.359 | NA | NA | NA |
| rs59985551 | body lean mass | myopia | -0.018 | 0.002 | 1.50E-22 | -0.004 | 0.001 | 0.000 | NA | NA | NA |
| rs60077625 | body lean mass | myopia | 0.014 | 0.002 | 4.09E-18 | 0.002 | 0.001 | 0.010 | NA | NA | NA |
| rs6032233 | body lean mass | myopia | 0.012 | 0.002 | 2.58E-10 | 0.000 | 0.001 | 0.718 | NA | NA | NA |
| rs607472 | body lean mass | myopia | 0.012 | 0.002 | 1.44E-13 | 0.001 | 0.001 | 0.424 | NA | NA | NA |
| rs60804050 | body lean mass | myopia | -0.011 | 0.002 | 1.16E-09 | -0.001 | 0.001 | 0.294 | NA | NA | NA |
| rs6081869 | body lean mass | myopia | -0.010 | 0.002 | 1.82E-10 | 0.000 | 0.001 | 0.780 | NA | NA | NA |
| rs6085659 | body lean mass | myopia | -0.010 | 0.002 | 8.95E-10 | 0.001 | 0.001 | 0.274 | NA | NA | NA |
| rs6142059 | body lean mass | myopia | 0.011 | 0.002 | 7.89E-13 | 0.000 | 0.001 | 0.547 | NA | NA | NA |
| rs61729527 | body lean mass | myopia | -0.025 | 0.003 | 5.45E-13 | 0.000 | 0.002 | 0.796 | NA | NA | NA |
| rs61862463 | body lean mass | myopia | 0.030 | 0.005 | 8.09E-09 | 0.001 | 0.002 | 0.585 | NA | NA | NA |
| rs61941043 | body lean mass | myopia | 0.049 | 0.008 | 8.01E-10 | 0.001 | 0.003 | 0.777 | NA | NA | NA |
| rs61954257 | body lean mass | myopia | 0.012 | 0.002 | 2.36E-14 | -0.002 | 0.001 | 0.012 | NA | NA | NA |
| rs62070645 | body lean mass | myopia | -0.024 | 0.002 | 3.28E-44 | -0.001 | 0.001 | 0.350 | NA | NA | NA |
| rs62107261 | body lean mass | myopia | -0.054 | 0.004 | 7.00E-51 | -0.001 | 0.002 | 0.411 | NA | NA | NA |
| rs62246314 | body lean mass | myopia | 0.014 | 0.003 | 4.72E-08 | 0.001 | 0.001 | 0.565 | NA | NA | NA |
| rs62275882 | body lean mass | myopia | -0.014 | 0.002 | 1.63E-10 | 0.000 | 0.001 | 0.747 | NA | NA | NA |
| rs6235 | body lean mass | myopia | 0.016 | 0.002 | 4.14E-20 | 0.001 | 0.001 | 0.453 | NA | NA | NA |
| rs62515437 | body lean mass | myopia | 0.020 | 0.002 | 1.10E-28 | 0.000 | 0.001 | 0.654 | NA | NA | NA |
| rs62621197 | body lean mass | myopia | -0.036 | 0.004 | 9.31E-18 | -0.002 | 0.002 | 0.173 | NA | NA | NA |
| rs62621812 | body lean mass | myopia | 0.049 | 0.006 | 1.20E-18 | 0.000 | 0.002 | 0.886 | NA | NA | NA |
| rs6445198 | body lean mass | myopia | -0.009 | 0.002 | 3.08E-09 | 0.001 | 0.001 | 0.138 | NA | NA | NA |
| rs6505781 | body lean mass | myopia | -0.010 | 0.002 | 2.65E-08 | -0.001 | 0.001 | 0.390 | NA | NA | NA |
| rs6570509 | body lean mass | myopia | -0.018 | 0.002 | 1.55E-25 | -0.001 | 0.001 | 0.243 | NA | NA | NA |
| rs658957 | body lean mass | myopia | -0.012 | 0.002 | 1.80E-08 | 0.000 | 0.001 | 0.585 | NA | NA | NA |
| rs662115 | body lean mass | myopia | 0.010 | 0.002 | 4.22E-10 | 0.000 | 0.001 | 0.768 | NA | NA | NA |
| rs66922415 | body lean mass | myopia | 0.044 | 0.002 | ######## | -0.001 | 0.001 | 0.257 | NA | NA | NA |
| rs67551338 | body lean mass | myopia | 0.025 | 0.003 | 5.58E-15 | -0.001 | 0.001 | 0.407 | NA | NA | NA |
| rs6762578 | body lean mass | myopia | 0.016 | 0.002 | 6.68E-19 | 0.001 | 0.001 | 0.438 | NA | NA | NA |
| rs6800021 | body lean mass | myopia | 0.014 | 0.002 | 1.13E-19 | 0.000 | 0.001 | 0.614 | NA | NA | NA |
| rs6821305 | body lean mass | myopia | 0.012 | 0.002 | 8.05E-15 | 0.000 | 0.001 | 0.662 | NA | NA | NA |
| rs6873192 | body lean mass | myopia | -0.012 | 0.002 | 3.90E-15 | -0.001 | 0.001 | 0.381 | NA | NA | NA |
| rs6874142 | body lean mass | myopia | 0.020 | 0.003 | 2.13E-15 | -0.004 | 0.001 | 0.000 | NA | NA | NA |
| rs6902789 | body lean mass | myopia | 0.010 | 0.002 | 1.10E-10 | 0.000 | 0.001 | 0.588 | NA | NA | NA |
| rs6975015 | body lean mass | myopia | 0.018 | 0.002 | 1.68E-13 | 0.000 | 0.001 | 0.819 | NA | NA | NA |
| rs7047694 | body lean mass | myopia | 0.011 | 0.002 | 1.30E-11 | 0.000 | 0.001 | 0.769 | NA | NA | NA |
| rs7077783 | body lean mass | myopia | -0.015 | 0.002 | 1.36E-11 | 0.000 | 0.001 | 0.651 | NA | NA | NA |
| rs7080472 | body lean mass | myopia | 0.015 | 0.002 | 2.28E-22 | 0.000 | 0.001 | 0.873 | NA | NA | NA |
| rs7097872 | body lean mass | myopia | 0.012 | 0.002 | 2.19E-14 | 0.000 | 0.001 | 0.918 | NA | NA | NA |
| rs7129320 | body lean mass | myopia | -0.023 | 0.002 | 7.00E-30 | -0.002 | 0.001 | 0.015 | NA | NA | NA |
| rs7132908 | body lean mass | myopia | 0.017 | 0.002 | 7.52E-26 | 0.000 | 0.001 | 0.731 | NA | NA | NA |
| rs7134283 | body lean mass | myopia | -0.013 | 0.002 | 1.98E-13 | -0.001 | 0.001 | 0.161 | NA | NA | NA |
| rs71385734 | body lean mass | myopia | -0.024 | 0.002 | 3.58E-31 | -0.001 | 0.001 | 0.359 | NA | NA | NA |
| rs7141420 | body lean mass | myopia | 0.012 | 0.002 | 1.65E-14 | 0.001 | 0.001 | 0.285 | NA | NA | NA |
| rs71414738 | body lean mass | myopia | 0.011 | 0.002 | 1.55E-08 | 0.001 | 0.001 | 0.142 | NA | NA | NA |
| rs71484923 | body lean mass | myopia | 0.011 | 0.002 | 1.99E-08 | 0.000 | 0.001 | 0.826 | NA | NA | NA |
| rs7148516 | body lean mass | myopia | -0.009 | 0.002 | 1.53E-08 | -0.001 | 0.001 | 0.396 | NA | NA | NA |
| rs7190477 | body lean mass | myopia | 0.009 | 0.002 | 1.93E-09 | 0.000 | 0.001 | 0.888 | NA | NA | NA |
| rs7205337 | body lean mass | myopia | 0.016 | 0.002 | 3.95E-17 | 0.000 | 0.001 | 0.745 | NA | NA | NA |
| rs7214743 | body lean mass | myopia | -0.016 | 0.002 | 2.11E-22 | 0.000 | 0.001 | 0.694 | NA | NA | NA |
| rs7229351 | body lean mass | myopia | -0.010 | 0.002 | 5.53E-11 | 0.000 | 0.001 | 0.496 | NA | NA | NA |
| rs723149 | body lean mass | myopia | -0.013 | 0.002 | 9.40E-17 | 0.000 | 0.001 | 0.487 | NA | NA | NA |
| rs7235010 | body lean mass | myopia | 0.027 | 0.002 | 2.75E-47 | 0.000 | 0.001 | 0.711 | NA | NA | NA |
| rs724016 | body lean mass | myopia | 0.030 | 0.002 | 9.22E-84 | -0.002 | 0.001 | 0.007 | NA | NA | NA |
| rs7250927 | body lean mass | myopia | -0.011 | 0.002 | 3.22E-11 | -0.001 | 0.001 | 0.356 | NA | NA | NA |
| rs72656010 | body lean mass | myopia | -0.036 | 0.002 | 5.13E-55 | 0.000 | 0.001 | 0.616 | NA | NA | NA |
| rs72660086 | body lean mass | myopia | 0.015 | 0.002 | 3.61E-15 | -0.001 | 0.001 | 0.376 | NA | NA | NA |
| rs72699866 | body lean mass | myopia | 0.013 | 0.002 | 1.09E-11 | 0.002 | 0.001 | 0.023 | NA | NA | NA |
| rs72703414 | body lean mass | myopia | -0.018 | 0.003 | 1.20E-08 | 0.002 | 0.001 | 0.091 | NA | NA | NA |
| rs72755233 | body lean mass | myopia | -0.014 | 0.002 | 1.17E-08 | -0.002 | 0.001 | 0.024 | NA | NA | NA |
| rs72801843 | body lean mass | myopia | 0.016 | 0.002 | 1.99E-21 | 0.002 | 0.001 | 0.023 | NA | NA | NA |
| rs72828807 | body lean mass | myopia | 0.010 | 0.002 | 6.29E-09 | 0.000 | 0.001 | 0.670 | NA | NA | NA |
| rs72885917 | body lean mass | myopia | -0.022 | 0.002 | 2.70E-36 | 0.000 | 0.001 | 0.972 | NA | NA | NA |
| rs73052033 | body lean mass | myopia | -0.016 | 0.002 | 3.10E-16 | -0.001 | 0.001 | 0.210 | NA | NA | NA |
| rs730536 | body lean mass | myopia | -0.009 | 0.002 | 2.50E-08 | 0.000 | 0.001 | 0.711 | NA | NA | NA |
| rs7305516 | body lean mass | myopia | -0.011 | 0.002 | 2.19E-12 | 0.000 | 0.001 | 0.937 | NA | NA | NA |
| rs73175572 | body lean mass | myopia | 0.028 | 0.002 | 1.56E-29 | 0.000 | 0.001 | 0.745 | NA | NA | NA |
| rs73619441 | body lean mass | myopia | -0.016 | 0.002 | 1.49E-13 | -0.002 | 0.001 | 0.027 | NA | NA | NA |
| rs73966422 | body lean mass | myopia | 0.013 | 0.002 | 4.67E-08 | 0.000 | 0.001 | 0.981 | NA | NA | NA |
| rs74048171 | body lean mass | myopia | -0.010 | 0.002 | 1.05E-08 | -0.001 | 0.001 | 0.412 | NA | NA | NA |
| rs74494415 | body lean mass | myopia | -0.030 | 0.004 | 2.53E-14 | 0.002 | 0.002 | 0.183 | NA | NA | NA |
| rs74565893 | body lean mass | myopia | -0.040 | 0.007 | 3.41E-08 | -0.002 | 0.003 | 0.551 | NA | NA | NA |
| rs7460093 | body lean mass | myopia | 0.009 | 0.002 | 1.93E-09 | 0.000 | 0.001 | 0.959 | NA | NA | NA |
| rs748457 | body lean mass | myopia | 0.009 | 0.002 | 1.58E-09 | 0.000 | 0.001 | 0.499 | NA | NA | NA |
| rs7559547 | body lean mass | myopia | 0.032 | 0.002 | 6.60E-57 | -0.002 | 0.001 | 0.062 | NA | NA | NA |
| rs7584391 | body lean mass | myopia | -0.012 | 0.002 | 4.90E-09 | 0.000 | 0.001 | 0.644 | NA | NA | NA |
| rs7624428 | body lean mass | myopia | 0.011 | 0.002 | 8.34E-10 | 0.001 | 0.001 | 0.429 | NA | NA | NA |
| rs7628343 | body lean mass | myopia | 0.015 | 0.003 | 5.27E-09 | -0.001 | 0.001 | 0.446 | NA | NA | NA |
| rs76798800 | body lean mass | myopia | 0.023 | 0.002 | 1.27E-38 | -0.001 | 0.001 | 0.493 | NA | NA | NA |
| rs768023 | body lean mass | myopia | 0.019 | 0.002 | 2.93E-33 | 0.000 | 0.001 | 0.485 | NA | NA | NA |
| rs76895963 | body lean mass | myopia | 0.103 | 0.006 | 7.25E-67 | 0.002 | 0.003 | 0.548 | NA | NA | NA |
| rs7728690 | body lean mass | myopia | -0.011 | 0.002 | 3.26E-12 | 0.001 | 0.001 | 0.108 | NA | NA | NA |
| rs7730885 | body lean mass | myopia | 0.015 | 0.002 | 3.16E-20 | 0.002 | 0.001 | 0.026 | NA | NA | NA |
| rs7731023 | body lean mass | myopia | 0.010 | 0.002 | 7.59E-10 | -0.002 | 0.001 | 0.005 | NA | NA | NA |
| rs77392989 | body lean mass | myopia | -0.014 | 0.002 | 5.47E-12 | -0.001 | 0.001 | 0.120 | NA | NA | NA |
| rs7740107 | body lean mass | myopia | -0.029 | 0.002 | 4.97E-61 | 0.003 | 0.001 | 0.000 | NA | NA | NA |
| rs7781964 | body lean mass | myopia | 0.013 | 0.002 | 1.35E-11 | 0.002 | 0.001 | 0.009 | NA | NA | NA |
| rs7815955 | body lean mass | myopia | -0.017 | 0.002 | 1.09E-19 | -0.002 | 0.001 | 0.030 | NA | NA | NA |
| rs78378222 | body lean mass | myopia | 0.089 | 0.007 | 9.38E-37 | -0.003 | 0.003 | 0.394 | NA | NA | NA |
| rs78812993 | body lean mass | myopia | -0.023 | 0.003 | 4.36E-11 | 0.003 | 0.002 | 0.088 | NA | NA | NA |
| rs78818722 | body lean mass | myopia | 0.023 | 0.003 | 3.03E-14 | 0.001 | 0.001 | 0.307 | NA | NA | NA |
| rs78964719 | body lean mass | myopia | 0.017 | 0.003 | 1.92E-08 | -0.001 | 0.001 | 0.286 | NA | NA | NA |
| rs7910087 | body lean mass | myopia | -0.012 | 0.002 | 2.80E-15 | 0.000 | 0.001 | 0.911 | NA | NA | NA |
| rs79266482 | body lean mass | myopia | 0.010 | 0.002 | 4.05E-08 | -0.001 | 0.001 | 0.413 | NA | NA | NA |
| rs7952436 | body lean mass | myopia | -0.033 | 0.003 | 3.09E-32 | 0.002 | 0.001 | 0.097 | NA | NA | NA |
| rs7977788 | body lean mass | myopia | 0.026 | 0.002 | 5.52E-46 | 0.001 | 0.001 | 0.322 | NA | NA | NA |
| rs79883557 | body lean mass | myopia | -0.018 | 0.003 | 1.97E-10 | 0.000 | 0.001 | 0.755 | NA | NA | NA |
| rs7994814 | body lean mass | myopia | 0.011 | 0.002 | 2.36E-12 | 0.001 | 0.001 | 0.398 | NA | NA | NA |
| rs8007058 | body lean mass | myopia | 0.010 | 0.002 | 4.13E-08 | -0.001 | 0.001 | 0.063 | NA | NA | NA |
| rs8007644 | body lean mass | myopia | 0.010 | 0.002 | 2.14E-09 | -0.001 | 0.001 | 0.249 | NA | NA | NA |
| rs8059189 | body lean mass | myopia | -0.011 | 0.002 | 2.73E-12 | 0.000 | 0.001 | 0.610 | NA | NA | NA |
| rs8123912 | body lean mass | myopia | 0.011 | 0.002 | 5.26E-10 | -0.001 | 0.001 | 0.072 | NA | NA | NA |
| rs815341 | body lean mass | myopia | 0.010 | 0.002 | 2.24E-09 | -0.001 | 0.001 | 0.174 | NA | NA | NA |
| rs836519 | body lean mass | myopia | 0.012 | 0.002 | 2.74E-10 | 0.002 | 0.001 | 0.045 | NA | NA | NA |
| rs9257319 | body lean mass | myopia | -0.019 | 0.002 | 1.65E-19 | 0.000 | 0.001 | 0.783 | NA | NA | NA |
| rs9314420 | body lean mass | myopia | -0.009 | 0.002 | 4.36E-09 | -0.001 | 0.001 | 0.208 | NA | NA | NA |
| rs9317002 | body lean mass | myopia | 0.009 | 0.002 | 2.39E-09 | 0.000 | 0.001 | 0.847 | NA | NA | NA |
| rs931874 | body lean mass | myopia | 0.009 | 0.002 | 5.23E-09 | 0.000 | 0.001 | 0.882 | NA | NA | NA |
| rs9327336 | body lean mass | myopia | 0.010 | 0.002 | 7.42E-10 | 0.000 | 0.001 | 0.901 | NA | NA | NA |
| rs9343977 | body lean mass | myopia | -0.013 | 0.002 | 7.02E-18 | 0.000 | 0.001 | 0.787 | NA | NA | NA |
| rs9379084 | body lean mass | myopia | -0.015 | 0.002 | 1.25E-09 | 0.000 | 0.001 | 0.646 | NA | NA | NA |
| rs9381350 | body lean mass | myopia | 0.010 | 0.002 | 3.95E-09 | 0.001 | 0.001 | 0.370 | NA | NA | NA |
| rs946197 | body lean mass | myopia | 0.016 | 0.002 | 3.92E-18 | 0.001 | 0.001 | 0.522 | NA | NA | NA |
| rs9480947 | body lean mass | myopia | -0.014 | 0.002 | 2.55E-20 | -0.001 | 0.001 | 0.313 | NA | NA | NA |
| rs9512661 | body lean mass | myopia | -0.009 | 0.002 | 1.61E-08 | 0.000 | 0.001 | 0.748 | NA | NA | NA |
| rs9540493 | body lean mass | myopia | -0.008 | 0.002 | 4.66E-08 | 0.001 | 0.001 | 0.370 | NA | NA | NA |
| rs9788443 | body lean mass | myopia | 0.020 | 0.004 | 3.73E-08 | 0.002 | 0.002 | 0.184 | NA | NA | NA |
| rs981002 | body lean mass | myopia | -0.011 | 0.002 | 9.01E-11 | 0.001 | 0.001 | 0.196 | NA | NA | NA |
| rs9826759 | body lean mass | myopia | 0.015 | 0.002 | 1.92E-19 | -0.002 | 0.001 | 0.017 | NA | NA | NA |
| rs9861443 | body lean mass | myopia | 0.012 | 0.002 | 3.26E-12 | 0.000 | 0.001 | 0.721 | NA | NA | NA |
| rs9915532 | body lean mass | myopia | -0.016 | 0.002 | 2.57E-15 | 0.002 | 0.001 | 0.020 | NA | NA | NA |
| rs9925273 | body lean mass | myopia | -0.013 | 0.002 | 1.10E-10 | 0.002 | 0.001 | 0.072 | NA | NA | NA |
| rs9951619 | body lean mass | myopia | 0.013 | 0.002 | 2.38E-12 | -0.002 | 0.001 | 0.026 | NA | NA | NA |
| rs9960619 | body lean mass | myopia | 0.009 | 0.002 | 1.36E-08 | 0.000 | 0.001 | 0.493 | NA | NA | NA |
| rs9985795 | body lean mass | myopia | -0.009 | 0.002 | 3.95E-09 | 0.001 | 0.001 | 0.027 | NA | NA | NA |
| rs10139160 | Hand grip strength (left) | myopia | 0.010 | 0.002 | 5.63E-09 | -0.001 | 0.001 | 0.332 | NA | NA | NA |
| rs10205394 | Hand grip strength (left) | myopia | -0.012 | 0.002 | 2.38E-08 | 0.000 | 0.001 | 0.777 | NA | NA | NA |
| rs10210654 | Hand grip strength (left) | myopia | 0.014 | 0.002 | 3.19E-10 | 0.001 | 0.001 | 0.485 | NA | NA | NA |
| rs10403906 | Hand grip strength (left) | myopia | -0.010 | 0.002 | 2.87E-09 | 0.000 | 0.001 | 0.520 | NA | NA | NA |
| rs10753139 | Hand grip strength (left) | myopia | 0.015 | 0.002 | 1.06E-16 | 0.000 | 0.001 | 0.819 | NA | NA | NA |
| rs10753823 | Hand grip strength (left) | myopia | -0.011 | 0.002 | 1.51E-08 | -0.002 | 0.001 | 0.024 | NA | NA | NA |
| rs10788958 | Hand grip strength (left) | myopia | 0.015 | 0.002 | 9.23E-16 | -0.001 | 0.001 | 0.267 | NA | NA | NA |
| rs10805877 | Hand grip strength (left) | myopia | 0.012 | 0.002 | 5.08E-09 | 0.001 | 0.001 | 0.354 | NA | NA | NA |
| rs10846071 | Hand grip strength (left) | myopia | -0.018 | 0.002 | 2.49E-24 | -0.001 | 0.001 | 0.436 | NA | NA | NA |
| rs11002322 | Hand grip strength (left) | myopia | -0.010 | 0.002 | 1.68E-08 | 0.001 | 0.001 | 0.093 | NA | NA | NA |
| rs11124957 | Hand grip strength (left) | myopia | -0.011 | 0.002 | 7.23E-11 | 0.001 | 0.001 | 0.114 | NA | NA | NA |
| rs11130333 | Hand grip strength (left) | myopia | 0.011 | 0.002 | 1.36E-09 | 0.001 | 0.001 | 0.268 | NA | NA | NA |
| rs11236203 | Hand grip strength (left) | myopia | -0.015 | 0.002 | 1.33E-17 | 0.000 | 0.001 | 0.750 | NA | NA | NA |
| rs11631697 | Hand grip strength (left) | myopia | 0.011 | 0.002 | 2.39E-08 | 0.002 | 0.001 | 0.033 | NA | NA | NA |
| rs11642430 | Hand grip strength (left) | myopia | 0.011 | 0.002 | 2.64E-09 | 0.000 | 0.001 | 0.782 | NA | NA | NA |
| rs11906450 | Hand grip strength (left) | myopia | 0.011 | 0.002 | 1.50E-08 | -0.001 | 0.001 | 0.076 | NA | NA | NA |
| rs12055234 | Hand grip strength (left) | myopia | -0.010 | 0.002 | 4.39E-08 | -0.001 | 0.001 | 0.236 | NA | NA | NA |
| rs12119893 | Hand grip strength (left) | myopia | -0.015 | 0.003 | 1.52E-08 | -0.001 | 0.001 | 0.369 | NA | NA | NA |
| rs12129704 | Hand grip strength (left) | myopia | -0.016 | 0.002 | 1.83E-13 | 0.000 | 0.001 | 0.592 | NA | NA | NA |
| rs12316046 | Hand grip strength (left) | myopia | -0.019 | 0.002 | 2.06E-25 | 0.000 | 0.001 | 0.470 | NA | NA | NA |
| rs12361415 | Hand grip strength (left) | myopia | 0.014 | 0.002 | 1.29E-12 | 0.000 | 0.001 | 0.775 | NA | NA | NA |
| rs12414407 | Hand grip strength (left) | myopia | 0.012 | 0.002 | 1.40E-10 | 0.001 | 0.001 | 0.450 | NA | NA | NA |
| rs12673062 | Hand grip strength (left) | myopia | -0.013 | 0.002 | 3.29E-09 | -0.001 | 0.001 | 0.451 | NA | NA | NA |
| rs12792358 | Hand grip strength (left) | myopia | -0.025 | 0.004 | 1.02E-12 | 0.004 | 0.001 | 0.002 | NA | NA | NA |
| rs12914702 | Hand grip strength (left) | myopia | 0.012 | 0.002 | 6.19E-09 | 0.000 | 0.001 | 0.532 | NA | NA | NA |
| rs12917449 | Hand grip strength (left) | myopia | -0.013 | 0.002 | 3.19E-09 | -0.001 | 0.001 | 0.414 | NA | NA | NA |
| rs12926737 | Hand grip strength (left) | myopia | -0.017 | 0.002 | 2.86E-14 | 0.000 | 0.001 | 0.752 | NA | NA | NA |
| rs13107325 | Hand grip strength (left) | myopia | -0.028 | 0.003 | 6.10E-17 | -0.004 | 0.001 | 0.001 | NA | NA | NA |
| rs138019 | Hand grip strength (left) | myopia | -0.011 | 0.002 | 2.53E-08 | -0.002 | 0.001 | 0.021 | NA | NA | NA |
| rs143384 | Hand grip strength (left) | myopia | 0.020 | 0.002 | 1.89E-28 | 0.001 | 0.001 | 0.045 | NA | NA | NA |
| rs1442883 | Hand grip strength (left) | myopia | -0.012 | 0.002 | 1.34E-09 | 0.000 | 0.001 | 0.873 | NA | NA | NA |
| rs150330307 | Hand grip strength (left) | myopia | -0.031 | 0.005 | 3.35E-10 | 0.001 | 0.002 | 0.616 | NA | NA | NA |
| rs1550115 | Hand grip strength (left) | myopia | 0.015 | 0.002 | 2.30E-14 | 0.001 | 0.001 | 0.188 | NA | NA | NA |
| rs1551042 | Hand grip strength (left) | myopia | -0.011 | 0.002 | 5.59E-10 | 0.002 | 0.001 | 0.010 | NA | NA | NA |
| rs1556659 | Hand grip strength (left) | myopia | 0.016 | 0.002 | 2.92E-19 | 0.000 | 0.001 | 0.501 | NA | NA | NA |
| rs16896068 | Hand grip strength (left) | myopia | -0.021 | 0.002 | 4.93E-19 | 0.000 | 0.001 | 0.796 | NA | NA | NA |
| rs217181 | Hand grip strength (left) | myopia | 0.013 | 0.002 | 1.03E-08 | 0.000 | 0.001 | 0.690 | NA | NA | NA |
| rs2273555 | Hand grip strength (left) | myopia | 0.011 | 0.002 | 2.09E-09 | -0.002 | 0.001 | 0.010 | NA | NA | NA |
| rs2532111 | Hand grip strength (left) | myopia | 0.011 | 0.002 | 5.04E-10 | 0.000 | 0.001 | 0.724 | NA | NA | NA |
| rs2807504 | Hand grip strength (left) | myopia | 0.012 | 0.002 | 2.92E-09 | 0.000 | 0.001 | 0.717 | NA | NA | NA |
| rs2871865 | Hand grip strength (left) | myopia | -0.024 | 0.003 | 2.96E-18 | 0.000 | 0.001 | 0.768 | NA | NA | NA |
| rs2871960 | Hand grip strength (left) | myopia | 0.012 | 0.002 | 1.70E-11 | -0.002 | 0.001 | 0.006 | NA | NA | NA |
| rs3116600 | Hand grip strength (left) | myopia | -0.018 | 0.002 | 1.11E-16 | -0.001 | 0.001 | 0.252 | NA | NA | NA |
| rs3169733 | Hand grip strength (left) | myopia | 0.011 | 0.002 | 5.47E-09 | -0.001 | 0.001 | 0.105 | NA | NA | NA |
| rs34159998 | Hand grip strength (left) | myopia | -0.024 | 0.004 | 1.60E-08 | 0.000 | 0.002 | 0.807 | NA | NA | NA |
| rs34845616 | Hand grip strength (left) | myopia | 0.012 | 0.002 | 6.66E-09 | 0.000 | 0.001 | 0.799 | NA | NA | NA |
| rs35779564 | Hand grip strength (left) | myopia | -0.010 | 0.002 | 3.78E-08 | 0.000 | 0.001 | 0.601 | NA | NA | NA |
| rs35810656 | Hand grip strength (left) | myopia | 0.010 | 0.002 | 2.30E-08 | 0.001 | 0.001 | 0.328 | NA | NA | NA |
| rs3785456 | Hand grip strength (left) | myopia | 0.015 | 0.002 | 1.92E-15 | 0.000 | 0.001 | 0.952 | NA | NA | NA |
| rs41271299 | Hand grip strength (left) | myopia | 0.024 | 0.004 | 1.54E-09 | -0.001 | 0.001 | 0.710 | NA | NA | NA |
| rs41705 | Hand grip strength (left) | myopia | -0.012 | 0.002 | 7.29E-09 | -0.001 | 0.001 | 0.466 | NA | NA | NA |
| rs417591 | Hand grip strength (left) | myopia | 0.012 | 0.002 | 7.30E-09 | 0.000 | 0.001 | 0.721 | NA | NA | NA |
| rs4234519 | Hand grip strength (left) | myopia | -0.011 | 0.002 | 1.16E-08 | -0.001 | 0.001 | 0.363 | NA | NA | NA |
| rs4308051 | Hand grip strength (left) | myopia | 0.017 | 0.002 | 6.67E-15 | 0.000 | 0.001 | 0.660 | NA | NA | NA |
| rs4363950 | Hand grip strength (left) | myopia | 0.013 | 0.002 | 8.64E-11 | 0.001 | 0.001 | 0.048 | NA | NA | NA |
| rs4380799 | Hand grip strength (left) | myopia | -0.012 | 0.002 | 4.93E-10 | 0.000 | 0.001 | 0.831 | NA | NA | NA |
| rs4398863 | Hand grip strength (left) | myopia | -0.011 | 0.002 | 3.51E-08 | -0.001 | 0.001 | 0.272 | NA | NA | NA |
| rs4553566 | Hand grip strength (left) | myopia | -0.011 | 0.002 | 1.93E-09 | 0.001 | 0.001 | 0.294 | NA | NA | NA |
| rs4575361 | Hand grip strength (left) | myopia | -0.010 | 0.002 | 3.83E-08 | 0.002 | 0.001 | 0.024 | NA | NA | NA |
| rs4621706 | Hand grip strength (left) | myopia | -0.013 | 0.002 | 8.03E-13 | 0.000 | 0.001 | 0.471 | NA | NA | NA |
| rs4713506 | Hand grip strength (left) | myopia | -0.018 | 0.002 | 1.52E-19 | 0.000 | 0.001 | 0.896 | NA | NA | NA |
| rs4784329 | Hand grip strength (left) | myopia | -0.011 | 0.002 | 6.28E-10 | 0.000 | 0.001 | 0.575 | NA | NA | NA |
| rs4886778 | Hand grip strength (left) | myopia | 0.013 | 0.002 | 9.52E-14 | 0.002 | 0.001 | 0.007 | NA | NA | NA |
| rs56187488 | Hand grip strength (left) | myopia | -0.011 | 0.002 | 3.59E-08 | 0.000 | 0.001 | 0.825 | NA | NA | NA |
| rs56338231 | Hand grip strength (left) | myopia | -0.012 | 0.002 | 4.35E-09 | 0.000 | 0.001 | 0.611 | NA | NA | NA |
| rs635538 | Hand grip strength (left) | myopia | -0.024 | 0.003 | 1.00E-14 | 0.000 | 0.001 | 0.687 | NA | NA | NA |
| rs66517261 | Hand grip strength (left) | myopia | -0.012 | 0.002 | 4.30E-09 | 0.000 | 0.001 | 0.707 | NA | NA | NA |
| rs6754903 | Hand grip strength (left) | myopia | 0.016 | 0.002 | 2.15E-12 | 0.001 | 0.001 | 0.325 | NA | NA | NA |
| rs694893 | Hand grip strength (left) | myopia | -0.011 | 0.002 | 2.17E-09 | -0.001 | 0.001 | 0.461 | NA | NA | NA |
| rs6977081 | Hand grip strength (left) | myopia | 0.014 | 0.002 | 3.97E-14 | 0.001 | 0.001 | 0.078 | NA | NA | NA |
| rs700518 | Hand grip strength (left) | myopia | 0.011 | 0.002 | 1.88E-09 | -0.001 | 0.001 | 0.094 | NA | NA | NA |
| rs7196917 | Hand grip strength (left) | myopia | -0.013 | 0.002 | 1.38E-12 | -0.001 | 0.001 | 0.223 | NA | NA | NA |
| rs7222242 | Hand grip strength (left) | myopia | -0.013 | 0.002 | 1.77E-09 | 0.000 | 0.001 | 0.913 | NA | NA | NA |
| rs7571789 | Hand grip strength (left) | myopia | 0.013 | 0.002 | 2.79E-14 | 0.000 | 0.001 | 0.881 | NA | NA | NA |
| rs7705189 | Hand grip strength (left) | myopia | 0.013 | 0.002 | 7.68E-14 | 0.000 | 0.001 | 0.749 | NA | NA | NA |
| rs7740107 | Hand grip strength (left) | myopia | -0.017 | 0.002 | 2.95E-17 | 0.003 | 0.001 | 0.000 | NA | NA | NA |
| rs77485342 | Hand grip strength (left) | myopia | 0.042 | 0.006 | 1.22E-10 | -0.001 | 0.002 | 0.646 | NA | NA | NA |
| rs7856625 | Hand grip strength (left) | myopia | -0.012 | 0.002 | 5.24E-12 | 0.001 | 0.001 | 0.174 | NA | NA | NA |
| rs79172804 | Hand grip strength (left) | myopia | -0.016 | 0.002 | 2.44E-12 | -0.003 | 0.001 | 0.000 | NA | NA | NA |
| rs7963801 | Hand grip strength (left) | myopia | -0.011 | 0.002 | 7.21E-10 | 0.000 | 0.001 | 0.526 | NA | NA | NA |
| rs8108461 | Hand grip strength (left) | myopia | 0.010 | 0.002 | 1.16E-08 | -0.001 | 0.001 | 0.225 | NA | NA | NA |
| rs823141 | Hand grip strength (left) | myopia | -0.013 | 0.002 | 6.27E-14 | 0.000 | 0.001 | 0.484 | NA | NA | NA |
| rs867633 | Hand grip strength (left) | myopia | -0.010 | 0.002 | 3.98E-08 | 0.000 | 0.001 | 0.776 | NA | NA | NA |
| rs934075 | Hand grip strength (left) | myopia | -0.011 | 0.002 | 1.09E-08 | 0.000 | 0.001 | 0.961 | NA | NA | NA |
| rs9371881 | Hand grip strength (left) | myopia | 0.010 | 0.002 | 1.81E-08 | 0.000 | 0.001 | 0.894 | NA | NA | NA |
| rs10210654 | Hand grip strength (right) | myopia | 0.013 | 0.002 | 2.05E-08 | 0.001 | 0.001 | 0.485 | NA | NA | NA |
| rs1043515 | Hand grip strength (right) | myopia | 0.013 | 0.002 | 1.02E-13 | 0.001 | 0.001 | 0.209 | NA | NA | NA |
| rs10798483 | Hand grip strength (right) | myopia | 0.016 | 0.002 | 1.84E-19 | 0.000 | 0.001 | 0.875 | NA | NA | NA |
| rs10799428 | Hand grip strength (right) | myopia | -0.015 | 0.002 | 7.39E-11 | 0.000 | 0.001 | 0.582 | NA | NA | NA |
| rs10846071 | Hand grip strength (right) | myopia | -0.017 | 0.002 | 2.43E-21 | -0.001 | 0.001 | 0.436 | NA | NA | NA |
| rs11236189 | Hand grip strength (right) | myopia | -0.017 | 0.002 | 4.72E-22 | 0.000 | 0.001 | 0.797 | NA | NA | NA |
| rs11243202 | Hand grip strength (right) | myopia | 0.013 | 0.002 | 6.04E-13 | -0.001 | 0.001 | 0.269 | NA | NA | NA |
| rs11669079 | Hand grip strength (right) | myopia | 0.013 | 0.002 | 7.11E-11 | 0.001 | 0.001 | 0.059 | NA | NA | NA |
| rs11813532 | Hand grip strength (right) | myopia | -0.010 | 0.002 | 3.94E-08 | 0.001 | 0.001 | 0.100 | NA | NA | NA |
| rs12127013 | Hand grip strength (right) | myopia | -0.015 | 0.003 | 1.27E-08 | 0.001 | 0.001 | 0.487 | NA | NA | NA |
| rs12361415 | Hand grip strength (right) | myopia | 0.011 | 0.002 | 6.24E-09 | 0.000 | 0.001 | 0.775 | NA | NA | NA |
| rs12452505 | Hand grip strength (right) | myopia | -0.014 | 0.003 | 1.61E-08 | -0.002 | 0.001 | 0.092 | NA | NA | NA |
| rs1245463 | Hand grip strength (right) | myopia | 0.010 | 0.002 | 1.20E-08 | 0.000 | 0.001 | 0.947 | NA | NA | NA |
| rs12562146 | Hand grip strength (right) | myopia | 0.014 | 0.003 | 8.79E-09 | 0.001 | 0.001 | 0.502 | NA | NA | NA |
| rs12598856 | Hand grip strength (right) | myopia | 0.010 | 0.002 | 4.78E-08 | 0.000 | 0.001 | 0.766 | NA | NA | NA |
| rs12708450 | Hand grip strength (right) | myopia | -0.013 | 0.002 | 3.17E-09 | 0.000 | 0.001 | 0.917 | NA | NA | NA |
| rs12790261 | Hand grip strength (right) | myopia | -0.026 | 0.003 | 3.12E-16 | 0.002 | 0.001 | 0.081 | NA | NA | NA |
| rs1280839 | Hand grip strength (right) | myopia | 0.010 | 0.002 | 4.65E-09 | -0.001 | 0.001 | 0.091 | NA | NA | NA |
| rs12914702 | Hand grip strength (right) | myopia | 0.012 | 0.002 | 3.89E-09 | 0.000 | 0.001 | 0.532 | NA | NA | NA |
| rs12917449 | Hand grip strength (right) | myopia | -0.013 | 0.002 | 1.58E-09 | -0.001 | 0.001 | 0.414 | NA | NA | NA |
| rs13017833 | Hand grip strength (right) | myopia | -0.027 | 0.005 | 1.18E-08 | 0.003 | 0.002 | 0.093 | NA | NA | NA |
| rs13040292 | Hand grip strength (right) | myopia | 0.012 | 0.002 | 1.30E-09 | -0.001 | 0.001 | 0.087 | NA | NA | NA |
| rs13107325 | Hand grip strength (right) | myopia | -0.031 | 0.003 | 3.07E-20 | -0.004 | 0.001 | 0.001 | NA | NA | NA |
| rs13150083 | Hand grip strength (right) | myopia | -0.011 | 0.002 | 2.60E-08 | 0.000 | 0.001 | 0.528 | NA | NA | NA |
| rs143384 | Hand grip strength (right) | myopia | 0.023 | 0.002 | 2.75E-38 | 0.001 | 0.001 | 0.045 | NA | NA | NA |
| rs1442883 | Hand grip strength (right) | myopia | -0.011 | 0.002 | 3.16E-08 | 0.000 | 0.001 | 0.873 | NA | NA | NA |
| rs150330307 | Hand grip strength (right) | myopia | -0.037 | 0.005 | 1.65E-13 | 0.001 | 0.002 | 0.616 | NA | NA | NA |
| rs1514665 | Hand grip strength (right) | myopia | 0.010 | 0.002 | 1.37E-08 | 0.000 | 0.001 | 0.889 | NA | NA | NA |
| rs1550115 | Hand grip strength (right) | myopia | 0.016 | 0.002 | 7.98E-16 | 0.001 | 0.001 | 0.188 | NA | NA | NA |
| rs1556659 | Hand grip strength (right) | myopia | 0.017 | 0.002 | 1.76E-21 | 0.000 | 0.001 | 0.501 | NA | NA | NA |
| rs17688916 | Hand grip strength (right) | myopia | -0.015 | 0.002 | 4.92E-11 | -0.003 | 0.001 | 0.000 | NA | NA | NA |
| rs1991431 | Hand grip strength (right) | myopia | 0.011 | 0.002 | 2.50E-09 | -0.002 | 0.001 | 0.006 | NA | NA | NA |
| rs200531 | Hand grip strength (right) | myopia | 0.013 | 0.002 | 3.86E-09 | 0.000 | 0.001 | 0.562 | NA | NA | NA |
| rs2194411 | Hand grip strength (right) | myopia | 0.015 | 0.003 | 1.75E-08 | 0.000 | 0.001 | 0.621 | NA | NA | NA |
| rs2265309 | Hand grip strength (right) | myopia | -0.010 | 0.002 | 7.55E-09 | 0.000 | 0.001 | 0.782 | NA | NA | NA |
| rs2273555 | Hand grip strength (right) | myopia | 0.011 | 0.002 | 2.70E-10 | -0.002 | 0.001 | 0.010 | NA | NA | NA |
| rs2431112 | Hand grip strength (right) | myopia | -0.011 | 0.002 | 2.20E-09 | 0.000 | 0.001 | 0.717 | NA | NA | NA |
| rs249516 | Hand grip strength (right) | myopia | 0.011 | 0.002 | 2.91E-09 | 0.000 | 0.001 | 0.830 | NA | NA | NA |
| rs2587505 | Hand grip strength (right) | myopia | -0.010 | 0.002 | 8.55E-09 | 0.000 | 0.001 | 0.956 | NA | NA | NA |
| rs28417075 | Hand grip strength (right) | myopia | -0.020 | 0.003 | 1.35E-09 | 0.000 | 0.001 | 0.776 | NA | NA | NA |
| rs2854152 | Hand grip strength (right) | myopia | 0.013 | 0.002 | 9.62E-13 | 0.000 | 0.001 | 0.521 | NA | NA | NA |
| rs2871865 | Hand grip strength (right) | myopia | -0.026 | 0.003 | 1.37E-21 | 0.000 | 0.001 | 0.768 | NA | NA | NA |
| rs2894602 | Hand grip strength (right) | myopia | 0.011 | 0.002 | 4.26E-08 | 0.000 | 0.001 | 0.651 | NA | NA | NA |
| rs2971154 | Hand grip strength (right) | myopia | -0.011 | 0.002 | 1.90E-09 | 0.000 | 0.001 | 0.520 | NA | NA | NA |
| rs3116605 | Hand grip strength (right) | myopia | -0.019 | 0.002 | 5.67E-19 | -0.001 | 0.001 | 0.266 | NA | NA | NA |
| rs34217742 | Hand grip strength (right) | myopia | 0.016 | 0.003 | 4.10E-09 | 0.001 | 0.001 | 0.304 | NA | NA | NA |
| rs34530577 | Hand grip strength (right) | myopia | -0.011 | 0.002 | 1.51E-09 | 0.000 | 0.001 | 0.715 | NA | NA | NA |
| rs34588175 | Hand grip strength (right) | myopia | -0.022 | 0.002 | 3.39E-19 | 0.000 | 0.001 | 0.741 | NA | NA | NA |
| rs34627176 | Hand grip strength (right) | myopia | -0.012 | 0.002 | 4.96E-08 | -0.001 | 0.001 | 0.136 | NA | NA | NA |
| rs35910339 | Hand grip strength (right) | myopia | -0.013 | 0.002 | 1.58E-11 | 0.001 | 0.001 | 0.112 | NA | NA | NA |
| rs3771498 | Hand grip strength (right) | myopia | 0.015 | 0.002 | 2.08E-17 | 0.000 | 0.001 | 0.656 | NA | NA | NA |
| rs3773853 | Hand grip strength (right) | myopia | 0.010 | 0.002 | 2.25E-08 | 0.002 | 0.001 | 0.008 | NA | NA | NA |
| rs3790076 | Hand grip strength (right) | myopia | -0.011 | 0.002 | 9.62E-11 | 0.000 | 0.001 | 0.804 | NA | NA | NA |
| rs417591 | Hand grip strength (right) | myopia | 0.014 | 0.002 | 2.83E-11 | 0.000 | 0.001 | 0.721 | NA | NA | NA |
| rs4308051 | Hand grip strength (right) | myopia | 0.016 | 0.002 | 2.91E-14 | 0.000 | 0.001 | 0.660 | NA | NA | NA |
| rs4326984 | Hand grip strength (right) | myopia | -0.010 | 0.002 | 3.65E-08 | 0.000 | 0.001 | 0.554 | NA | NA | NA |
| rs4373305 | Hand grip strength (right) | myopia | -0.011 | 0.002 | 4.74E-09 | 0.001 | 0.001 | 0.137 | NA | NA | NA |
| rs4380799 | Hand grip strength (right) | myopia | -0.014 | 0.002 | 1.48E-12 | 0.000 | 0.001 | 0.831 | NA | NA | NA |
| rs4594848 | Hand grip strength (right) | myopia | 0.014 | 0.002 | 2.45E-16 | 0.000 | 0.001 | 0.614 | NA | NA | NA |
| rs4621706 | Hand grip strength (right) | myopia | -0.010 | 0.002 | 8.81E-09 | 0.000 | 0.001 | 0.471 | NA | NA | NA |
| rs4730984 | Hand grip strength (right) | myopia | 0.012 | 0.002 | 1.01E-08 | 0.001 | 0.001 | 0.303 | NA | NA | NA |
| rs4751671 | Hand grip strength (right) | myopia | 0.011 | 0.002 | 1.30E-09 | 0.001 | 0.001 | 0.238 | NA | NA | NA |
| rs475390 | Hand grip strength (right) | myopia | -0.014 | 0.002 | 5.09E-11 | -0.001 | 0.001 | 0.117 | NA | NA | NA |
| rs4764131 | Hand grip strength (right) | myopia | -0.017 | 0.002 | 4.35E-22 | -0.001 | 0.001 | 0.459 | NA | NA | NA |
| rs4784329 | Hand grip strength (right) | myopia | -0.013 | 0.002 | 5.35E-14 | 0.000 | 0.001 | 0.575 | NA | NA | NA |
| rs4785574 | Hand grip strength (right) | myopia | -0.011 | 0.002 | 4.54E-10 | 0.000 | 0.001 | 0.657 | NA | NA | NA |
| rs4886778 | Hand grip strength (right) | myopia | 0.013 | 0.002 | 1.56E-13 | 0.002 | 0.001 | 0.007 | NA | NA | NA |
| rs4927015 | Hand grip strength (right) | myopia | 0.013 | 0.002 | 4.92E-13 | -0.001 | 0.001 | 0.069 | NA | NA | NA |
| rs4945185 | Hand grip strength (right) | myopia | -0.010 | 0.002 | 2.28E-08 | -0.001 | 0.001 | 0.358 | NA | NA | NA |
| rs56412116 | Hand grip strength (right) | myopia | -0.012 | 0.002 | 1.32E-08 | -0.001 | 0.001 | 0.536 | NA | NA | NA |
| rs57316347 | Hand grip strength (right) | myopia | -0.011 | 0.002 | 1.18E-08 | 0.001 | 0.001 | 0.140 | NA | NA | NA |
| rs6006984 | Hand grip strength (right) | myopia | 0.011 | 0.002 | 9.67E-09 | -0.002 | 0.001 | 0.020 | NA | NA | NA |
| rs635538 | Hand grip strength (right) | myopia | -0.024 | 0.003 | 1.88E-14 | 0.000 | 0.001 | 0.687 | NA | NA | NA |
| rs6425501 | Hand grip strength (right) | myopia | 0.010 | 0.002 | 3.08E-08 | 0.001 | 0.001 | 0.418 | NA | NA | NA |
| rs6539284 | Hand grip strength (right) | myopia | 0.012 | 0.002 | 1.17E-11 | 0.000 | 0.001 | 0.530 | NA | NA | NA |
| rs6693965 | Hand grip strength (right) | myopia | -0.016 | 0.003 | 4.93E-10 | -0.001 | 0.001 | 0.587 | NA | NA | NA |
| rs6977081 | Hand grip strength (right) | myopia | 0.013 | 0.002 | 1.39E-11 | 0.001 | 0.001 | 0.078 | NA | NA | NA |
| rs700518 | Hand grip strength (right) | myopia | 0.011 | 0.002 | 2.91E-10 | -0.001 | 0.001 | 0.094 | NA | NA | NA |
| rs7071654 | Hand grip strength (right) | myopia | 0.016 | 0.002 | 9.48E-11 | 0.000 | 0.001 | 0.813 | NA | NA | NA |
| rs7206195 | Hand grip strength (right) | myopia | -0.018 | 0.002 | 2.60E-15 | 0.000 | 0.001 | 0.771 | NA | NA | NA |
| rs721101 | Hand grip strength (right) | myopia | 0.012 | 0.002 | 4.60E-10 | 0.001 | 0.001 | 0.390 | NA | NA | NA |
| rs7249081 | Hand grip strength (right) | myopia | 0.011 | 0.002 | 1.47E-09 | 0.000 | 0.001 | 0.878 | NA | NA | NA |
| rs73307079 | Hand grip strength (right) | myopia | 0.014 | 0.002 | 4.55E-10 | 0.000 | 0.001 | 0.554 | NA | NA | NA |
| rs74265413 | Hand grip strength (right) | myopia | 0.012 | 0.002 | 1.51E-11 | 0.000 | 0.001 | 0.856 | NA | NA | NA |
| rs75069534 | Hand grip strength (right) | myopia | 0.017 | 0.003 | 3.28E-09 | -0.001 | 0.001 | 0.547 | NA | NA | NA |
| rs7740107 | Hand grip strength (right) | myopia | -0.017 | 0.002 | 8.50E-18 | 0.003 | 0.001 | 0.000 | NA | NA | NA |
| rs77485342 | Hand grip strength (right) | myopia | 0.042 | 0.006 | 1.10E-10 | -0.001 | 0.002 | 0.646 | NA | NA | NA |
| rs7760564 | Hand grip strength (right) | myopia | 0.012 | 0.002 | 2.09E-08 | 0.000 | 0.001 | 0.533 | NA | NA | NA |
| rs7871404 | Hand grip strength (right) | myopia | 0.012 | 0.002 | 2.70E-08 | 0.001 | 0.001 | 0.398 | NA | NA | NA |
| rs7968902 | Hand grip strength (right) | myopia | -0.013 | 0.002 | 2.94E-12 | 0.001 | 0.001 | 0.336 | NA | NA | NA |
| rs8012800 | Hand grip strength (right) | myopia | 0.011 | 0.002 | 4.32E-08 | 0.001 | 0.001 | 0.356 | NA | NA | NA |
| rs8055199 | Hand grip strength (right) | myopia | -0.011 | 0.002 | 1.21E-09 | 0.000 | 0.001 | 0.904 | NA | NA | NA |
| rs817316 | Hand grip strength (right) | myopia | 0.010 | 0.002 | 5.99E-09 | -0.001 | 0.001 | 0.157 | NA | NA | NA |
| rs823130 | Hand grip strength (right) | myopia | -0.015 | 0.002 | 1.37E-16 | 0.000 | 0.001 | 0.650 | NA | NA | NA |
| rs9267806 | Hand grip strength (right) | myopia | -0.019 | 0.002 | 3.02E-20 | 0.000 | 0.001 | 0.887 | NA | NA | NA |
| rs9322822 | Hand grip strength (right) | myopia | 0.012 | 0.002 | 3.74E-10 | 0.000 | 0.001 | 0.697 | NA | NA | NA |
| rs934075 | Hand grip strength (right) | myopia | -0.011 | 0.002 | 5.54E-09 | 0.000 | 0.001 | 0.961 | NA | NA | NA |
| rs9396861 | Hand grip strength (right) | myopia | -0.011 | 0.002 | 9.65E-10 | 0.000 | 0.001 | 0.933 | NA | NA | NA |
| rs973767 | Hand grip strength (right) | myopia | 0.014 | 0.002 | 7.97E-09 | 0.000 | 0.001 | 0.773 | NA | NA | NA |
| rs9847951 | Hand grip strength (right) | myopia | -0.010 | 0.002 | 2.32E-08 | 0.001 | 0.001 | 0.245 | NA | NA | NA |
| rs997850 | Hand grip strength (right) | myopia | -0.010 | 0.002 | 1.57E-08 | 0.000 | 0.001 | 0.589 | NA | NA | NA |

**CAD: coronary artery disease;**
